# Age Distribution of All-Cause Mortality Among Children Younger Than 5 Years in Low- and Middle-Income Countries

**DOI:** 10.1001/jamanetworkopen.2022.12692

**Published:** 2022-05-19

**Authors:** Omar Karlsson, Rockli Kim, Andreas Hasman, S. V. Subramanian

**Affiliations:** 1 Takemi Program in International Health, Harvard T. H. Chan School of Public Health, Harvard University, Boston, Massachusetts; 2 Division of Health Policy and Management, College of Health Science, Korea University, Seoul, South Korea; 3 Interdisciplinary Program in Precision Public Health, Department of Public Health Sciences, Graduate School of Korea University, Seoul, South Korea; 4 Harvard Center for Population and Development Studies, Cambridge, Massachusetts; 5 UNICEF Programme Division, New York, New York; 6 Department of Social and Behavioral Sciences, Harvard T. H. Chan School of Public Health, Harvard University, Boston, Massachusetts

## Abstract

**Importance:**

Coverage of essential child health and nutrition interventions in low- and middle-income countries remains suboptimal. Adverse exposures, such as undernutrition and infections, are particularly harmful during the 1000 days from conception until 2 years of age.

**Objective:**

To investigate whether deaths in children younger than 5 years—which also reflect adverse exposures faced by children more broadly—are concentrated in the first 2 years after birth.

**Design, Setting, and Participants:**

This cross-sectional study used a synthetic cohort probability method with Demographic and Health Surveys and Multiple Indicator Cluster Surveys from 77 low- and middle-income countries, with the earliest survey starting data collection in March 2010 and the most recent survey ending data collection in December 2019. Participants included 2 827 515 children who were younger than 5 years at any point 10 years before survey. Data were analyzed from March 11 to 21, 2022.

**Main Outcomes and Measures:**

Share of deaths among children younger than 5 years occurring before 1 month, at 1 to 23 months, and at 24 to 59 months of age.

**Results:**

Among the 2 827 515 children included in the analysis, 81.5% (95% CI, 81.0%-82.0%) of deaths occurred in the first 2 years after birth ranging from 63.7% (95% CI, 61.6%-65.7%) in Niger to 97.8% (95% CI, 85.9%-99.7%) in Albania. An estimated 18.5% (95% CI, 18.0%-19.0%) of child deaths occurred at 24 to 59 months of age. Countries with higher mortality rates among children younger than 5 years had a lower share of deaths occurring in the neonatal period.

**Conclusions and Relevance:**

In this sample of 77 low- and middle-income countries, a large majority of deaths among children younger than 5 years occurred before 2 years of age in all countries among boys and girls and in households with the worst and best living standards. Research has highlighted perinatal complications, infections, and undernutrition as primary causes of death among children younger than 5 years. Therefore, coverage of interventions to reduce these adverse exposures should be ensured during pregnancy and the first 2 years after birth, which is also a crucial period for human development.

## Key Points

**Question:**

What share of deaths among children younger than 5 years occur before 2 years of age in low- and middle-income countries?

**Findings:**

In this cross-sectional study of 2 827 515 children younger than 5 years from 77 countries, a large majority (81.5%) of deaths occurred before 2 years of age in all countries, among boys and girls, and in the households with the highest and lowest living standards.

**Meaning:**

These findings suggest that coverage of potentially life-saving interventions should be ensured for children younger than 2 years of age in low- and middle-income countries.

## Teaser

This cross-sectional study investigates all-cause mortality among children younger than 5 years in 77 low- and middle-income countries and compares the mortality rates among children younger than 2 years with those aged 2 to 4 years.

## Introduction

Globally, more than 5 million children younger than 5 years die each year, mostly owing to preventable causes.^1^ The burden is highly unequal: almost 2 million of these deaths occurred in the 47 countries making up the United Nations’ list of least developed countries according to socioeconomic development, leading to a mortality rate of 61 per 1000 live births, whereas about 60 000 occurred in high-income countries, with a mortality rate of 5 deaths per 1000 live births.^1^ The mortality rate among children younger than 5 years is a crucial indicator of children’s health and a composite measure of adverse exposures. Most deaths among these children are attributed to prematurity, birth asphyxia and trauma, and infections, particularly those causing pneumonia, diarrhea, and malaria.^2^ Undernutrition has also been estimated to be an underlying factor in 45% of all deaths among children younger than 5 years (in 2011).^3^ Infections and undernutrition are especially detrimental from conception until 2 years of age—the first 1000 days—with immediate negative consequences for survival, physical growth, and cognitive development, as well as long-term consequences, including for productivity, wages, and poverty reduction.^4,5^

Globally, deaths among children younger than 5 years are increasingly concentrated in infancy, especially the neonatal period.^6^ However, given the importance of the first 1000 days, reporting the mortality rate of children younger than 2 years, rather than only that of infants, is important for communicating adversity faced by the most vulnerable children. In this study, we investigated mortality of children younger than 2 years and compared it with the mortality of children aged 2 to 4 years in 77 low- and middle-income countries, 37 of which were on the United Nations’ list of least developed countries.^7^ We further separated the share of deaths occurring before 2 years of age into neonatal deaths (approximated as the first month after birth) and deaths occurring at ages 1 to 23 months. We show all estimates for the pooled sample, least developed countries, regions, World Bank income groups, and countries and further stratified by sex and household living standards.

Because mortality is a composite and readily available measure for child health, primarily reflecting prenatal nutrition, prematurity, and perinatal complications in the neonatal period and malnutrition and infections after the neonatal period, understanding how it is patterned by age will indicate when children experience the most consequential adverse exposures. Further, understanding differences in age patterning by location, sex, and living standards will also help to ensure equitable interventions targeting.

## Methods

### Data

We used Demographic and Health Surveys (DHS) and Multiple Indicator Cluster Surveys (MICS), which are nationally representative cross-sectional household surveys conducted regularly in low- and middle-income countries.^8,9,10,11,12^ For each country, the most recent survey was used. Countries with no survey after 2010 were excluded. The most recent survey was conducted in 2019. We only included surveys that were nationally representative and collected complete birth histories that included date of birth and age at death, allowing direct estimation of mortality rates. A total of 77 countries (50 from the DHS and 27 from MICS) met these criteria. Data collection for the earliest survey started in March 2010 and data collection for the most recent survey ended in December 2019. We restricted the analysis to the 120 months preceding the survey and excluded children born in the survey month. We obtained a sample of 2 827 515 children younger than 5 years at any point during that period (eTable 1 in the Supplement). This study followed the Strengthening the Reporting of Observational Studies in Epidemiology (STROBE) reporting guideline. The institutional review board (IRB) of Harvard Longwood Campus allows researchers to self-determine when an IRB application is required using an IRB decision tool. Because this study used publicly accessible secondary data obtained from the DHS and MICS websites, it did not meet the regulatory definition of human subjects research, thus IRB review was not required.

Stratified multistage sampling was used. Strata were based on geographic or administrative subnational regions. The primary sampling units consisted of census enumeration areas, generally neighborhoods in urban areas and villages in rural areas. Primary sampling units were sampled with a probability proportional to size. Approximately 20 to 30 households were sampled (using systematic random sampling) for an interview in the selected primary sampling units. Several questionnaires were administered. The woman’s questionnaire^13,14^ recorded information from all women aged 15 to 49 years on various topics, including birth histories recording all their live births. A household questionnaire^15,16^ recorded basic information on the household and its members. Data from other questionnaires were not used in this study.

The sample sizes for each survey were intended to be sufficient to yield precise estimates for health of children younger than 5 years and their mothers at the population level and for subnational regions.^10,17^ Response rates of greater than 90% were typically observed in these surveys.^8,10^ Nonresponses were not replaced. Sampling weights, defined as the inverse probability of being included in the survey, were included with the data to adjust for nonresponses and oversampling and improve precision.^10,18^ No observations were missing for the variables used in this study; however, date of birth and age at death were imputed by the DHS and MICS when missing or incomplete.^18,19^

We restricted the period under study to 120 months before each survey to obtain a large enough sample size for reliable rates for subgroups (ie, by living standards and sex) while limiting recall bias and bias due to maternal age censoring (eg, at 180 months before the survey, only births to women younger than 35 years at the time of the survey were included).^20^ We also show results using the 5-year period before survey in eTables 2 and 3 and eFigures 1 to 4 in the Supplement, which yields similar age distribution of deaths as our main results. All data analyses were performed using Stata, version 16 (StataCorp LLC).

### Statistical Analysis

Data were analyzed from March 11 to 21, 2022. Mortality rates were constructed using data from the birth histories in the woman’s questionnaire, which included all live births by women aged 15 to 49 years at the time of the survey. A period of 120 months before the survey was used. The month of the survey was excluded because it did not contribute a whole month. Three variables were used to construct the mortality rate: month of interview, month of birth, and—for deceased children—age at death (in months).

Mortality rates were estimated using the same approach used in the DHS and MICS reports: a synthetic cohort life-table approach^18,21^ (further details are provided in eMethods 1, eTable 4, and eFigure 5 in the Supplement). Mortality for children aged 1 to 23 months was calculated by subtracting the neonatal mortality rate from the mortality rate of children aged 0 to 23 months in the same way as neonatal mortality rate is subtracted from the infant mortality rate to construct postneonatal mortality rate in the reports from the DHS.^18^ Similarly, mortality for children aged 24 to 59 months was calculated by subtracting the mortality rate for children aged 0 to 23 months from the mortality rate of children younger than 5 years. Therefore, the sum of the mortality measures for neonates, children aged 1 to 23 months, and children aged 24 to 59 months equals the mortality rate for children younger than 5 years. The share measures were then calculated by dividing the measures for mortality at 0 to 23 months of age, younger than 1 month, 1 to 23 months of age, and 24 to 59 months of age by mortality rate for children younger than 5 years and were presented as percentage of deaths among children younger than 5 years occurring in each age group.

Our primary results show the mortality rate for children younger than 5 years and the share of these deaths occurring before 1 month (neonates), 1 to 23 months, and 24 to 59 months of age. We show the Pearson correlations between the mortality rate among children younger than 5 years and the share of deaths occurring in each age group in eFigure 6 in the Supplement. We also show the percentage of deaths among children younger than 5 years occurring in each age interval used for the synthetic cohort probability method (ie, <1, 1-2, 3-5, 6-11, 12-23, 24-35, 36-47, and 48-59 months).

We further show the share of deaths among children younger than 5 years in each age group for boys and girls and children in 20% of households with the best and worst living standards within countries according to a wealth index provided by the DHS and MICS (further details are provided in eMethods 2 in the Supplement). We tabulated all mortality measures and shares and differences by sex and living standards with 95% CIs (eTables 5 to 10 in the Supplement).

All estimates were weighted using sampling weights scaled such that the sum of the weights for children who were younger than 5 years at any point in the 120 months before the survey equaled the number of births (in thousands) 10 years before each survey. Although children included were born as much as 180 months before survey, some of these children may have died before entering the 120 months before survey. The Population Division of the United Nations provides data on the number of births for 5-year periods: we added two 5-year periods and interpolated intervening years to obtain a yearly time series that was then linked to the survey year in each country.^22^ The 95% CIs were based on bootstrapped SEs using 50 sample replications of the primary sampling units. The 95% CIs were logit transformed (after converting estimates to fractions) to keep them within possible bounds for mortality (ie, 0-1000) and shares (ie, 0-100). Two-sided *P* < .05 indicated statistical significance for Pearson correlation coefficients.

## Results

**Figure 1.**
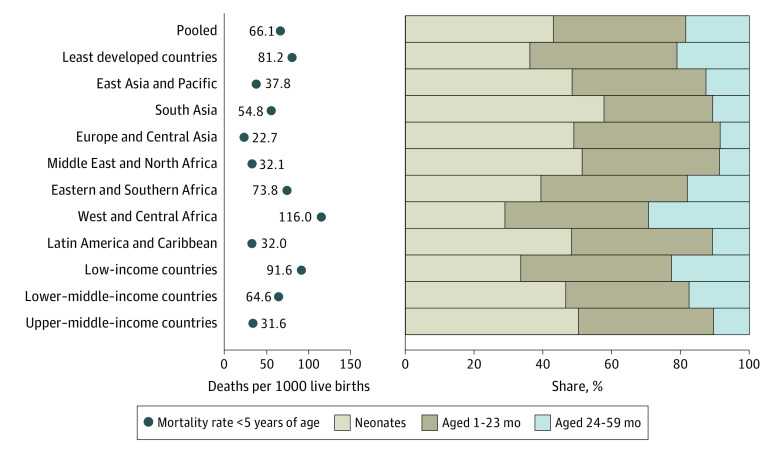
Mortality Rate Among Children Younger Than 5 Years and Share of These Deaths Occurring at Different Ages Tabulated estimates and confidence intervals are provided in eTable 5 in the Supplement.

Among the 2 827 515 children included in the analysis, we observed a mortality rate among children younger than 5 years of 66.1 (95% CI, 65.1-67.1) deaths per 1000 live births in our pooled sample (Figure 1 and eTable 5 in the Supplement) and 81.2 (95% CI, 79.6-82.8) in the least developed countries. Regional mortality rates (using UNICEF regional classifications^7^) were as follows: Europe and Central Asia, 22.7 (95% CI, 19.8-26.0); Middle East and North Africa, 32.1 (95% CI, 30.3-33.9); Latin America and Caribbean, 32.0 (95% CI, 30.5-33.6); East Asia and Pacific, 37.8 (95% CI, 35.8-39.8); South Asia, 54.8 (95% CI, 53.6-56.1); Eastern and Southern Africa, 73.8 (95% CI, 71.7-76.0); and West and Central Africa, 116.0 (95% CI, 113.2-118.7). The share of deaths among children younger than 5 years occurring in the first 2 years of life was 81.5% (95% CI, 81.0%-82.0%) in our pooled sample, 43.0% (95% CI, 42.4%-43.7%) in the neonatal period, 38.5% (95% CI, 37.9%-39.1%) at 1 to 23 months of age, and 18.5% (95% CI, 18.0-19.0) at 2 years or older. In the least developed countries, 79.0% (95% CI, 78.4%-79.6%) of deaths among children younger than 5 years occurred before 2 years of age. Regionally, this share was as follows: West and Central Africa, 70.7% (95% CI, 69.9%-71.4%); Eastern and Southern Africa, 82.0% (95% CI, 81.0%-82.9%); East Asia and Pacific, 87.4% (95% CI, 85.7%-88.9%); South Asia, 89.4% (95% CI, 88.9%-89.8%), Latin America and Caribbean, 89.3% (95% CI, 87.9%-90.4%); Middle East and North Africa, 91.3% (95% CI, 90.0%-92.5%); and Europe and Central Asia, 91.5% (95% CI, 87.5%-94.4%).

**Figure 2.**
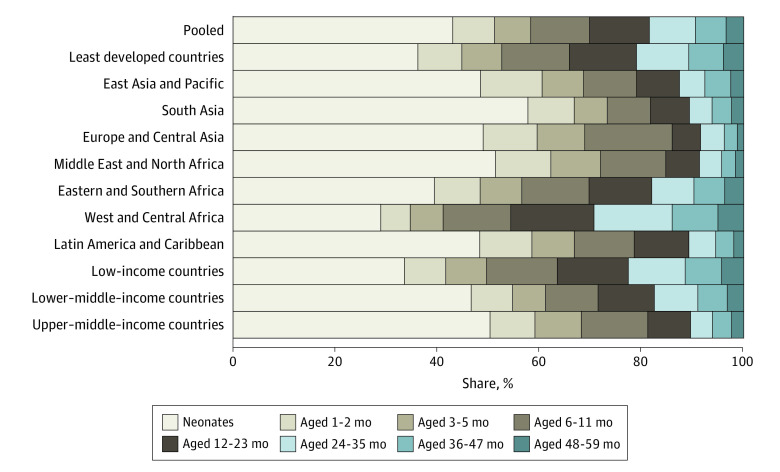
Share of Deaths Among Children Younger Than 5 Years Occurring at Different Ages Using Detailed Age Intervals Tabulated estimates and 95% CIs are provided in eTable 6 in the Supplement. Estimates for countries are provided in eFigure 7 in the Supplement.

Looking at the age distribution of deaths by World Bank income groups shows a gradient in which the share of deaths among children younger than 5 years that occurred before 2 years of age ranged from 77.4% (95% CI, 76.7%-78.1%) in low-income countries to 89.6% (95% CI, 88.3%-90.7%) in upper–middle-income countries. Breaking the age groups into more detailed age intervals shows that in the pooled sample, 8.2% (95% CI, 7.9%-8.5%) of deaths occurred at 1 to 2 months of age, 7.1% (95% CI, 6.8%-7.4%) at 3 to 5 months of age, 11.5% (95% CI, 11.2%-11.9%) at 6 to 11 months of age, 11.7% (95% CI, 11.4%-12.1%) at 12 to 23 months of age, 9.0% (95% CI, 8.6%-9.4%) at 24 to 35 months of age, 6.0% (95% CI, 5.8%-6.2%) at 36 to 47 months of age, and 3.5% (95% CI, 3.3%-3.7%) at 48 to 59 months of age (Figure 2 and eTable 6 in the Supplement). West and Central Africa had 15.3% (95% CI, 14.7%-15.9%) of deaths among children younger than 5 years occurring at age 24 to 35 months, and Eastern and Southern Africa had 8.3% (95% CI, 7.8%-8.8%).

**Figure 3.**
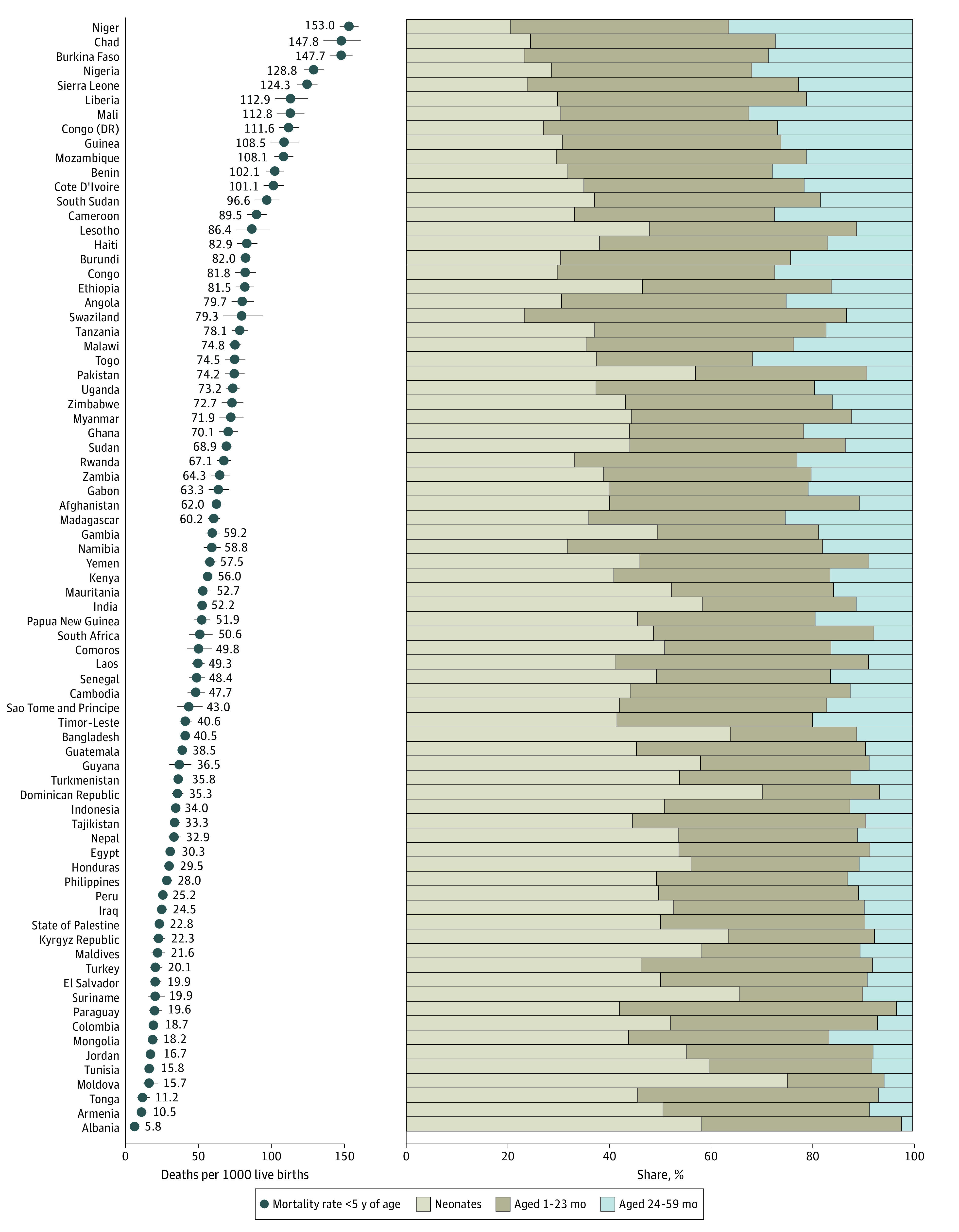
Mortality Rate Among Children Younger Than 5 Years and Share of These Deaths Occurring at Different Ages by Countries Error bars indicate 95% CIs (some of which may be narrower than symbols). Tabulated estimates and 95% CIs are provided in eTable 5 in the Supplement.

Figure 3, in which countries are ranked according to mortality rate among children younger than 5 years, shows a pattern: Countries with a higher mortality rate among children younger than 5 years have a lower share of deaths at 0 to 23 months of age—driven by a lower percentage of deaths in the neonatal period—and a larger share of deaths at 24 to 59 months of age (see also eTable 5 in the Supplement). This pattern is highlighted by the Pearson correlations for the mortality rate among children younger than 5 years, which were 0.82 (*P* < .001) for share of these deaths occurring at 24 to 59 months of age, 0.40 (*P* < .001) for the share occurring at 1 to 23 months of age, and −0.80 (*P* < .001) for the share occurring before 1 month of age (eFigure 6 in the Supplement). For example, Niger, the country with the highest mortality rate among children younger than 5 years (153.0 [95% CI, 146.7-159.6] deaths per 1000 live births), had the lowest share of these deaths at ages 0 to 23 months at 63.7% (95% CI, 61.6%-65.7%). The greatest share of deaths among children younger than 5 years occurring before 2 years of age, at 97.8% (95% CI, 85.9%-99.7%), was observed in Albania, which also had the lowest mortality rate among children younger than 5 years (5.8 [95% CI, 3.7-9.0] deaths per 1000 live births).

Most countries had a greater share of deaths among children younger than 5 years occurring in the second compared with the third year (eFigure 7 and eTable 6 in the Supplement). Many countries in West and Central Africa and a few in Eastern and Southern Africa were notable examples of countries with large and similar shares of deaths at 2 and 3 years of age. Niger had the greatest share of deaths occurring at 3 years of age (19.7% [95% CI, 18.2%-21.2%]).

### Results by Household Living Standards and Sex

**Figure 4.**
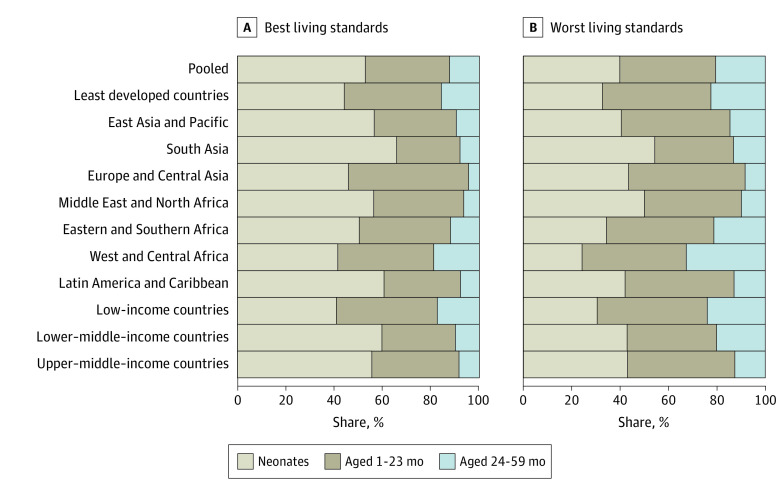
Share of Deaths Among Children Younger Than 5 Years Occurring at Different Ages by Living Standards Worst indicates the 20% of households with the worst living standards within each country; best, the 20% of households with the best living standards within each country. Tabulated estimates and 95% CIs are provided in eTables 7 and 8 in the Supplement. Estimates for countries are provided in eFigure 8 in the Supplement.

In our pooled sample, children in the bottom 20% of households according to living standards within countries had a lower share of deaths among children younger than 5 years occurring before 2 years of age by 8.2 (95% CI, –9.2 to –7.3) percentage points compared with the 20% of households with the best living standards (Figure 4 and eTables 7 and 8 in the Supplement for mortality and shares, respectively). Children in households with the worst living standards had a smaller share of deaths occurring in the neonatal period (13.0 [95% CI, –15.4 to –10.5] percentage points) and a greater share of deaths occurring at ages 1 to 23 months (4.8 [95% CI, 2.3-7.2] percentage points) and 24 to 59 months (8.2 [95% CI, 7.3-9.2] percentage points). In all countries, fewer deaths occurred after 2 years of age than before 2 years of age, both for children with the worst and best living standards (eFigure 8 and eTables 7 and 8 in the Supplement). However, several groups had zero deaths in our sample.

**Figure 5.**
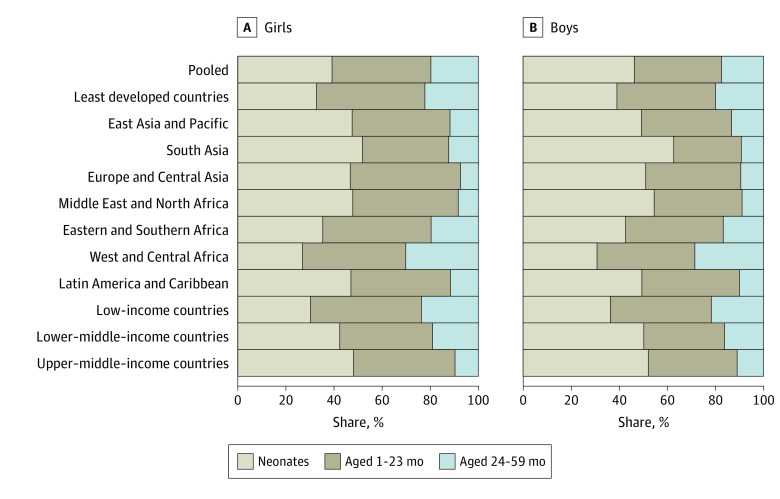
Share of Deaths Among Children Younger Than 5 Years Occurring at Different Ages by Sex Tabulated estimates and 95% CIs are provided in eTables 9 and 10 in the Supplement. Estimates for countries are provided in eFigure 9 in the Supplement.

In our pooled sample, boys had a higher neonatal mortality rate, or 8.0 (95% CI, 7.1-8.8) more deaths per 1000 live births, whereas no difference was observed in mortality by sex after the neonatal period, both at 1 to 23 months (–0.2 [95% CI, –0.9 to 0.6]) and 24 to 49 months (–0.1 [95% CI, –0.6 to 0.4]) of age (eTable 9 in the Supplement). In our pooled sample, the share of deaths among children younger than 5 years occurring in the neonatal period was 7.1 (95% CI, 6.1-8.0) percentage points greater for boys than girls (Figure 5 and eTable 10 in the Supplement). Boys had a lower share of deaths at 1 to 23 months of age by 4.8 (95% CI, –5.6 to –4.0) percentage points and at ages 24 to 59 months by 2.3 (95% CI, –2.9 to –1.6) percentage points (eFigure 9 and eTables 9 and 10 in the Supplement present results by country).

## Discussion

This cross-sectional study had 2 salient findings. First, 81.5% of deaths among children younger than 5 years occurred before 2 years of age in our pooled sample of 77 low- and middle-income countries—43.0% in the neonatal period and 38.5% at ages 1 to 23 months—whereas 18.5% were among children 2 years and older. Second, countries with a higher mortality rate among children younger than 5 years had a smaller proportion of these deaths in the neonatal period, presumably because settings with higher mortality have a relatively high burden of infectious diseases and undernutrition, which are more common causes of death after the neonatal period. However, regardless of the mortality profile among children younger than 5 years, most child deaths occurred before 2 years of age in all countries, ranging from 63.7% in Niger to 97.8% in Albania.

We observed other important differences in the age pattern of deaths among children younger than 5 years: Children in households with lower living standards within countries had a greater share of these deaths occurring after the neonatal period. Boys had a higher share of deaths among children younger than 5 years in the neonatal period and a lower share at ages 1 to 23 months, whereas the overall sex difference in the share occurring at ages 2 to 4 years was small.

Child deaths reflect childhood adversity more broadly in that the perinatal complications, undernutrition, and infections leading to deaths also have long-term adverse effects on the survivors. Undernutrition and infections while in utero and 2 years after birth have been suggested to cause permanent damage to physical growth, health, and cognitive development and have even been linked to worse health and lower income and educational attainment in adulthood.^4,5^ However, some processes should be considered when interpreting these findings as indicative of the harmfulness of exposures specifically occurring before and after children turn 2 years of age. The mortality of children aged 2 to 4 years will also be caused by previous exposures that occur as early as pregnancy. Therefore, the mortality rate of children aged 2 to 4 years cannot be interpreted as only reflecting exposures during that age period but as a part of an accumulation of exposures that eventually cause death.^23^ There was also an obvious difference in exposure time (ie, 2 vs 3 years); however, in our pooled sample, we also observed that 11.7% of deaths occurred at ages 12 to 23 months, whereas 9.0% occurred at 2 years of age, 6.0% at 3 years of age, and 3.5% at 4 years of age. Accumulation of adverse exposures and differences in exposure time would mean that the vulnerability of children aged 2 to 4 years would be exaggerated when looking at the shares of deaths among children younger than 5 years occurring in that period. Accidents also become a more common cause of death as children age, although undernutrition and infections remain more common causes before 5 years of age.^2^ Further, this study covers a period as early as 2000, and because the concentration of deaths in the neonatal period was greater where mortality rates among children younger than 5 years were lower, the share of these deaths occurring after children are 2 years of age is probably lower in more recent periods in which the mortality rate among children younger than 5 years has declined.^1^

### Implications for Policy and Practice

Almost 50% of neonatal deaths have been attributed to preterm birth and birth asphyxia and trauma, with many deaths also attributed to neonatal infections and disorders.^2^ Approximately 50% of deaths among children younger than 5 years after the neonatal period have been attributed to lower respiratory tract infections, diarrhea, and malaria (primarily in sub-Saharan Africa), with many deaths also attributed to congenital heart anomalies, meningitis, whooping cough, and undernutrition.^2^ However, deaths among children younger than 5 years often have multiple causal components associated with chronic undernutrition and repeated infections.^23^ Further, separating deaths caused by undernutrition and infections is difficult: Infections cause undernutrition by restricting the absorption of nutrients, increasing energy requirements to fight off infections, and reducing appetite.^24^ Meanwhile, undernourished children are more susceptible to infections and more likely to die of them owing to weakened immune systems.^24^ A study from 2013^3^ indicated that undernutrition—including suboptimal breastfeeding, stunting, wasting, and vitamin A, zinc, and micronutrient deficiencies—was an underlying factor in 45% of child deaths. Another study that used data from 2004^25^ indicated that undernutrition in children younger than 5 years contributed to 73% of deaths due to diarrhea, 44% of deaths due to pneumonia, 11% of deaths due to prematurity, 5% of deaths due to birth asphyxia and trauma, and 45% of deaths due to severe neonatal infections.

The evidence further points toward the effectiveness of nutrition interventions and complementary feeding to improve child health, particularly at young ages.^26,27,28,29,30,31,32,33^ Many deaths can also be prevented by reducing environmental contaminants, particularly those causing diarrhea due to inadequate drinking water, sanitation, and hygiene^34^ and pneumonia due to indoor air pollution from solid cooking fuels.^35^ In addition, preventing deaths—especially in the neonatal period—also requires improved access to high-quality health care, such as antenatal, birth, and postnatal care for mothers and children.^36,37^ For example, only 64% and 77% of births in sub-Saharan Africa and South Asia, respectively, were attended by a skilled professional, and approximately one-half received the recommended 4 or more antenatal care visits.^38^ Variations in fertility and maternal age at birth can also influence the age distribution of deaths: Children of younger mothers are at a higher risk of death at younger than 5 years, particularly in infancy.^39,40,41^ The high fertility regions, where age at first birth also tends to be low—Eastern and Southern Africa and West and Central Africa^42^—would, therefore, have more deaths of younger children attributed to high fertility and young maternal age at birth.

This study shows that mortality risks are concentrated in the first 2 years after birth, with more than 4 of 5 deaths among children younger than 5 years occurring in this period in our sample. Health and nutrition interventions must ensure coverage of children at most risk. Governments could consider the age distribution of deaths among children younger than 5 years to guide context-specific decisions. Furthermore, where age-disaggregated mortality data are unavailable, the mortality rate among children younger than 5 years could be considered a proxy for the age distribution of deaths.^43^ However, other context-specific factors should also be considered in any prioritization, such as cohort- and period-specific events, the marginal cost of delivery, different adversities faced by children, and ongoing interventions, as well as variation between groups.

### Limitations

This study has limitations. First, we relied on survey data, which may have included inaccuracies. Dates of birth and death were often recorded from mothers by memory and may be subject to recall bias. Incomplete date of birth and age at death required imputation, which may be subject to error. Age at death reported in years was truncated to months, which—together with digit preference—resulted in age heaping, particularly at 12 months but also at 24 months of age. Because age at death referred to completed months, deaths at 24 months of age were classified as occurring after rather than before 2 years of age in the same way as deaths at 12 months of age are considered infant deaths in the DHS reports. When using a synthetic cohort probability method (as we did in this report), age heaping at 24 months may negatively bias deaths before 2 years and positively bias deaths after 2 years, exaggerating the share of deaths among children younger than 5 years for those 2 years and older. However, the DHS maintains that using the synthetic cohort probability method with age heaping is unlikely to bias the estimates more than 5% in the case of infant and child mortality, and even less for mortality among children younger than 2 and younger than 5 years, which were of interest in this study.^18^ Misreporting of age at birth could also cause problems for mortality rates; however, it is unlikely to have a large impact unless misreporting happens to a very different extent for surviving and deceased children.^18^ Further, the quality of age reporting has been suggested to have vastly improved in more recent DHS surveys, such as those used in this study.^44^ Omission of births from birth histories—especially of children who died—has also been suggested, mainly in older surveys.^45^ The DHS and MICS are some of the most commonly used and most reliable sources of mortality data for children younger than 5 years in low- and middle-income countries that lack vital registration systems.^46^

## Conclusions

This cross-sectional study found that most deaths among children younger than 5 years occurred before 2 years of age, and for these deaths, more than one-half occurred in the neonatal period. These results highlight interventions at the pregnancy and perinatal stages to reduce neonatal deaths, including addressing maternal malnutrition and infections and providing essential health care services. After the neonatal period, nutrition interventions and preventive and curative interventions for diarrhea and pneumonia are needed. Because most deaths among children younger than 5 years after the neonatal period are concentrated before 2 years of age, this group should receive special attention. Furthermore, ensuring that nutritional and health interventions cover all children younger than 2 years and pregnant women is crucial because the consequences of adverse exposure to undernutrition and infections during the 1000 days from conception have been shown to be particularly detrimental to health and human development more broadly.

## Notes

Supplement. **eMethods 1.** Constructing Mortality Measures **eMethods 2.** Measure for Household Living Standards **eTable 1.** Information on Data **eTable 2.** Mortality and the Share of Deaths Among Children Younger Than 5 Years by Age: Period Restricted to 5 Years Before Survey Instead of 10 Years **eTable 3.** Share of Deaths Among Children Younger Than 5 Years Occurring at Different Ages Using Detailed Age Intervals: Period Restricted to 5 Years Before Survey Instead of 10 Years **eTable 4.** Component Mortality Probabilities for Pooled Sample **eTable 5.** Mortality and the Share of Deaths Among Children Younger Than 5 Years by Age **eTable 6.** Share of Deaths Among Children Younger Than 5 Years Occurring at Different Ages Using Detailed Age Intervals **eTable 7.** Mortality (Deaths per 1000) at Different Ages by Living Standards **eTable 8.** Shares of Deaths Among Children Younger Than 5 Years Occurring at Different Ages by Living Standards **eTable 9.** Mortality (Deaths per 1000) at Different Ages by Sex **eTable 10.** Shares of Deaths Among Children Younger Than 5 Years Occurring at Different Ages by Sex **eFigure 1.** Mortality Rate Among Children Younger Than 5 Years and Share of These Deaths Occurring at Different Ages: Pooled, Least Developed Countries, Regions, and World Bank Income Groups: Period Restricted to 5 Years Before Survey Instead of 10 Years **eFigure 2.** Share of Deaths Among Children Younger Than 5 Years Occurring at Different Ages Using Detailed Age Intervals: Pooled, Least Developed Countries, Regions, and World Bank Income Groups: Period Restricted to 5 Years Before Survey Instead of 10 Years **eFigure 3.** Mortality Rate Among Children Younger Than 5 Years and Share of These Deaths Occurring at Different Ages by Country: Period Restricted to 5 Years Before Survey Instead of 10 Years **eFigure 4.** Share of Deaths Among Children Younger Than 5 Years Occurring at Different Ages Using Detailed Age Intervals by Countries: Period Restricted to 5 Years Before Survey Instead of 10 Years **eFigure 5.** Lexis Diagram Demonstrating Inclusion for Each Component Mortality Probability **eFigure 6.** Country-Level Association Between Mortality Rate Among Children Younger Than 5 Years and Share of These Deaths Occurring at Different Ages **eFigure 7.** Share of Deaths Among Children Younger Than 5 Years Occurring at Different Ages Using Detailed Age Intervals by Countries **eFigure 8.** Share of Deaths Among Children Younger Than 5 Years Occurring at Different Ages: Countries by Living Standards **eFigure 9.** Share of Deaths Among Children Younger Than 5 Years Occurring at Different Ages: Countries by Sex
eReferences
