# Supplementary material for: Age Distribution of All-Cause Mortality Among Children Younger Than 5 Years in Low- and Middle-Income Countries
Source: JAMA Netw Open. 2022 May 19;5(5):e2212692. doi: 10.1001/jamanetworkopen.2022.12692 (PMC9121187; doi:10.1001/jamanetworkopen.2022.12692)
Supplement: Supplement. — eMethods 1. Constructing Mortality Measures eMethods 2. Measure for Household Living Standards eTable 1. Information on Data eTable 2. Mortality and the Share of Deaths Among Children Younger Than 5 Years by Age: Period Restricted to 5 Years Before Survey Instead of 10 Years eTable 3. Share of Deaths Among Children Younger Than 5 Years Occurring at Different Ages Using Detailed Age Intervals: Period Restricted to 5 Years Before Survey Instead of 10 Years eTable 4. Component Mortality Probabilities for Pooled Sample eTable 5. Mortality and the Share of Deaths Among Children Younger Than 5 Years by Age eTable 6. Share of Deaths Among Children Younger Than 5 Years Occurring at Different Ages Using Detailed Age Intervals eTable 7. Mortality (Deaths per 1000) at Different Ages by Living Standards eTable 8. Shares of Deaths Among Children Younger Than 5 Years Occurring at Different Ages by Living Standards eTable 9. Mortality (Deaths per 1000) at Different Ages by Sex eTable 10. Shares of Deaths Among Children Younger Than 5 Years Occurring at Different Ages by Sex eFigure 1. Mortality Rate Among Children Younger Than 5 Years and Share of These Deaths Occurring at Different Ages: Pooled, Least Developed Countries, Regions, and World Bank Income Groups: Period Restricted to 5 Years Before Survey Instead of 10 Years eFigure 2. Share of Deaths Among Children Younger Than 5 Years Occurring at Different Ages Using Detailed Age Intervals: Pooled, Least Developed Countries, Regions, and World Bank Income Groups: Period Restricted to 5 Years Before Survey Instead of 10 Years eFigure 3. Mortality Rate Among Children Younger Than 5 Years and Share of These Deaths Occurring at Different Ages by Country: Period Restricted to 5 Years Before Survey Instead of 10 Years eFigure 4. Share of Deaths Among Children Younger Than 5 Years Occurring at Different Ages Using Detailed Age Intervals by Countries: Period Restricted to 5 Years Before Survey Instead of 10 Years eFigure 5. Lexis Diagram [file jamanetwopen-e2212692-s001.pdf]

Supplementary Online Content

Karlsson O, Kim R, Hasman A, Subramanian SV. Age distribution of all-cause mortality among children younger than 5 years in low- and middle-income countries. *JAMA Netw Open*. 2022;5(5):e2212692. doi:10.1001/jamanetworkopen.2022.12692

eMethods 1. Constructing Mortality Measures
eMethods 2. Measure for Household Living Standards
eTable 1. Information on Data
eTable 2. Mortality and the Share of Deaths Among Children Younger Than 5 Years by Age: Period Restricted to 5 Years Before Survey Instead of 10 Years
eTable 3. Share of Deaths Among Children Younger Than 5 Years Occurring at Different Ages Using Detailed Age Intervals: Period Restricted to 5 Years Before Survey Instead of 10 Years
eTable 4. Component Mortality Probabilities for Pooled Sample
eTable 5. Mortality and the Share of Deaths Among Children Younger Than 5 Years by Age
eTable 6. Share of Deaths Among Children Younger Than 5 Years Occurring at Different Ages Using Detailed Age Intervals
eTable 7. Mortality (Deaths per 1000) at Different Ages by Living Standards
eTable 8. Shares of Deaths Among Children Younger Than 5 Years Occurring at Different Ages by Living Standards
eTable 9. Mortality (Deaths per 1000) at Different Ages by Sex
eTable 10. Shares of Deaths Among Children Younger Than 5 Years Occurring at Different Ages by Sex
eFigure 1. Mortality Rate Among Children Younger Than 5 Years and Share of These Deaths Occurring at Different Ages: Pooled, Least Developed Countries, Regions, and World Bank Income Groups: Period Restricted to 5 Years Before Survey Instead of 10 Years
eFigure 2. Share of Deaths Among Children Younger Than 5 Years Occurring at Different Ages Using Detailed Age Intervals: Pooled, Least Developed Countries, Regions, and World Bank Income Groups: Period Restricted to 5 Years Before Survey Instead of 10 Years
eFigure 3. Mortality Rate Among Children Younger Than 5 Years and Share of These Deaths Occurring at Different Ages by Country: Period Restricted to 5 Years Before Survey Instead of 10 Years
eFigure 4. Share of Deaths Among Children Younger Than 5 Years Occurring at Different Ages Using Detailed Age Intervals by Countries: Period Restricted to 5 Years Before Survey Instead of 10 Years
eFigure 5. Lexis Diagram Demonstrating Inclusion for Each Component Mortality Probability

© 2022 Karlsson O et al. *JAMA Network Open*.

eFigure 6. Country-Level Association Between Mortality Rate Among Children Younger Than 5 Years and Share of These Deaths Occurring at Different Ages
eFigure 7. Share of Deaths Among Children Younger Than 5 Years Occurring at Different Ages Using Detailed Age Intervals by Countries
eFigure 8. Share of Deaths Among Children Younger Than 5 Years Occurring at Different Ages: Countries by Living Standards
eFigure 9. Share of Deaths Among Children Younger Than 5 Years Occurring at Different Ages: Countries by Sex
eReferences

This supplementary material has been provided by the authors to give readers additional information about their work.

© 2022 Karlsson O et al. *JAMA Network Open*.

eMethods 1. Constructing Mortality Measures

In the surveys, age at death was usually recorded in days, months, or years for children that died at 24 months or older. Therefore, we used age at death in months, as imputed in the DHS and MICS, where ages reported in days were converted to months, ages reported in months were used directly, and ages reported in years were truncated to months (ie, three years as 36 months). Missing age at death was imputed using a quasi-random "hot deck" method, based on the age at death for the closest preceding observation in the data with the same birth interval and, if available, the same form of reporting for age at death (ie, days, months, or years). Date of birth was randomly imputed after imposing certain logical ranges (eg, based on other dates, birth intervals, and maternal age at birth, duration of amenorrhea, and abstinence) and constraints (eg, age or age at death) [1].

We used a direct method for calculating mortality rates. Direct methods are preferable to indirect methods, which only use information on births and deaths for cohorts of mothers as opposed to data on date of birth and age at death of deceased children. Indirect methods rely on a broader set of assumptions than direct methods, such as stable fertility levels and patterns, no change or linear change in mortality, and a pattern of mortality derived from other populations, often European. We followed the United Nations Population Division's recommendation for calculation and presentation of mortality rates to facilitate comparison to other sources [2]. Mortality rates were estimated using the same approach used in reports from the DHS and MICS: a synthetic cohort life table approach [3–6]. (Note that under-5 mortality rates in the DHS reports may differ since we restricted the period to ten years before a survey while the DHS usually limits it to five years for national-level estimates.)

A synthetic cohort life table was constructed and component mortality probabilities for small age intervals estimated, which were then combined to calculate a mortality probability for the age group of interest. (As such, it is similar to a product limit estimator.) This approach differs from a vital statistics approach, where deaths over a period are divided by births over the same period, in that the vital statistics approach produces true rates, rather than probabilities, and the variation in the number of births over time is not accounted for. The synthetic cohort life table approach also differs from a true life table approach, where the number of deaths in a specific birth cohort is divided by the number of deaths in that specific birth cohort, so all children must have lived through the risk period, which in this study would mean that only children born more than five years before each survey would be included. The synthetic cohort life table approach allows the use of most recent births and is also specific to a period (ie, the ten years preceding the survey in our study).

First, component mortality probabilities were constructed for the age intervals less than one month, 1–2 months, 3–5 months, 6–11 months, 12–23 months, 24–35 months, 36–47 months, and 48–59 months. (Note that here we use *age groups* to refer to the broader age groups which are of primary interest—under-5, under-2, and neonatal—and *age interval* to refer to the age segments used to calculate the component mortality probabilities, which are then used for estimating mortality rates for the broader age groups.) eFigure 5 shows a Lexis diagram which illustrates the three cohorts of children included in the component mortality probability for a specific age interval and period: the y-axis shows an age interval for a specific component mortality probability which ranges from a_1 to a_2 (0 to < 1 month, 1–2 months, 3–5 months, etc.) and the x-axis shows the period for which the component mortality probability is being calculated, ranging from t_1 (120 months before the survey in our study) to t_2 (one month before the survey). t_1 and t_2 can vary, but in this study, we use a single period. The component mortality probability for the specific age interval includes three cohorts of children: B, who are exposed to the entire period, reaching the (hypothetical) ages a_1 and a_2 within the period t_1 to t_2 (ie, born between t_1-a_1 and t_2-a_2); A who reach age a_1 before the period starts at t_1 and age a_2 before the period ends at t_2 (ie, born between t_1-a_2 and t_1-a_2); and C who reach age a_1 after the period starts at t_1 but do not reach age a_2 before the period ends at t_2 (ie, born between t_2-a_2 and t_2-a_1).

It was assumed that half of the exposures occurred in the relevant age interval for only partially exposed children in cohorts A and C and half the deaths for partially exposed children in cohort A. Note that all deaths of children in the relevant age interval for children in cohort C were included in this study since the end of the period t_2 was the date of the interview and those deaths must have occurred before the interview. The events occurring in the month of the interview were excluded since they did not contribute an entire month of deaths and exposures. The component probability q_i for each age interval i was then calculated as:

$$q_i = \frac{0.5 \times Ad_i + Bd_i + Cd_i}{0.5 \times Ac_i + Bc_i + 0.5 \times Cc_i} \qquad \text{Eq.1}$$

© 2022 Karlsson O et al. *JAMA Network Open*.

or half the deaths of children in cohort A (Ad) and all deaths in cohorts B (Bd) and C (Cd) occurring in age interval i divided by half the cases in cohorts A (Ac) and C (Cc) and all cases in cohort B (Bc) exposed to age interval i. These component mortality probabilities were converted to survival probabilities by subtracting them from one $(1 - q_i)$.

$$ {}_nq_x = 1 - \prod_{i=n}^{i=x+n}(1 - q_i) \qquad \text{Eq.2} $$

The mortality rates (${}_nq_x \times 1000$) for the age groups (n to x)—ages 0 to < 1 month, 0 to 23, and 0 to 59—were calculated by subtracting the product of the component probabilities across the relevant age group from one [6].

eTable 4 demonstrates the synthetic cohort life table for our pooled sample. Each component (mortality) probability in the second last column was obtained by dividing half the deaths of children in cohort A and all deaths in cohorts B and C by half the cases in cohorts A and C and all cases in cohort B (as in Eq.1). The under-5 mortality rate was obtained by multiplying together all rows in the last column and further multiplying by 1000 (as in Eq.2). The under-2 mortality rate was obtained by multiplying the first five rows of the last column together and further multiplying by 1000. The neonatal mortality rate was $q_0 \times 1000$ for the first age interval shown in the first row second last column.

Mortality for children 1–23 months old was calculated by subtracting the neonatal mortality rate from mortality rate of children aged 0–23 months old (as neonatal mortality rate is subtracted from the infant mortality rate to construct postneonatal mortality rate in the reports from the DHS [5,6]). Similarly, mortality for children 24–59 months old was calculated by subtracting the mortality rate for children 0–23 months old from the under-5 mortality rate. Therefore, the mortality measures for neonates, children 1–23 months old, and children 24–59 months old, sum up to the under-5 mortality rate. The share measures were then calculated by dividing the mortality measures for mortality at 0–23 months, <1 month, 1–23 months, and 24–59 months by the under-5 mortality rate and multiplying by 100 to obtain percentages of under-5 deaths occurring in each age group.

© 2022 Karlsson O et al. *JAMA Network Open*.

eMethods 2. Measure for Household Living Standards

A household wealth index was constructed (by the DHS and MICS) using information on the household's ownership of assets (eg, car, television, refrigerator) and amenities (eg, type of toilet facility, electricity access, source of water) using principal component analysis [7]. Households were divided into five equally sized groups, each containing 20% of households (quintiles), based on the household wealth index. We show results comparing the top quintile (20% of households with the best living standards) to the bottom quintile (20% of households with the worst living standards) within each country.

© 2022 Karlsson O et al. *JAMA Network Open*.

eTable 1. Information on Data

Country	Survey year	Observations	Number of births ('000)	LDC	UNICEF-Region	Income group	Data
Pooled		2,827,515	878,562				
Afghanistan	2015	90,971	11,727	Yes	South Asia	LIC	DHS
Albania	2017-2018	9,131	357		Europe & Central Asia	UMIC	DHS
Angola	2015-2016	32,544	10,666	Yes	Eastern & Southern Africa	LMIC	DHS
Armenia	2015-2016	5,103	440		Europe & Central Asia	UMIC	DHS
Bangladesh	2019	73,146	30,241	Yes	South Asia	LMIC	MICS
Benin	2017-2018	33,565	3,758	Yes	West & Central Africa	LMIC	DHS
Burkina Faso	2010	39,950	6,028	Yes	West & Central Africa	LIC	DHS
Burundi	2016-2017	34,295	3,804	Yes	Eastern & Southern Africa	LIC	DHS
Cambodia	2014	20,962	3,610	Yes	East Asia & Pacific	LMIC	DHS
Cameroon	2018-2019	24,613	8,372		West & Central Africa	LMIC	DHS
Chad	2014-2015	51,289	5,463	Yes	West & Central Africa	LIC	DHS
Colombia	2015-2016	36,494	7,637		Latin America & Caribbean	UMIC	DHS
Comoros	2012	8,281	231	Yes	Eastern & Southern Africa	LMIC	DHS
Congo	2011-2012	22,680	1,432		West & Central Africa	LMIC	DHS
Congo (DR)	2013-2014	44,608	27,504	Yes	West & Central Africa	LIC	DHS
Cote d'Ivoire	2016	23,660	7,889		West & Central Africa	LMIC	MICS
Dominican Rep.	2014	40,182	2,077		Latin America & Caribbean	UMIC	MICS
Egypt	2014	40,620	21,948		Middle East & North Africa	LMIC	DHS
El Salvador	2014	15,865	1,239		Latin America & Caribbean	LMIC	MICS
Ethiopia	2016	29,854	32,116	Yes	Eastern & Southern Africa	LIC	DHS
Gabon	2012	15,291	496		West & Central Africa	UMIC	DHS
Gambia	2018	26,996	791	Yes	West & Central Africa	LIC	MICS
Ghana	2014	15,832	7,888		West & Central Africa	LMIC	DHS
Guatemala	2014-2015	35,185	4,084		Latin America & Caribbean	UMIC	DHS
Guinea	2018	20,966	4,224	Yes	West & Central Africa	LIC	DHS
Guyana	2014	7,424	162		Latin America & Caribbean	UMIC	MICS
Haiti	2016-2017	18,293	2,743	Yes	Latin America & Caribbean	LIC	DHS
Honduras	2011-2012	31,415	2,153		Latin America & Caribbean	LMIC	DHS
India	2015-2016	798,690	260,232		South Asia	LMIC	DHS
Indonesia	2017	53,671	49,778		East Asia & Pacific	UMIC	DHS
Iraq	2018	51,289	10,582		Middle East & North Africa	UMIC	MICS
Jordan	2017-2018	31,781	2,118		Middle East & North Africa	UMIC	DHS
Kenya	2014	59,861	14,551		Eastern & Southern Africa	LMIC	DHS
Kyrgyz Rep.	2018	8,783	1,529		Europe & Central Asia	LMIC	MICS
Lao	2017	34,275	1,672	Yes	East Asia & Pacific	LMIC	MICS
Lesotho	2018	7,583	577	Yes	Eastern & Southern Africa	LMIC	MICS
Liberia	2013	20,692	1,386	Yes	West & Central Africa	LIC	DHS
Madagascar	2018	32,998	7,871	Yes	Eastern & Southern Africa	LIC	MICS
Malawi	2015-2016	48,309	5,836	Yes	Eastern & Southern Africa	LIC	DHS
Maldives	2016-2017	8,543	72		South Asia	UMIC	DHS
Mali	2018	25,363	7,325	Yes	West & Central Africa	LIC	DHS
Mauritania	2015	28,187	1,279	Yes	West & Central Africa	LMIC	MICS
Moldova	2012	4,414	443		Europe & Central Asia	LMIC	MICS
Mongolia	2018	15,381	721		East Asia & Pacific	LMIC	MICS
Mozambique	2011	27,381	9,000	Yes	Eastern & Southern Africa	LIC	DHS
Myanmar	2015-2016	14,719	10,090	Yes	East Asia & Pacific	LMIC	DHS
Namibia	2013	12,741	617		Eastern & Southern Africa	UMIC	DHS
Nepal	2019	17,862	5,823	Yes	South Asia	LMIC	MICS
Niger	2012	33,401	7,572	Yes	West & Central Africa	LIC	DHS
Nigeria	2018	89,319	69,010		West & Central Africa	LMIC	DHS
Pakistan	2017-2018	35,704	55,669		South Asia	LMIC	DHS
Papua New Guinea	2016-2018	25,363	2,205		East Asia & Pacific	LMIC	DHS
Paraguay	2016	9,782	1,410		Latin America & Caribbean	UMIC	MICS
Peru	2012	28,366	6,092		Latin America & Caribbean	UMIC	DHS
Philippines	2017	31,648	23,164		East Asia & Pacific	LMIC	DHS
Rwanda	2014-2015	21,899	3,443	Yes	Eastern & Southern Africa	LIC	DHS
Sao Tome & Principe	2014	5,304	64	Yes	West & Central Africa	LMIC	MICS
Senegal	2019	16,357	5,200	Yes	West & Central Africa	LMIC	DHS
Sierra Leone	2019	27,532	2,510	Yes	West & Central Africa	LIC	DHS
South Africa	2016	9,534	11,886		Eastern & Southern Africa	UMIC	DHS
South Sudan	2010	24,384	3,104	Yes	Eastern & Southern Africa	LIC	MICS
State of Palestine	2014	21,342	1,335		Middle East & North Africa	LMIC	MICS
Sudan	2014	40,049	12,168	Yes	Eastern & Southern Africa	LIC	MICS
Suriname	2018	9,537	108		Latin America & Caribbean	UMIC	MICS
Swaziland	2014	6,476	324		Eastern & Southern Africa	LMIC	MICS
Tajikistan	2017	15,078	2,507		Europe & Central Asia	LIC	DHS
Tanzania	2015-2016	25,929	17,879	Yes	Eastern & Southern Africa	LMIC	DHS
Timor-Leste	2016	20,945	343	Yes	East Asia & Pacific	LMIC	DHS
Togo	2017	13,383	2,444	Yes	West & Central Africa	LIC	MICS
Tonga	2019	3,899	26		East Asia & Pacific	UMIC	MICS
Tunisia	2018	10,215	2,002		Middle East & North Africa	LMIC	MICS
Turkey	2013	10,957	13,146		Europe & Central Asia	UMIC	DHS
Turkmenistan	2019	9,964	1,390		Europe & Central Asia	UMIC	MICS
Uganda	2016	40,628	14,731	Yes	Eastern & Southern Africa	LIC	DHS
Yemen	2013	45,923	7,748	Yes	Middle East & North Africa	LIC	DHS
Zambia	2018-2019	27,019	5,879	Yes	Eastern & Southern Africa	LMIC	DHS

© 2022 Karlsson O et al. *JAMA Network Open*.

Zimbabwe	2019	15,840	4,621		Eastern & Southern Africa	LMIC	MICS

Notes: Observations refer to children that were under five years old at any point during the 120 months before each survey (excluding children born in the month of survey). The Population Division of the United Nations provides data on the number of births for five-year periods: two five-year periods are summed and interpolated to a yearly series and then linked to the survey year in each country and used to rescale the sampling weights. Acronyms and abbreviations: Republic (Rep.), Democratic Republic (DR), Low-Income Countries (LIC), Lower-Middle-Income Countries (LMIC), Upper-Middle-Income Countries (UMIC)

© 2022 Karlsson O et al. *JAMA Network Open*.

eTable 2. Mortality and the Share of Deaths Among Children Younger Than 5 Years by Age: Period Restricted to 5 Years Before Survey Instead of 10 Years

	Mortality (deaths per 1000)					Share (%)			
	0–59 months	0–23 months	Neonates	1–23 months	24–59 months	0–23 months	Neonates	1–23 months	24–59 months
Pooled	62.6	50.6	27.1	23.5	12.0	80.9	43.3	37.6	19.1
	[61.3,63.9]	[49.6,51.7]	[26.4,27.8]	[22.8,24.2]	[11.4,12.5]	[80.3,81.5]	[42.4,44.2]	[36.8,38.5]	[18.5,19.7]
Least Developed Countries	73.2	57.3	26.9	30.4	15.9	78.3	36.8	41.5	21.7
	[71.3,75.2]	[55.8,59.0]	[26.0,27.9]	[29.3,31.6]	[15.2,16.6]	[77.6,79.1]	[35.7,37.9]	[40.6,42.5]	[20.9,22.4]
East Asia & Pacific	33.2	28.7	16.2	12.5	4.5	86.4	48.7	37.7	13.6
	[31.1,35.5]	[26.7,30.8]	[14.8,17.7]	[11.1,14.1]	[3.7,5.5]	[83.7,88.6]	[45.3,52.2]	[34.2,41.2]	[11.4,16.3]
Cambodia	34.7	30.4	17.8	12.6	4.3	87.6	51.3	36.4	12.4
	[28.1,42.9]	[24.3,38.1]	[13.1,24.2]	[9.7,16.5]	[3.1,6.0]	[83.4,90.9]	[43.0,59.5]	[29.0,44.4]	[9.1,16.6]
Indonesia	31.6	27.5	15.5	12.0	4.2	86.8	48.9	37.9	13.2
	[28.8,34.7]	[25.0,30.1]	[13.9,17.2]	[10.2,14.1]	[3.0,5.9]	[82.2,90.4]	[44.4,53.4]	[33.5,42.6]	[9.6,17.8]
Lao	45.5	41.5	17.8	23.6	4.1	91.0	39.1	51.9	9.0
	[40.3,51.4]	[36.4,47.2]	[14.9,21.3]	[20.1,27.7]	[3.0,5.5]	[88.0,93.3]	[34.2,44.3]	[46.7,57.0]	[6.7,12.0]
Mongolia	19.3	16.3	8.7	7.6	3.0	84.6	45.2	39.3	15.4
	[14.6,25.4]	[11.9,22.4]	[5.8,13.1]	[5.0,11.6]	[1.6,5.6]	[72.6,91.9]	[33.0,58.1]	[28.7,51.2]	[8.1,27.4]
Myanmar	50.0	43.3	24.5	18.8	6.7	86.6	49.1	37.5	13.4
	[42.5,58.7]	[36.3,51.5]	[19.7,30.6]	[13.4,26.3]	[4.3,10.3]	[80.1,91.3]	[39.2,59.1]	[28.9,47.1]	[8.7,19.9]
Papua New Guinea	48.5	37.6	20.4	17.2	10.9	77.5	42.0	35.5	22.5
	[43.8,53.8]	[33.4,42.4]	[17.1,24.3]	[13.9,21.4]	[8.3,14.4]	[71.6,82.5]	[35.3,49.1]	[29.6,41.9]	[17.5,28.4]
Philippines	27.3	23.5	13.6	9.8	3.8	86.0	49.9	36.0	14.0
	[23.3,32.0]	[19.8,27.8]	[10.6,17.5]	[7.4,13.0]	[2.6,5.6]	[80.5,90.0]	[40.8,59.1]	[28.2,44.7]	[10.0,19.5]
Timor-Leste	41.4	33.0	18.5	14.5	8.4	79.7	44.7	34.9	20.3
	[36.0,47.5]	[28.9,37.6]	[15.3,22.4]	[11.5,18.2]	[5.9,12.0]	[73.3,84.9]	[37.7,52.0]	[28.4,42.1]	[15.1,26.7]
Tonga	16.4	15.0	7.1	7.8	1.5	91.1	43.3	47.8	8.9
	[9.7,27.7]	[8.4,26.4]	[4.3,11.9]	[3.3,18.7]	[0.4,5.3]	[68.2,98.0]	[25.8,62.7]	[25.3,71.1]	[2.0,31.8]
South Asia	52.2	46.8	30.4	16.4	5.4	89.7	58.3	31.4	10.3
	[50.6,53.9]	[45.2,48.5]	[29.2,31.8]	[15.6,17.1]	[5.1,5.7]	[89.0,90.3]	[56.9,59.7]	[30.3,32.5]	[9.7,11.0]
Afghanistan	55.2	49.4	22.2	27.2	5.8	89.5	40.2	49.3	10.5
	[49.4,61.5]	[43.9,55.4]	[18.8,26.2]	[23.6,31.3]	[4.8,7.0]	[87.4,91.3]	[35.9,44.7]	[44.7,53.9]	[8.7,12.6]
Bangladesh	39.6	35.6	25.9	9.6	4.0	89.9	65.5	24.4	10.1
	[37.0,42.3]	[33.0,38.3]	[23.9,28.1]	[8.2,11.4]	[3.2,5.1]	[87.2,92.1]	[61.6,69.3]	[21.2,27.9]	[7.9,12.8]
India	49.7	44.3	29.5	14.8	5.4	89.1	59.2	29.8	10.9
	[48.7,50.8]	[43.4,45.2]	[28.5,30.4]	[14.2,15.5]	[5.1,5.8]	[88.4,89.7]	[57.9,60.6]	[28.7,31.0]	[10.3,11.6]
Maldives	20.4	18.6	11.4	7.2	1.8	91.0	55.8	35.2	9.0
	[14.0,29.7]	[12.4,27.7]	[6.8,19.0]	[3.3,15.7]	[0.9,3.7]	[82.7,95.5]	[34.2,75.4]	[17.6,58.0]	[4.5,17.3]
Nepal	28.4	25.5	15.9	9.6	3.0	89.6	55.9	33.8	10.4
	[22.7,35.5]	[20.0,32.4]	[12.1,20.9]	[6.9,13.3]	[1.9,4.5]	[84.1,93.4]	[48.2,63.3]	[26.5,41.9]	[6.6,15.9]
Pakistan	70.7	64.7	39.9	24.7	6.0	91.5	56.5	35.0	8.5
	[63.2,79.0]	[57.7,72.4]	[34.7,46.0]	[20.6,29.7]	[4.5,8.1]	[88.8,93.6]	[51.3,61.5]	[30.2,40.1]	[6.4,11.2]
Europe & Central Asia	19.5	17.8	9.8	8.0	1.7	91.5	50.5	41.1	8.5
	[16.1,23.5]	[14.7,21.6]	[7.7,12.5]	[6.3,10.2]	[1.1,2.5]	[87.4,94.4]	[43.7,57.2]	[34.5,47.9]	[5.6,12.6]
Albania	4.5	4.5	1.7	2.8	NA	NA	37.3	62.7	NA
	[2.0,10.1]	[2.0,10.1]	[0.5,6.3]	[0.9,8.9]			[5.6,85.6]	[14.4,94.4]	
Armenia	6.3	5.3	3.1	2.2	1.1	83.3	48.5	34.7	16.7
	[3.8,10.4]	[3.0,9.2]	[1.5,6.1]	[0.8,5.9]	[0.2,4.8]	[47.6,96.5]	[23.1,74.7]	[14.5,62.6]	[3.5,52.4]
Kyrgyz Republic	19.6	17.3	13.4	3.8	2.3	88.1	68.5	19.6	11.9
	[14.0,27.5]	[11.7,25.5]	[8.9,20.3]	[2.0,7.4]	[1.1,4.7]	[75.0,94.8]	[55.0,79.5]	[10.8,33.0]	[5.2,25.0]
Moldova	16.3	14.6	13.3	1.4	1.6	89.9	81.6	8.3	10.1
	[10.1,26.2]	[9.2,23.2]	[8.1,21.7]	[0.3,5.9]	[0.4,6.3]	[67.4,97.5]	[58.4,93.3]	[1.2,40.7]	[2.5,32.6]
Tajikistan	33.4	29.9	12.8	17.1	3.5	89.5	38.4	51.1	10.5

© 2022 Karlsson O et al. *JAMA Network Open*.

	[29.0,38.4]	[25.9,34.4]	[10.0,16.4]	[13.7,21.2]	[2.1,5.9]	[83.3,93.6]	[30.0,47.5]	[42.8,59.4]	[6.4,16.7]
Turkey	14.9	14.0	7.4	6.6	0.9	93.9	49.7	44.2	6.1
	[10.2,21.8]	[9.6,20.4]	[5.0,11.1]	[3.4,12.6]	[0.4,2.3]	[86.4,97.4]	[32.1,67.4]	[27.8,62.1]	[2.6,13.6]
Turkmenistan	37.9	33.4	22.1	11.3	4.4	88.3	58.4	29.9	11.7
	[31.2,45.8]	[27.3,40.8]	[16.9,28.9]	[7.9,16.3]	[2.6,7.5]	[81.5,92.7]	[48.0,68.0]	[21.0,40.7]	[7.3,18.5]
Middle East & North Africa	30.2	27.3	15.5	11.8	2.9	90.3	51.3	39.1	9.7
	[28.1,32.5]	[25.2,29.6]	[13.9,17.2]	[10.7,13.0]	[2.4,3.5]	[88.3,92.0]	[48.3,54.2]	[36.1,42.1]	[8.0,11.7]
Egypt	27.5	24.4	13.8	10.6	3.1	88.7	50.1	38.6	11.3
	[24.8,30.5]	[21.8,27.2]	[11.9,16.0]	[8.9,12.6]	[2.2,4.3]	[84.6,91.9]	[44.8,55.4]	[33.6,43.9]	[8.1,15.4]
Iraq	25.7	23.4	14.4	9.0	2.3	90.9	56.1	34.8	9.1
	[21.8,30.3]	[19.8,27.7]	[11.4,18.3]	[7.3,10.9]	[1.5,3.6]	[86.9,93.8]	[49.8,62.2]	[28.4,41.9]	[6.2,13.1]
Jordan	19.4	17.6	10.7	6.9	1.8	90.8	55.5	35.4	9.2
	[15.6,24.1]	[14.1,21.9]	[8.0,14.4]	[4.7,10.1]	[0.9,3.3]	[83.9,95.0]	[42.3,67.9]	[25.0,47.3]	[5.0,16.1]
State of Palestine	21.7	20.0	11.2	8.9	1.7	92.3	51.4	40.8	7.7
	[18.3,25.8]	[16.5,24.3]	[8.9,14.0]	[6.7,11.7]	[0.9,3.0]	[85.9,95.9]	[44.1,58.7]	[33.1,49.0]	[4.1,14.1]
Tunisia	16.7	15.0	8.7	6.3	1.7	89.6	51.8	37.9	10.4
	[13.0,21.5]	[11.3,19.8]	[6.0,12.4]	[4.0,10.1]	[0.9,3.3]	[80.3,94.8]	[37.0,66.3]	[26.0,51.4]	[5.2,19.7]
Yemen	52.8	48.6	25.9	22.7	4.2	92.1	49.1	43.1	7.9
	[47.3,58.8]	[43.7,54.0]	[22.4,29.9]	[19.6,26.3]	[2.9,5.9]	[89.3,94.3]	[44.0,54.2]	[38.4,47.8]	[5.7,10.7]
Eastern & Southern Africa	65.5	53.8	26.7	27.1	11.6	82.3	40.8	41.4	17.7
	[63.1,67.9]	[51.9,55.8]	[25.3,28.2]	[25.6,28.7]	[10.7,12.7]	[80.9,83.5]	[38.9,42.8]	[39.8,43.1]	[16.5,19.1]
Angola	68.3	52.5	24.2	28.3	15.8	76.9	35.4	41.5	23.1
	[60.1,77.5]	[45.9,60.1]	[20.2,28.9]	[24.2,33.1]	[13.0,19.2]	[73.2,80.2]	[31.2,39.8]	[37.7,45.4]	[19.8,26.8]
Burundi	78.1	59.2	22.9	36.2	19.0	75.7	29.4	46.4	24.3
	[72.3,84.4]	[53.5,65.5]	[19.3,27.2]	[32.0,41.0]	[16.9,21.2]	[72.6,78.7]	[25.5,33.6]	[42.3,50.5]	[21.3,27.4]
Comoros	49.7	38.6	23.5	15.0	11.1	77.6	47.3	30.2	22.4
	[40.7,60.6]	[30.3,49.0]	[18.0,30.8]	[10.3,21.9]	[7.2,17.1]	[67.2,85.4]	[39.1,55.7]	[21.7,40.4]	[14.6,32.8]
Ethiopia	67.0	55.5	29.5	26.1	11.5	82.9	44.0	38.9	17.1
	[59.9,75.0]	[49.9,61.8]	[24.4,35.6]	[21.6,31.5]	[8.7,15.2]	[78.6,86.4]	[37.0,51.2]	[32.6,45.6]	[13.6,21.4]
Kenya	52.4	44.3	22.3	22.0	8.0	84.7	42.6	42.1	15.3
	[48.0,57.1]	[40.0,49.1]	[19.6,25.4]	[18.8,25.8]	[6.4,10.0]	[81.0,87.8]	[38.1,47.2]	[37.3,47.0]	[12.2,19.0]
Lesotho	76.2	66.1	36.4	29.7	10.1	86.7	47.8	38.9	13.3
	[62.0,93.3]	[54.1,80.4]	[28.2,46.9]	[23.0,38.2]	[5.6,18.3]	[78.5,92.1]	[39.8,55.9]	[32.1,46.2]	[7.9,21.5]
Madagascar	58.7	43.7	21.0	22.6	15.0	74.5	35.9	38.6	25.5
	[54.0,63.8]	[39.8,48.0]	[18.6,23.9]	[19.3,26.6]	[13.0,17.2]	[71.5,77.2]	[31.7,40.2]	[34.3,43.1]	[22.8,28.5]
Malawi	63.8	49.1	26.7	22.4	14.7	76.9	41.8	35.1	23.1
	[58.6,69.4]	[44.5,54.1]	[23.2,30.6]	[19.6,25.6]	[11.9,18.2]	[72.2,81.0]	[37.1,46.6]	[31.4,39.0]	[19.0,27.8]
Mozambique	96.9	78.0	30.4	47.6	18.9	80.5	31.4	49.1	19.5
	[89.9,104.4]	[72.1,84.4]	[26.2,35.3]	[43.1,52.6]	[15.5,23.0]	[77.1,83.6]	[27.6,35.5]	[45.2,53.0]	[16.4,22.9]
Namibia	54.5	45.9	19.7	26.2	8.6	84.2	36.2	48.0	15.8
	[46.2,64.1]	[38.2,55.0]	[14.6,26.6]	[21.6,31.7]	[6.2,11.9]	[78.6,88.6]	[28.9,44.2]	[41.1,55.0]	[11.4,21.4]
Rwanda	50.4	39.6	19.6	20.0	10.8	78.6	38.9	39.7	21.4
	[45.3,56.0]	[35.2,44.4]	[16.7,23.0]	[16.9,23.7]	[8.9,13.0]	[75.0,81.8]	[34.0,44.0]	[35.1,44.5]	[18.2,25.0]
South Africa	42.1	38.2	21.0	17.2	3.9	90.8	50.0	40.8	9.2
	[34.1,51.8]	[30.3,48.0]	[14.8,29.8]	[13.1,22.5]	[2.6,5.9]	[85.9,94.1]	[39.6,60.4]	[31.8,50.4]	[5.9,14.1]
South Sudan	108.3	90.5	42.6	47.9	17.7	83.6	39.4	44.3	16.4
	[98.8,118.5]	[81.7,100.2]	[35.9,50.5]	[42.4,54.1]	[14.5,21.6]	[80.3,86.5]	[34.5,44.5]	[40.0,48.6]	[13.5,19.7]
Sudan	68.4	60.1	32.6	27.5	8.3	87.9	47.6	40.3	12.1
	[63.2,74.0]	[55.3,65.3]	[29.1,36.4]	[24.2,31.3]	[6.9,10.0]	[85.6,89.8]	[43.4,51.9]	[36.5,44.1]	[10.2,14.4]
Swaziland	67.0	57.4	20.1	37.3	9.6	85.7	30.0	55.7	14.3
	[55.1,81.2]	[45.3,72.6]	[14.0,28.9]	[28.7,48.4]	[5.7,15.9]	[76.3,91.8]	[22.5,38.9]	[46.9,64.1]	[8.2,23.7]
Tanzania	67.2	55.9	25.5	30.4	11.3	83.2	37.9	45.3	16.8
	[58.6,76.8]	[49.0,63.7]	[21.4,30.3]	[25.5,36.2]	[8.6,14.8]	[79.5,86.4]	[32.8,43.2]	[40.6,50.1]	[13.6,20.5]

© 2022 Karlsson O et al. *JAMA Network Open*.

Uganda	64.3	52.1	26.7	25.3	12.2	81.0	41.6	39.4	19.0
	[59.9,69.0]	[48.4,56.0]	[24.7,29.0]	[22.6,28.4]	[10.4,14.3]	[78.5,83.3]	[39.0,44.2]	[36.1,42.8]	[16.7,21.5]
Zambia	60.5	49.5	27.4	22.1	11.0	81.9	45.4	36.5	18.1
	[55.1,66.4]	[44.8,54.7]	[23.8,31.7]	[19.3,25.3]	[8.8,13.7]	[78.0,85.2]	[40.4,50.5]	[32.8,40.5]	[14.8,22.0]
Zimbabwe	64.9	55.3	32.3	23.0	9.6	85.2	49.8	35.4	14.8
	[56.7,74.2]	[48.7,62.7]	[26.6,39.1]	[19.8,26.7]	[6.9,13.3]	[81.1,88.5]	[44.3,55.3]	[30.1,41.1]	[11.5,18.9]
West & Central Africa	111.4	78.0	32.9	45.1	33.4	70.0	29.5	40.5	30.0
	[108.1,114.7]	[75.3,80.7]	[31.1,34.7]	[43.4,47.0]	[32.0,34.8]	[69.0,71.0]	[28.4,30.7]	[39.3,41.7]	[29.0,31.0]
Benin	96.2	68.3	30.0	38.2	28.0	70.9	31.2	39.7	29.1
	[89.2,103.7]	[62.5,74.5]	[26.2,34.3]	[34.7,42.1]	[24.6,31.7]	[67.8,73.9]	[28.2,34.4]	[36.8,42.8]	[26.1,32.2]
Burkina Faso	128.5	89.3	28.1	61.3	39.2	69.5	21.8	47.7	30.5
	[121.0,136.5]	[83.3,95.7]	[25.4,31.0]	[56.1,66.8]	[35.9,42.8]	[67.4,71.5]	[20.0,23.8]	[45.1,50.2]	[28.5,32.6]
Cameroon	79.6	59.4	28.1	31.3	20.2	74.6	35.3	39.3	25.4
	[72.9,86.9]	[53.6,65.8]	[24.3,32.5]	[27.1,36.2]	[17.3,23.6]	[71.1,77.9]	[31.3,39.5]	[35.3,43.5]	[22.1,28.9]
Chad	133.0	95.0	33.8	61.2	38.0	71.4	25.4	46.0	28.6
	[124.1,142.4]	[86.0,104.9]	[29.6,38.6]	[54.9,68.2]	[35.0,41.2]	[68.5,74.2]	[23.0,28.0]	[43.3,48.8]	[25.8,31.5]
Congo	67.8	50.4	21.5	28.8	17.4	74.3	31.8	42.5	25.7
	[60.1,76.4]	[43.4,58.4]	[16.8,27.6]	[24.2,34.3]	[14.5,20.8]	[69.7,78.4]	[25.9,38.3]	[37.3,48.0]	[21.6,30.3]
Congo (DR)	104.3	74.1	28.0	46.1	30.2	71.0	26.8	44.2	29.0
	[97.2,111.8]	[68.4,80.2]	[24.4,32.0]	[41.8,50.9]	[26.5,34.4]	[67.9,74.0]	[23.9,30.0]	[41.0,47.5]	[26.0,32.1]
Cote D'Ivoire	96.2	74.3	33.3	41.0	21.9	77.2	34.6	42.6	22.8
	[87.5,105.8]	[67.2,82.1]	[29.5,37.7]	[35.2,47.6]	[18.1,26.5]	[73.6,80.5]	[31.0,38.4]	[38.2,47.1]	[19.5,26.4]
Gabon	64.6	49.7	26.3	23.4	14.9	76.9	40.7	36.2	23.1
	[56.6,73.7]	[41.7,59.3]	[20.0,34.6]	[18.5,29.5]	[10.6,20.9]	[68.4,83.7]	[32.3,49.7]	[29.2,43.9]	[16.3,31.6]
Gambia	57.2	46.9	30.6	16.2	10.4	81.9	53.5	28.3	18.1
	[51.1,64.0]	[41.6,52.7]	[25.6,36.6]	[13.5,19.5]	[8.3,13.0]	[78.2,85.1]	[47.0,59.9]	[23.5,33.8]	[14.9,21.8]
Ghana	59.9	46.7	28.7	18.0	13.2	78.0	47.9	30.1	22.0
	[51.0,70.1]	[39.4,55.3]	[22.6,36.3]	[14.2,22.9]	[9.1,19.0]	[70.3,84.2]	[41.4,54.5]	[23.2,38.0]	[15.8,29.7]
Guinea	111.5	82.9	32.3	50.6	28.6	74.4	29.0	45.4	25.6
	[100.6,123.3]	[74.3,92.4]	[28.2,36.9]	[43.2,59.2]	[24.3,33.6]	[71.2,77.3]	[25.4,32.9]	[41.2,49.7]	[22.7,28.8]
Liberia	93.8	73.2	26.2	47.0	20.6	78.0	28.0	50.1	22.0
	[84.8,103.8]	[64.7,82.7]	[21.7,31.7]	[40.7,54.2]	[17.7,24.0]	[74.4,81.2]	[24.0,32.3]	[45.6,54.5]	[18.8,25.6]
Mali	101.1	69.6	32.5	37.1	31.5	68.9	32.2	36.7	31.1
	[92.8,110.0]	[63.4,76.3]	[28.0,37.7]	[32.9,41.7]	[27.4,36.1]	[65.6,71.9]	[28.3,36.3]	[33.4,40.0]	[28.1,34.4]
Mauritania	53.8	45.6	28.9	16.7	8.2	84.8	53.7	31.1	15.2
	[47.1,61.3]	[39.9,52.1]	[24.8,33.6]	[13.7,20.4]	[6.3,10.6]	[81.0,88.0]	[48.9,58.5]	[26.8,35.8]	[12.0,19.0]
Niger	127.3	76.5	24.2	52.4	50.8	60.1	19.0	41.1	39.9
	[119.4,135.7]	[70.3,83.3]	[21.1,27.7]	[47.1,58.2]	[46.3,55.7]	[57.1,63.0]	[16.7,21.5]	[38.1,44.3]	[37.0,42.9]
Nigeria	132.0	90.4	39.3	51.1	41.7	68.4	29.7	38.7	31.6
	[123.7,140.8]	[84.6,96.5]	[35.2,43.8]	[47.3,55.3]	[38.0,45.6]	[66.7,70.1]	[27.3,32.3]	[36.5,41.0]	[29.9,33.3]
Sao Tome & Principe	44.9	41.0	21.9	19.1	3.9	91.4	48.9	42.5	8.6
	[35.0,57.4]	[31.8,52.8]	[15.4,31.3]	[13.3,27.2]	[1.7,8.8]	[81.3,96.3]	[37.3,60.6]	[30.9,55.0]	[3.7,18.7]
Senegal	37.1	32.9	20.7	12.2	4.2	88.7	55.9	32.8	11.3
	[30.9,44.4]	[27.1,39.9]	[16.3,26.2]	[9.8,15.2]	[2.7,6.6]	[82.8,92.8]	[48.9,62.6]	[27.5,38.7]	[7.2,17.2]
Sierra Leone	122.1	95.2	30.5	64.7	26.9	78.0	25.0	53.0	22.0
	[113.2,131.6]	[88.4,102.5]	[25.7,36.2]	[58.5,71.5]	[22.4,32.2]	[74.7,81.0]	[21.1,29.4]	[49.0,56.9]	[19.0,25.3]
Togo	71.3	48.2	27.4	20.7	23.1	67.6	38.5	29.1	32.4
	[62.9,80.8]	[41.0,56.6]	[21.9,34.4]	[16.2,26.5]	[19.0,28.1]	[61.7,72.9]	[32.2,45.2]	[23.4,35.6]	[27.1,38.3]
Latin America & Caribbean	29.9	27.0	14.7	12.3	2.9	90.3	49.1	41.2	9.7
	[28.1,31.7]	[25.2,28.9]	[13.7,15.7]	[10.8,14.0]	[2.4,3.5]	[88.3,91.9]	[45.9,52.2]	[37.7,44.8]	[8.1,11.7]
Colombia	16.3	15.4	8.5	6.8	1.0	94.0	52.3	41.7	6.0
	[13.8,19.3]	[13.0,18.1]	[6.7,10.9]	[5.4,8.6]	[0.5,1.7]	[90.1,96.4]	[43.4,60.9]	[34.2,49.7]	[3.6,9.9]
Dominican Republic	35.1	32.6	24.6	8.0	2.5	93.0	70.1	22.9	7.0

© 2022 Karlsson O et al. *JAMA Network Open*.

	[30.5,40.3]	[27.9,38.1]	[20.6,29.3]	[5.8,11.2]	[1.4,4.3]	[87.7,96.1]	[62.1,76.9]	[17.4,29.6]	[3.9,12.3]
El Salvador	19.9	18.8	11.0	7.8	1.1	94.7	55.4	39.3	5.3
	[15.1,26.0]	[14.2,24.9]	[7.2,16.7]	[5.6,10.8]	[0.5,2.4]	[88.0,97.7]	[42.6,67.5]	[27.7,52.2]	[2.3,12.0]
Guatemala	35.4	32.3	16.9	15.3	3.1	91.2	47.9	43.3	8.8
	[31.1,40.2]	[28.0,37.1]	[14.7,19.5]	[11.9,19.6]	[2.2,4.3]	[87.8,93.7]	[42.2,53.7]	[36.7,50.2]	[6.3,12.2]
Guyana	39.2	34.7	22.9	11.8	4.5	88.5	58.4	30.1	11.5
	[28.6,53.6]	[24.1,49.8]	[15.1,34.7]	[7.5,18.5]	[1.8,11.3]	[72.7,95.7]	[45.9,69.9]	[20.8,41.4]	[4.3,27.3]
Haiti	81.2	69.5	31.6	37.9	11.8	85.5	38.9	46.7	14.5
	[70.8,93.0]	[59.1,81.6]	[25.8,38.7]	[30.0,47.8]	[9.1,15.1]	[81.1,89.0]	[32.8,45.3]	[39.9,53.5]	[11.0,18.9]
Honduras	29.0	26.2	17.6	8.6	2.8	90.4	60.7	29.7	9.6
	[26.1,32.3]	[23.4,29.5]	[14.9,20.8]	[6.8,10.9]	[1.9,4.0]	[86.2,93.4]	[53.0,68.0]	[23.6,36.6]	[6.6,13.8]
Paraguay	18.5	17.7	6.0	11.7	0.8	95.8	32.5	63.2	4.2
	[14.0,24.4]	[13.3,23.5]	[4.0,9.0]	[7.5,18.1]	[0.3,2.2]	[89.1,98.4]	[20.5,47.4]	[48.5,75.8]	[1.6,10.9]
Peru	20.9	19.0	10.4	8.6	1.9	90.9	49.6	41.3	9.1
	[18.0,24.3]	[16.0,22.5]	[8.4,12.8]	[6.6,11.2]	[1.2,3.0]	[85.2,94.5]	[42.0,57.2]	[33.8,49.2]	[5.5,14.8]
Suriname	19.5	17.9	12.4	5.5	1.6	91.8	63.8	28.0	8.2
	[12.9,29.3]	[11.8,27.0]	[7.4,20.7]	[2.8,10.5]	[0.6,4.2]	[79.4,97.0]	[44.1,79.7]	[14.2,47.8]	[3.0,20.6]
Low-Income Countries	82.9	63.6	27.9	35.6	19.4	76.6	33.7	43.0	23.4
	[80.7,85.2]	[61.8,65.4]	[26.8,29.1]	[34.1,37.2]	[18.2,20.6]	[75.4,77.8]	[32.2,35.1]	[41.7,44.3]	[22.2,24.6]
Lower-Middle-Income Countries	62.3	51.0	29.2	21.9	11.2	82.0	46.9	35.1	18.0
	[60.8,63.8]	[49.8,52.3]	[28.3,30.1]	[21.0,22.7]	[10.7,11.8]	[81.3,82.7]	[45.7,48.0]	[34.2,36.0]	[17.3,18.7]
Upper-Middle-Income Countries	28.8	25.7	14.5	11.2	3.1	89.1	50.3	38.8	10.9
	[26.9,30.9]	[23.9,27.6]	[13.2,15.9]	[10.1,12.4]	[2.6,3.7]	[87.3,90.7]	[46.9,53.7]	[35.9,41.8]	[9.3,12.7]

Notes: 95% confidence intervals are shown in brackets.

© 2022 Karlsson O et al. *JAMA Network Open*.

eTable 3. Share of Deaths Among Children Younger Than 5 Years Occurring at Different Ages Using Detailed Age Intervals: Period Restricted to 5 Years Before Survey Instead of 10 Years

	<1 month	1–2 months	3–5 months	6–11 months	12–23 months	24–35 months	36–47 months	48–59 months
Pooled	43.3	8.0	6.7	11.1	11.8	9.2	6.1	3.7
	[42.4, 44.2]	[7.6, 8.4]	[6.3, 7.1]	[10.6, 11.6]	[11.4, 12.3]	[8.8, 9.7]	[5.8, 6.5]	[3.4, 4.0]
Least Developed Countries	36.8	8.2	7.2	12.8	13.3	10.5	6.7	4.4
	[35.7, 37.9]	[7.6, 8.9]	[6.6, 7.7]	[12.3, 13.5]	[12.7, 14.0]	[9.9, 11.1]	[6.2, 7.3]	[4.1, 4.8]
East Asia & Pacific	48.7	11.2	7.0	9.7	9.8	5.1	6.0	2.5
	[45.3, 52.2]	[9.3, 13.3]	[5.4, 9.1]	[7.9, 11.8]	[8.0, 11.9]	[4.0, 6.5]	[4.2, 8.6]	[1.8, 3.4]
Cambodia	51.3	15.9	5.4	7.9	7.1	2.9	6.5	3.0
	[43.0, 59.5]	[11.1, 22.4]	[2.6, 10.9]	[4.9, 12.4]	[4.6, 10.9]	[1.3, 6.4]	[4.0, 10.5]	[1.7, 5.2]
Indonesia	48.9	9.5	5.8	10.9	11.7	4.5	6.4	2.3
	[44.4, 53.4]	[6.4, 13.9]	[3.6, 9.0]	[7.9, 14.7]	[9.1, 15.0]	[2.9, 7.0]	[4.1, 9.7]	[1.3, 4.2]
Lao	39.1	25.4	15.7	7.7	3.1	3.9	2.5	2.6
	[34.2, 44.3]	[21.0, 30.2]	[12.5, 19.6]	[5.7, 10.4]	[1.7, 5.7]	[2.5, 6.0]	[1.5, 4.2]	[1.2, 5.4]
Mongolia	45.2	17.9	6.4	11.2	3.9	6.0	8.9	0.5
	[33.0, 58.1]	[9.8, 30.4]	[2.0, 18.7]	[4.6, 24.8]	[1.7, 8.8]	[2.7, 13.0]	[3.5, 20.8]	[0.1, 2.9]
Myanmar	49.1	14.9	6.9	9.8	6.0	6.5	5.8	1.1
	[39.2, 59.1]	[9.4, 22.8]	[4.1, 11.2]	[5.2, 17.7]	[3.4, 10.3]	[3.5, 11.8]	[3.1, 10.6]	[0.5, 2.6]
Papua New Guinea	42.0	5.3	9.3	11.8	9.2	12.5	7.3	2.6
	[35.3, 49.1]	[3.1, 8.7]	[5.1, 16.3]	[8.3, 16.5]	[5.5, 15.0]	[9.5, 16.3]	[4.7, 11.2]	[1.2, 5.4]
Philippines	49.9	10.6	9.2	6.8	9.5	4.8	5.5	3.7
	[40.8, 59.1]	[6.5, 16.9]	[5.7, 14.5]	[4.4, 10.4]	[6.0, 14.5]	[3.2, 7.2]	[3.1, 9.5]	[1.7, 8.0]
Timor-Leste	44.7	8.6	8.5	10.7	7.2	8.2	6.4	5.7
	[37.7, 52.0]	[5.4, 13.3]	[5.1, 13.9]	[7.1, 15.7]	[4.4, 11.6]	[5.4, 12.1]	[3.9, 10.3]	[3.2, 10.1]
Tonga	43.3	13.1	9.2	25.5	NA	3.2	5.7	NA
	[25.8, 62.7]	[1.8, 54.7]	[2.8, 26.4]	[7.6, 58.5]		[0.2, 35.4]	[0.8, 30.4]	
South Asia	58.3	9.3	6.6	8.4	7.0	4.1	3.8	2.4
	[56.9, 59.7]	[8.6, 10.1]	[5.9, 7.3]	[7.7, 9.3]	[6.3, 7.9]	[3.7, 4.5]	[3.4, 4.3]	[2.1, 2.7]
Afghanistan	40.2	13.9	11.5	15.5	8.3	5.7	3.3	1.5
	[35.9, 44.7]	[9.7, 19.5]	[9.3, 14.2]	[12.4, 19.3]	[6.4, 10.7]	[4.4, 7.4]	[2.3, 4.8]	[0.8, 2.6]
Bangladesh	65.5	7.1	6.5	5.5	5.1	4.9	2.9	2.4
	[61.6, 69.3]	[5.6, 9.1]	[5.0, 8.5]	[4.2, 7.3]	[3.6, 7.2]	[3.3, 7.1]	[1.8, 4.5]	[1.5, 4.0]
India	59.2	8.6	6.4	7.7	7.2	4.2	4.2	2.6
	[57.9, 60.6]	[8.1, 9.1]	[5.8, 7.0]	[7.1, 8.3]	[6.6, 7.9]	[3.8, 4.5]	[3.9, 4.6]	[2.3, 2.9]
Maldives	55.8	10.1	12.7	10.2	2.1	2.8	3.3	2.9
	[34.2, 75.4]	[3.8, 24.4]	[4.1, 33.1]	[2.3, 35.1]	[0.6, 6.8]	[0.7, 10.5]	[1.0, 10.8]	[0.8, 10.5]
Nepal	55.9	14.7	7.5	8.8	2.8	3.7	2.0	4.7
	[48.2, 63.3]	[9.4, 22.1]	[4.2, 13.1]	[5.2, 14.5]	[1.1, 7.1]	[1.7, 8.1]	[0.6, 6.6]	[2.1, 10.0]
Pakistan	56.5	11.3	6.2	10.4	7.1	3.5	3.0	2.0
	[51.3, 61.5]	[8.6, 14.7]	[4.3, 8.9]	[8.0, 13.4]	[5.1, 9.7]	[2.2, 5.5]	[1.8, 4.9]	[1.2, 3.3]
Europe & Central Asia	50.5	6.1	11.6	18.0	5.5	4.7	2.7	1.1
	[43.7, 57.2]	[4.0, 9.1]	[7.2, 18.1]	[11.6, 26.7]	[3.3, 9.0]	[2.6, 8.3]	[1.4, 5.0]	[0.5, 2.4]
Albania	37.3	NA	4.1	34.8	23.8	NA	NA	NA
	[5.6, 85.6]		[0.1, 74.0]	[5.8, 82.2]	[4.6, 67.1]			
Armenia	48.5	NA	NA	17.3	17.4	14.5	2.2	NA
	[23.1, 74.7]			[4.2, 50.0]	[4.5, 48.5]	[2.8, 50.3]	[0.1, 26.6]	
Kyrgyz Republic	68.5	11.0	1.1	6.1	1.5	8.9	2.6	0.4
	[55.0, 79.5]	[4.9, 22.8]	[0.1, 11.7]	[2.0, 17.3]	[0.4, 5.6]	[3.1, 22.9]	[0.6, 10.5]	[0.1, 2.7]
Moldova	81.6	8.3	NA	NA	NA	8.5	NA	1.6

© 2022 Karlsson O et al. *JAMA Network Open*.

	[58.4, 93.3]	[1.2, 40.7]				[1.7, 32.7]		[0.1, 18.7]
Tajikistan	38.4	8.6	10.5	23.3	8.7	4.4	3.0	3.1
	[30.0, 47.5]	[5.4, 13.3]	[6.2, 17.2]	[17.8, 29.9]	[5.5, 13.7]	[2.0, 9.4]	[1.3, 6.9]	[1.2, 7.9]
Turkey	49.7	4.2	14.4	20.7	4.9	3.8	2.2	NA
	[32.1, 67.4]	[1.4, 11.7]	[6.8, 28.0]	[8.6, 42.0]	[0.7, 26.2]	[1.3, 10.9]	[0.6, 7.8]	
Turkmenistan	58.4	5.2	12.0	8.8	3.8	5.1	4.3	2.4
	[48.0, 68.0]	[2.9, 9.3]	[6.4, 21.3]	[5.0, 15.1]	[1.5, 9.4]	[2.5, 9.9]	[1.8, 9.6]	[0.5, 10.9]
Middle East & North Africa	51.3	10.9	9.4	11.3	7.4	4.6	3.3	1.8
	[48.3, 54.2]	[9.3, 12.9]	[7.7, 11.6]	[9.3, 13.5]	[6.1, 9.0]	[3.4, 6.1]	[2.3, 4.8]	[1.1, 2.8]
Egypt	50.1	12.3	9.5	9.2	7.7	5.3	4.5	1.5
	[44.8, 55.4]	[9.4, 15.8]	[7.1, 12.4]	[6.0, 13.7]	[5.6, 10.6]	[3.4, 8.2]	[2.5, 7.7]	[0.5, 4.5]
Iraq	56.1	7.7	10.6	13.2	3.4	3.4	3.9	1.8
	[49.8, 62.2]	[5.5, 10.9]	[6.8, 15.9]	[9.7, 17.6]	[2.0, 5.6]	[2.0, 5.7]	[1.9, 7.8]	[1.1, 3.1]
Jordan	55.5	4.7	10.9	15.7	4.1	4.7	3.9	0.6
	[42.3, 67.9]	[2.3, 9.6]	[5.6, 19.9]	[8.7, 26.6]	[2.0, 8.5]	[3.0, 7.3]	[1.2, 11.8]	[0.1, 4.2]
State of Palestine	51.4	14.8	8.0	9.8	8.3	3.2	2.9	1.7
	[44.1, 58.7]	[10.3, 20.7]	[4.7, 13.1]	[5.9, 15.8]	[5.2, 13.2]	[1.3, 7.3]	[1.0, 7.9]	[0.5, 5.2]
Tunisia	51.8	14.0	4.5	13.0	6.3	4.7	4.0	1.7
	[37.0, 66.3]	[7.5, 24.8]	[1.4, 13.3]	[6.0, 26.0]	[2.1, 17.3]	[1.9, 10.8]	[1.2, 13.1]	[0.2, 12.9]
Yemen	49.1	10.8	9.0	13.0	10.2	4.5	1.1	2.3
	[44.0, 54.2]	[8.2, 14.1]	[6.8, 12.0]	[10.9, 15.5]	[7.9, 13.0]	[3.0, 6.7]	[0.5, 2.2]	[1.4, 3.7]
Eastern & Southern Africa	40.8	9.0	7.7	12.1	12.6	8.0	5.9	3.9
	[38.9, 42.8]	[8.1, 10.1]	[6.9, 8.5]	[11.3, 13.1]	[11.6, 13.6]	[7.4, 8.6]	[5.1, 6.7]	[3.4, 4.5]
Angola	35.4	10.6	5.2	13.7	12.1	10.7	8.4	4.0
	[31.2, 39.8]	[8.1, 13.6]	[3.8, 6.9]	[10.9, 17.0]	[9.5, 15.2]	[8.4, 13.5]	[6.0, 11.8]	[2.9, 5.5]
Burundi	29.4	11.1	6.8	13.0	15.5	9.2	10.2	4.9
	[25.5, 33.6]	[8.9, 13.9]	[5.2, 8.7]	[10.9, 15.4]	[13.0, 18.4]	[7.1, 11.7]	[8.4, 12.3]	[3.6, 6.6]
Comoros	47.3	13.6	6.3	4.8	5.5	10.7	7.6	4.1
	[39.1, 55.7]	[7.4, 23.7]	[3.6, 10.7]	[2.0, 11.3]	[2.8, 10.7]	[4.8, 22.0]	[4.0, 14.1]	[1.6, 10.4]
Ethiopia	44.0	11.0	6.3	10.5	11.1	6.9	6.2	4.1
	[37.0, 51.2]	[7.5, 15.9]	[4.0, 9.6]	[8.3, 13.1]	[8.3, 14.8]	[4.8, 9.7]	[4.0, 9.4]	[2.6, 6.5]
Kenya	42.6	8.4	10.0	12.9	10.8	5.6	6.2	3.5
	[38.1, 47.2]	[6.6, 10.7]	[8.1, 12.3]	[10.6, 15.6]	[8.3, 13.8]	[4.0, 7.7]	[4.1, 9.1]	[2.4, 5.2]
Lesotho	47.8	8.7	11.1	14.3	4.8	6.4	3.6	3.3
	[39.8, 55.9]	[5.5, 13.4]	[6.8, 17.5]	[9.5, 21.1]	[2.6, 8.8]	[3.8, 10.5]	[0.9, 13.0]	[1.4, 7.7]
Madagascar	35.9	11.0	7.1	13.8	6.6	11.4	7.8	6.4
	[31.7, 40.2]	[8.4, 14.3]	[5.3, 9.6]	[11.3, 16.7]	[4.9, 8.9]	[9.2, 14.0]	[5.5, 10.8]	[4.6, 8.8]
Malawi	41.8	7.2	4.9	11.5	11.5	11.6	5.7	5.8
	[37.1, 46.6]	[5.4, 9.5]	[3.7, 6.5]	[9.4, 14.0]	[9.5, 13.9]	[9.1, 14.7]	[4.2, 7.7]	[4.0, 8.4]
Mozambique	31.4	10.0	8.4	16.3	14.4	9.5	6.7	3.3
	[27.6, 35.5]	[8.0, 12.4]	[6.7, 10.5]	[13.4, 19.7]	[11.0, 18.6]	[7.6, 11.9]	[5.0, 8.8]	[2.4, 4.5]
Namibia	36.2	10.2	9.7	15.2	12.8	6.6	6.2	2.9
	[28.9, 44.2]	[6.9, 14.9]	[6.6, 14.1]	[10.9, 20.8]	[8.9, 18.3]	[3.6, 12.0]	[3.8, 9.9]	[1.4, 5.9]
Rwanda	38.9	6.8	5.7	12.7	14.5	7.9	6.3	7.2
	[34.0, 44.0]	[4.7, 9.7]	[4.0, 8.1]	[10.0, 16.1]	[11.4, 18.3]	[5.8, 10.8]	[4.2, 9.3]	[5.2, 9.8]
South Africa	50.0	5.5	17.2	11.7	6.4	3.7	2.1	3.4
	[39.6, 60.4]	[2.9, 10.1]	[10.1, 27.6]	[7.0, 18.8]	[2.7, 14.5]	[1.9, 7.2]	[0.6, 6.8]	[1.6, 7.0]
South Sudan	39.4	10.2	12.2	10.8	11.1	7.4	4.6	4.4
	[34.5, 44.5]	[8.1, 12.7]	[9.9, 14.9]	[8.9, 13.1]	[9.2, 13.4]	[6.0, 9.2]	[3.4, 6.2]	[3.2, 6.0]
Sudan	47.6	9.0	8.1	11.2	11.9	6.8	2.9	2.4
	[43.4, 51.9]	[6.2, 13.0]	[6.5, 10.2]	[8.4, 14.8]	[9.6, 14.5]	[5.3, 8.8]	[2.0, 4.3]	[1.6, 3.6]
Swaziland	30.0	9.2	9.4	25.6	11.5	6.3	5.6	2.4

© 2022 Karlsson O et al. *JAMA Network Open*.

	[22.5, 38.9]	[5.1, 16.2]	[5.8, 15.1]	[20.2, 31.8]	[7.0, 18.2]	[2.9, 13.2]	[2.3, 13.1]	[0.6, 8.8]
Tanzania	37.9	6.2	7.4	12.9	18.8	7.6	5.7	3.5
	[32.8, 43.2]	[4.1, 9.4]	[5.6, 9.7]	[9.9, 16.6]	[14.8, 23.6]	[5.7, 10.1]	[4.2, 7.8]	[2.1, 5.6]
Uganda	41.6	7.9	6.4	10.7	14.4	9.7	5.8	3.5
	[39.0, 44.2]	[6.1, 10.1]	[4.9, 8.4]	[8.5, 13.5]	[12.4, 16.6]	[7.9, 11.8]	[4.8, 7.1]	[2.6, 4.7]
Zambia	45.4	9.0	5.5	9.3	12.6	8.4	6.0	3.7
	[40.4, 50.5]	[6.3, 12.7]	[3.8, 8.0]	[7.3, 11.9]	[9.2, 17.0]	[6.0, 11.7]	[4.1, 8.7]	[2.1, 6.2]
Zimbabwe	49.8	7.5	5.2	9.5	13.2	6.7	4.0	4.1
	[44.3, 55.3]	[5.3, 10.7]	[3.6, 7.5]	[6.7, 13.3]	[9.9, 17.2]	[4.5, 9.9]	[2.3, 7.1]	[2.6, 6.4]
West & Central Africa	29.5	5.4	5.9	12.8	16.4	15.7	8.9	5.4
	[28.4, 30.7]	[5.0, 5.8]	[5.4, 6.5]	[12.1, 13.5]	[15.6, 17.4]	[14.9, 16.5]	[8.4, 9.4]	[5.0, 5.9]
Benin	31.2	5.9	6.0	14.1	13.8	12.7	11.0	5.4
	[28.2, 34.4]	[4.3, 7.9]	[4.7, 7.5]	[11.9, 16.7]	[11.6, 16.4]	[11.0, 14.7]	[9.2, 13.0]	[4.0, 7.1]
Burkina Faso	21.8	5.2	7.0	16.6	18.8	16.0	9.0	5.5
	[20.0, 23.8]	[4.2, 6.5]	[5.7, 8.6]	[14.9, 18.5]	[16.8, 21.0]	[14.4, 17.8]	[7.8, 10.4]	[4.3, 6.9]
Cameroon	35.3	7.3	5.8	11.5	14.8	11.0	9.5	4.9
	[31.3, 39.5]	[5.3, 10.1]	[4.3, 7.8]	[9.4, 13.9]	[12.2, 17.8]	[8.8, 13.6]	[7.2, 12.4]	[3.4, 7.0]
Chad	25.4	6.3	7.0	15.6	17.1	14.7	8.7	5.1
	[23.0, 28.0]	[5.3, 7.5]	[5.7, 8.5]	[13.8, 17.6]	[15.0, 19.4]	[13.0, 16.7]	[7.2, 10.6]	[4.1, 6.3]
Congo	31.8	6.4	8.7	11.3	16.2	12.6	8.0	5.1
	[25.9, 38.3]	[4.2, 9.5]	[5.8, 12.7]	[7.5, 16.7]	[12.4, 21.0]	[9.4, 16.8]	[5.4, 11.8]	[3.2, 7.9]
Congo (DR)	26.8	5.1	7.5	16.5	15.1	13.7	8.4	7.0
	[23.9, 30.0]	[3.9, 6.6]	[6.1, 9.3]	[14.7, 18.6]	[12.8, 17.7]	[11.6, 16.1]	[6.9, 10.1]	[5.4, 9.0]
Cote D'Ivoire	34.6	6.8	8.2	12.5	15.0	11.8	7.0	4.0
	[31.0, 38.4]	[5.2, 8.9]	[6.3, 10.7]	[10.3, 15.2]	[12.4, 18.0]	[9.5, 14.4]	[5.3, 9.4]	[2.7, 5.8]
Gabon	40.7	2.5	5.4	17.1	11.1	9.7	7.9	5.4
	[32.3, 49.7]	[1.2, 5.4]	[3.3, 8.8]	[12.8, 22.6]	[7.2, 16.7]	[6.2, 14.9]	[3.8, 15.8]	[2.3, 12.3]
Gambia	53.5	5.2	5.5	7.4	10.2	8.3	6.0	3.8
	[47.0, 59.9]	[3.7, 7.3]	[3.9, 7.8]	[5.0, 10.7]	[7.4, 14.1]	[6.5, 10.6]	[3.9, 9.1]	[2.2, 6.4]
Ghana	47.9	5.3	7.0	8.6	9.1	8.0	7.3	6.6
	[41.4, 54.5]	[3.3, 8.4]	[3.9, 12.3]	[5.8, 12.8]	[6.1, 13.5]	[5.3, 12.0]	[3.5, 14.5]	[3.7, 11.6]
Guinea	29.0	8.6	8.0	14.2	14.7	14.7	6.4	4.6
	[25.4, 32.9]	[6.7, 10.9]	[6.0, 10.7]	[11.7, 17.0]	[12.1, 17.6]	[11.9, 18.0]	[4.8, 8.4]	[3.3, 6.3]
Liberia	28.0	6.1	10.0	13.2	20.7	10.3	7.0	4.7
	[24.0, 32.3]	[4.5, 8.2]	[7.1, 13.9]	[10.2, 17.1]	[16.8, 25.3]	[7.8, 13.5]	[4.7, 10.3]	[2.9, 7.5]
Mali	32.2	5.2	5.8	10.6	15.0	15.9	9.3	6.0
	[28.3, 36.3]	[4.0, 6.8]	[4.3, 7.8]	[8.9, 12.7]	[12.6, 17.9]	[13.1, 19.2]	[7.3, 11.8]	[4.8, 7.3]
Mauritania	53.7	12.5	5.7	7.9	5.0	7.9	4.2	3.2
	[48.9, 58.5]	[9.9, 15.7]	[3.8, 8.6]	[5.7, 10.8]	[3.0, 8.1]	[5.7, 10.7]	[2.0, 8.5]	[1.9, 5.4]
Niger	19.0	4.6	4.4	11.8	20.3	19.7	12.0	8.1
	[16.7, 21.5]	[3.5, 6.1]	[3.3, 5.8]	[10.1, 13.6]	[18.4, 22.5]	[17.6, 21.9]	[10.5, 13.8]	[6.7, 9.8]
Nigeria	29.7	4.9	4.8	11.5	17.5	17.5	9.0	5.0
	[27.3, 32.3]	[4.3, 5.6]	[4.2, 5.6]	[10.2, 13.0]	[16.1, 19.0]	[16.0, 19.2]	[8.0, 10.2]	[4.2, 6.0]
Sao Tome & Principe	48.9	7.7	11.9	16.7	6.1	4.3	3.2	1.1
	[37.3, 60.6]	[3.3, 16.9]	[6.2, 21.7]	[11.0, 24.7]	[2.6, 13.8]	[1.6, 10.8]	[1.1, 8.9]	[0.2, 6.1]
Senegal	55.9	8.8	4.8	8.9	10.3	7.1	3.2	1.0
	[48.9, 62.6]	[5.9, 12.8]	[2.4, 9.5]	[6.0, 13.1]	[7.3, 14.4]	[3.9, 12.5]	[1.9, 5.4]	[0.4, 2.9]
Sierra Leone	25.0	10.4	9.3	17.2	16.2	13.3	5.1	3.6
	[21.1, 29.4]	[8.4, 12.7]	[7.4, 11.5]	[14.9, 19.6]	[14.1, 18.7]	[11.1, 15.7]	[3.8, 6.9]	[2.6, 4.9]
Togo	38.5	4.4	6.1	9.4	9.2	14.6	13.0	4.7
	[32.2, 45.2]	[2.3, 8.1]	[4.1, 8.9]	[6.5, 13.5]	[5.6, 14.8]	[11.5, 18.4]	[9.3, 17.9]	[2.9, 7.6]
Latin America & Caribbean	49.1	11.2	8.7	11.0	10.3	5.0	3.2	1.6

© 2022 Karlsson O et al. *JAMA Network Open*.

	[45.9, 52.2]	[9.6, 13.1]	[6.8, 10.9]	[8.8, 13.7]	[8.9, 12.0]	[4.0, 6.2]	[2.3, 4.4]	[1.1, 2.3]
Colombia	52.3	12.8	10.0	10.1	8.8	3.3	1.3	1.4
	[43.4, 60.9]	[8.0, 19.9]	[6.0, 16.1]	[6.2, 16.1]	[5.5, 13.8]	[1.5, 7.2]	[0.4, 3.6]	[0.6, 3.2]
Dominican Republic	70.1	6.5	4.5	7.7	4.2	2.7	1.5	2.8
	[62.1, 76.9]	[4.4, 9.5]	[2.1, 9.2]	[4.7, 12.3]	[1.8, 9.4]	[1.2, 6.3]	[0.6, 3.7]	[0.9, 7.9]
El Salvador	55.4	13.3	8.2	10.6	7.2	4.5	0.8	NA
	[42.6, 67.5]	[6.1, 26.6]	[3.4, 18.2]	[5.5, 19.4]	[2.7, 17.8]	[1.9, 10.5]	[0.1, 5.3]	
Guatemala	47.9	12.6	9.8	8.3	12.7	4.9	2.8	1.0
	[42.2, 53.7]	[8.4, 18.5]	[6.8, 13.9]	[5.7, 12.0]	[9.6, 16.5]	[3.2, 7.5]	[1.6, 4.9]	[0.4, 2.3]
Guyana	58.4	7.4	8.9	6.3	7.6	2.3	5.7	3.5
	[45.9, 69.9]	[3.7, 14.0]	[4.1, 18.2]	[2.7, 13.7]	[2.3, 22.2]	[0.6, 8.1]	[1.2, 23.3]	[0.6, 16.8]
Haiti	38.9	10.4	8.9	13.8	13.5	7.6	5.2	1.7
	[32.8, 45.3]	[8.0, 13.4]	[5.9, 13.4]	[10.5, 18.1]	[10.5, 17.1]	[5.2, 11.0]	[3.1, 8.5]	[0.9, 3.1]
Honduras	60.7	6.2	6.1	8.3	9.1	3.7	3.6	2.3
	[53.0, 68.0]	[4.4, 8.7]	[3.8, 9.7]	[5.5, 12.2]	[6.2, 13.2]	[2.0, 6.8]	[1.8, 7.3]	[1.1, 4.5]
Paraguay	32.5	19.0	22.8	19.7	1.7	2.6	1.0	0.7
	[20.5, 47.4]	[7.1, 42.0]	[11.8, 39.3]	[7.3, 43.2]	[0.4, 6.4]	[0.7, 9.2]	[0.2, 5.8]	[0.1, 8.1]
Peru	49.6	13.4	6.5	11.5	10.0	4.5	3.0	1.6
	[42.0, 57.2]	[8.6, 20.2]	[3.3, 12.4]	[5.4, 22.9]	[5.8, 16.5]	[2.3, 8.8]	[1.1, 7.7]	[0.6, 4.2]
Suriname	63.8	7.2	6.8	11.6	2.4	1.4	6.8	NA
	[44.1, 79.7]	[1.8, 25.2]	[1.5, 25.4]	[3.6, 31.8]	[0.7, 7.7]	[0.2, 8.6]	[2.1, 19.7]	
Low-Income Countries	33.7	7.9	7.4	13.7	14.0	11.4	7.1	4.9
	[32.2, 35.1]	[7.3, 8.6]	[6.7, 8.0]	[12.9, 14.5]	[13.3, 14.8]	[10.6, 12.2]	[6.6, 7.6]	[4.4, 5.4]
Lower-Middle-Income Countries	46.9	7.9	6.2	9.8	11.1	8.7	5.9	3.4
	[45.7, 48.0]	[7.4, 8.4]	[5.8, 6.6]	[9.3, 10.4]	[10.6, 11.7]	[8.3, 9.3]	[5.5, 6.3]	[3.1, 3.7]
Upper-Middle-Income Countries	50.3	8.6	9.3	11.8	9.1	4.2	4.5	2.2
	[46.9, 53.7]	[7.1, 10.3]	[7.7, 11.2]	[9.4, 14.7]	[7.6, 10.8]	[3.3, 5.3]	[3.3, 6.0]	[1.5, 3.1]

Notes: 95% confidence intervals are shown in brackets.

© 2022 Karlsson O et al. *JAMA Network Open*.

eTable 4. Component Mortality Probabilities for Pooled Sample

Age interval	Weighted cases (unweighted)			Weighted deaths (unweighted)			Component probability	1–Component probability
	Cohort A	Cohort B	Cohort C	Cohort A	Cohort B	Cohort C		
< 1 month	2,671.6	614,416.1	2,734.0	186.0	17,364.3	82.8	0.0284	0.9716
	(17,086)	(1,987,110)	(17,589)	(536)	(51,975)	(491)		
1–2 months	5,270.7	591,812.9	5,252.0	68.9	3,255.4	23.8	0.0056	0.9944
	(34,794)	(1,917,439)	(34,193)	(215)	(10,973)	(185)		
3–5 months	8,049.0	582,915.6	7,733.1	82.8	2,775.1	45.8	0.0049	0.9951
	(52,289)	(1,888,185)	(49,373)	(282)	(9,251)	(291)		
6–11 months	15,158.2	565,198.7	13,605.6	183.1	4,396.6	132.4	0.0079	0.9921
	(98,992)	(1,829,024)	(87,175)	(606)	(14,169)	(847)		
12–23 months	29,615.2	528,518.0	24,705.4	299.9	4,203.2	229.2	0.0081	0.9919
	(191,907)	(1,709,276)	(157,624)	(934)	(12,857)	(1,473)		
24–35 months	29,204.3	514,858.7	26,780.8	242.8	3,166.7	183.0	0.0063	0.9937
	(191,912)	(1,660,658)	(170,080)	(788)	(9,680)	(1,145)		
36–47 months	29,931.7	505,024.4	24,285.2	182.8	2,055.3	125.8	0.0042	0.9958
	(195,287)	(1,624,626)	(153,690)	(600)	(6,284)	(709)		
48–59 months	29,150.3	492,987.4	22,993.9	118.6	1,153.3	64.3	0.0025	0.9975
	(188,737)	(1,582,586)	(145,590)	(380)	(3,616)	(388)		
								U5MR=66

Notes: Unweighted cases and deaths are shown in parentheses below weighted cases and deaths. Acronyms: under-5 mortality rate (U5MR).

© 2022 Karlsson O et al. *JAMA Network Open*.

eTable 5. Mortality and the Share of Deaths Among Children Younger Than 5 Years by Age

	Mortality (deaths per 1000)					Share (%)			
	0–59 months	0–23 months	Neonates	1–23 months	24–59 months	0–23 months	Neonates	1–23 months	24–59 months
Pooled	66.1	53.9	28.4	25.5	12.2	81.5	43.0	38.5	18.5
	[65.1,67.1]	[53.1,54.8]	[27.9,29.0]	[24.9,26.1]	[11.8,12.6]	[81.0,82.0]	[42.4,43.7]	[37.9,39.1]	[18.0,19.0]
Least Developed Countries	81.2	64.1	29.4	34.8	17.1	79.0	36.2	42.8	21.0
	[79.6,82.8]	[62.8,65.5]	[28.5,30.2]	[33.8,35.7]	[16.5,17.6]	[78.4,79.6]	[35.4,37.0]	[42.0,43.6]	[20.4,21.6]
East Asia & Pacific	37.8	33.0	18.3	14.7	4.8	87.4	48.5	38.9	12.6
	[35.8,39.8]	[31.2,34.9]	[17.1,19.5]	[13.6,15.9]	[4.1,5.5]	[85.7,88.9]	[46.3,50.7]	[37.0,40.9]	[11.1,14.3]
Cambodia	47.7	41.9	21.1	20.8	5.9	87.7	44.2	43.5	12.3
	[42.2,54.0]	[36.6,47.9]	[18.2,24.4]	[17.4,24.8]	[4.6,7.5]	[84.5,90.3]	[40.2,48.3]	[39.2,47.8]	[9.7,15.5]
Indonesia	34.0	29.8	17.3	12.5	4.2	87.6	50.9	36.7	12.4
	[31.9,36.3]	[28.0,31.8]	[16.1,18.6]	[11.2,13.9]	[3.2,5.5]	[84.4,90.2]	[47.8,54.0]	[33.7,39.7]	[9.8,15.6]
Lao	49.3	45.0	20.3	24.7	4.3	91.3	41.2	50.1	8.7
	[45.1,53.9]	[40.9,49.5]	[17.8,23.2]	[21.8,27.9]	[3.6,5.2]	[89.5,92.8]	[37.3,45.2]	[46.3,53.8]	[7.2,10.5]
Mongolia	18.2	15.2	8.0	7.2	3.0	83.5	43.9	39.6	16.5
	[15.2,21.9]	[12.3,18.7]	[6.1,10.4]	[5.5,9.5]	[1.8,4.9]	[74.6,89.7]	[35.3,52.8]	[32.6,47.1]	[10.3,25.4]
Myanmar	71.9	63.2	31.9	31.3	8.7	88.0	44.4	43.5	12.0
	[64.0,80.6]	[56.3,70.9]	[27.7,36.7]	[26.0,37.6]	[6.7,11.2]	[85.1,90.4]	[38.6,50.4]	[38.7,48.5]	[9.6,14.9]
Papua New Guinea	51.9	41.9	23.7	18.2	10.0	80.7	45.7	35.1	19.3
	[46.6,57.7]	[37.2,47.2]	[20.9,26.9]	[14.5,22.9]	[7.6,13.1]	[75.8,84.9]	[39.7,51.7]	[29.7,40.8]	[15.1,24.2]
Philippines	28.0	24.4	13.8	10.6	3.6	87.2	49.4	37.8	12.8
	[24.9,31.4]	[21.5,27.6]	[11.6,16.4]	[9.0,12.5]	[2.7,4.7]	[83.6,90.1]	[44.1,54.6]	[33.0,42.9]	[9.9,16.4]
Timor-Leste	40.6	32.6	16.9	15.7	8.0	80.2	41.6	38.6	19.8
	[36.7,45.0]	[29.4,36.1]	[14.2,20.2]	[13.6,18.1]	[6.3,10.3]	[75.7,84.0]	[36.2,47.2]	[33.4,44.1]	[16.0,24.3]
Tonga	11.2	10.5	5.1	5.3	0.8	93.2	45.6	47.6	6.8
	[7.8,16.2]	[7.0,15.6]	[3.5,7.4]	[2.8,10.1]	[0.2,2.8]	[75.6,98.4]	[31.0,60.9]	[29.9,65.9]	[1.6,24.4]
South Asia	54.8	49.0	31.7	17.3	5.8	89.4	57.7	31.6	10.6
	[53.6,56.1]	[47.8,50.3]	[30.7,32.6]	[16.6,18.1]	[5.6,6.1]	[88.9,89.8]	[56.7,58.8]	[30.6,32.7]	[10.2,11.1]
Afghanistan	62.0	55.5	24.9	30.6	6.5	89.5	40.1	49.4	10.5
	[56.9,67.6]	[50.8,60.6]	[22.5,27.4]	[27.3,34.4]	[5.6,7.7]	[88.0,90.8]	[37.1,43.1]	[46.6,52.1]	[9.2,12.0]
Bangladesh	40.5	36.1	25.9	10.1	4.5	89.0	64.0	25.0	11.0
	[38.6,42.5]	[34.2,38.0]	[24.4,27.6]	[8.9,11.5]	[3.9,5.2]	[87.3,90.4]	[61.1,66.7]	[22.3,27.9]	[9.6,12.7]
India	52.2	46.4	30.5	15.9	5.8	88.8	58.4	30.4	11.2
	[51.4,53.0]	[45.6,47.2]	[29.7,31.3]	[15.4,16.3]	[5.6,6.0]	[88.4,89.3]	[57.5,59.4]	[29.6,31.3]	[10.7,11.6]
Maldives	21.6	19.4	12.6	6.8	2.2	89.6	58.3	31.3	10.4
	[17.5,26.7]	[15.3,24.5]	[9.6,16.5]	[3.8,11.9]	[1.2,4.2]	[81.2,94.5]	[43.7,71.7]	[19.3,46.4]	[5.5,18.8]
Nepal	32.9	29.3	17.7	11.6	3.6	89.1	53.8	35.3	10.9
	[28.9,37.4]	[25.4,33.7]	[14.9,20.9]	[9.5,14.2]	[2.3,5.6]	[83.5,92.9]	[48.1,59.4]	[30.1,40.9]	[7.1,16.5]
Pakistan	74.2	67.5	42.4	25.1	6.7	91.0	57.1	33.8	9.0
	[67.7,81.3]	[61.5,74.1]	[38.6,46.5]	[21.3,29.6]	[5.5,8.2]	[89.2,92.5]	[53.3,60.8]	[30.2,37.7]	[7.5,10.8]
Europe & Central Asia	22.7	20.8	11.1	9.7	1.9	91.5	49.0	42.6	8.5
	[19.8,26.0]	[18.0,23.9]	[9.4,13.1]	[7.9,11.8]	[1.3,2.9]	[87.5,94.4]	[43.2,54.8]	[37.5,47.8]	[5.6,12.5]
Albania	5.8	5.7	3.4	2.3	0.1	97.8	58.3	39.5	2.2
	[3.7,9.0]	[3.6,8.9]	[1.8,6.3]	[1.0,5.1]	[0.0,0.7]	[85.9,99.7]	[32.7,80.1]	[18.2,65.7]	[0.3,14.1]
Armenia	10.5	9.6	5.3	4.3	0.9	91.4	50.7	40.7	8.6
	[7.6,14.6]	[7.0,13.2]	[3.3,8.5]	[2.4,7.5]	[0.3,2.6]	[78.3,96.9]	[31.7,69.5]	[24.1,59.8]	[3.1,21.7]
Kyrgyz Republic	22.3	20.6	14.2	6.4	1.7	92.4	63.6	28.9	7.6
	[18.4,26.9]	[16.5,25.6]	[10.4,19.2]	[4.4,9.4]	[0.9,3.2]	[85.1,96.3]	[51.1,74.4]	[19.3,40.7]	[3.7,14.9]
Moldova	15.7	14.9	11.8	3.0	0.9	94.4	75.2	19.1	5.6
	[11.4,21.8]	[10.9,20.2]	[8.5,16.6]	[1.5,6.0]	[0.2,3.4]	[80.5,98.5]	[61.1,85.5]	[9.8,33.8]	[1.5,19.5]
Tajikistan	33.3	30.3	14.9	15.4	3.1	90.7	44.6	46.1	9.3

© 2022 Karlsson O et al. *JAMA Network Open*.

	[30.3,36.7]	[27.6,33.2]	[12.6,17.5]	[13.0,18.1]	[2.0,4.8]	[86.2,93.9]	[38.1,51.3]	[39.9,52.5]	[6.1,13.8]
Turkey	20.1	18.5	9.3	9.2	1.6	92.1	46.3	45.7	7.9
	[16.3,24.7]	[14.8,23.0]	[7.0,12.3]	[6.7,12.6]	[0.8,3.2]	[84.4,96.1]	[36.4,56.5]	[37.0,54.8]	[3.9,15.6]
Turkmenistan	35.8	31.4	19.3	12.1	4.4	87.8	54.0	33.9	12.2
	[30.9,41.4]	[27.0,36.5]	[15.9,23.4]	[9.3,15.8]	[3.0,6.4]	[82.9,91.4]	[46.7,61.0]	[26.8,41.7]	[8.6,17.1]
Middle East & North Africa	32.1	29.3	16.5	12.8	2.8	91.3	51.4	39.9	8.7
	[30.3,33.9]	[27.5,31.2]	[15.1,17.9]	[12.0,13.7]	[2.4,3.2]	[90.0,92.5]	[49.1,53.7]	[37.9,41.9]	[7.5,10.0]
Egypt	30.3	27.7	16.3	11.4	2.6	91.6	53.8	37.7	8.4
	[27.8,32.9]	[25.2,30.4]	[14.5,18.3]	[10.0,13.0]	[2.0,3.3]	[89.0,93.5]	[50.2,57.4]	[34.1,41.5]	[6.5,11.0]
Iraq	24.5	22.1	12.9	9.2	2.3	90.4	52.7	37.7	9.6
	[21.4,28.0]	[19.3,25.4]	[11.0,15.1]	[7.8,11.0]	[1.7,3.2]	[87.4,92.8]	[48.6,56.8]	[33.6,42.1]	[7.2,12.6]
Jordan	16.7	15.4	9.3	6.2	1.3	92.2	55.4	36.8	7.8
	[13.9,20.0]	[12.8,18.6]	[7.4,11.6]	[4.6,8.3]	[0.8,2.2]	[87.4,95.2]	[46.4,64.0]	[29.0,45.3]	[4.8,12.6]
State of Palestine	22.8	20.7	11.5	9.2	2.1	90.6	50.2	40.4	9.4
	[20.1,26.0]	[17.9,23.9]	[9.7,13.6]	[7.4,11.4]	[1.5,3.2]	[86.1,93.7]	[44.2,56.1]	[34.8,46.2]	[6.3,13.9]
Tunisia	15.8	14.6	9.5	5.1	1.3	92.0	59.8	32.2	8.0
	[13.0,19.3]	[11.7,18.1]	[7.3,12.3]	[3.5,7.4]	[0.7,2.3]	[85.5,95.7]	[47.7,70.8]	[23.5,42.3]	[4.3,14.5]
Yemen	57.5	52.6	26.5	26.1	5.0	91.4	46.1	45.3	8.6
	[53.4,62.0]	[48.8,56.6]	[24.1,29.2]	[23.5,28.8]	[3.8,6.4]	[89.2,93.2]	[43.1,49.2]	[42.1,48.5]	[6.8,10.8]
Eastern & Southern Africa	73.8	60.5	29.1	31.4	13.3	82.0	39.4	42.6	18.0
	[71.7,76.0]	[58.7,62.4]	[27.8,30.4]	[30.2,32.7]	[12.5,14.2]	[81.0,82.9]	[38.0,40.9]	[41.4,43.8]	[17.1,19.0]
Angola	79.7	59.8	24.4	35.4	19.9	75.0	30.6	44.4	25.0
	[72.4,87.7]	[54.0,66.1]	[21.3,28.0]	[31.4,39.9]	[17.0,23.4]	[71.9,77.9]	[27.6,33.9]	[41.2,47.6]	[22.1,28.1]
Burundi	82.0	62.2	25.0	37.3	19.8	75.9	30.5	45.4	24.1
	[78.3,85.9]	[58.5,66.2]	[22.3,27.9]	[34.9,39.7]	[17.8,21.9]	[73.3,78.3]	[27.8,33.3]	[43.2,47.6]	[21.7,26.7]
Comoros	49.8	41.7	25.4	16.3	8.0	83.9	51.0	32.8	16.1
	[42.0,58.9]	[34.9,49.8]	[20.6,31.3]	[12.2,21.8]	[5.6,11.5]	[78.3,88.2]	[43.9,58.1]	[26.0,40.5]	[11.8,21.7]
Ethiopia	81.5	68.5	38.0	30.5	13.0	84.0	46.7	37.4	16.0
	[75.4,88.0]	[63.8,73.5]	[34.3,42.2]	[26.7,34.7]	[10.7,15.8]	[81.5,86.3]	[42.4,51.0]	[33.5,41.4]	[13.7,18.5]
Kenya	56.0	46.9	22.9	23.9	9.1	83.7	41.0	42.7	16.3
	[53.2,59.0]	[44.2,49.7]	[21.0,25.1]	[21.8,26.3]	[7.7,10.8]	[81.0,86.1]	[37.6,44.4]	[39.7,45.9]	[13.9,19.0]
Lesotho	86.4	76.8	41.5	35.3	9.5	89.0	48.0	40.9	11.0
	[75.5,98.6]	[67.4,87.4]	[33.8,50.9]	[29.4,42.5]	[6.0,15.1]	[83.6,92.7]	[41.1,55.1]	[34.2,48.0]	[7.3,16.4]
Madagascar	60.2	45.0	21.7	23.3	15.2	74.8	36.0	38.8	25.2
	[56.1,64.6]	[41.7,48.6]	[19.6,24.0]	[20.7,26.3]	[13.3,17.3]	[72.1,77.3]	[32.9,39.3]	[35.5,42.2]	[22.7,27.9]
Malawi	74.8	57.2	26.5	30.7	17.5	76.5	35.5	41.1	23.5
	[70.8,78.9]	[53.9,60.8]	[23.9,29.4]	[28.7,32.9]	[15.4,20.0]	[73.8,79.0]	[32.6,38.5]	[38.7,43.5]	[21.0,26.2]
Mozambique	108.1	85.4	32.0	53.4	22.7	79.0	29.6	49.4	21.0
	[101.8,114.8]	[80.0,91.1]	[28.8,35.5]	[49.4,57.8]	[19.8,26.0]	[76.5,81.3]	[27.2,32.1]	[46.6,52.2]	[18.7,23.5]
Namibia	58.8	48.4	18.7	29.7	10.5	82.2	31.8	50.4	17.8
	[53.3,64.9]	[43.1,54.3]	[15.3,22.9]	[26.3,33.5]	[8.4,13.1]	[77.9,85.8]	[27.3,36.6]	[45.7,55.2]	[14.2,22.1]
Rwanda	67.1	51.8	22.2	29.5	15.3	77.2	33.1	44.0	22.8
	[62.3,72.2]	[47.8,56.1]	[19.9,24.8]	[26.7,32.7]	[13.5,17.4]	[74.7,79.4]	[30.3,36.1]	[41.3,46.7]	[20.6,25.3]
South Africa	50.6	46.7	24.7	22.0	3.9	92.4	48.8	43.5	7.6
	[43.1,59.4]	[39.3,55.4]	[19.5,31.3]	[17.7,27.4]	[2.8,5.3]	[89.3,94.6]	[41.6,56.1]	[36.7,50.6]	[5.4,10.7]
South Sudan	96.6	78.9	35.9	43.1	17.6	81.8	37.1	44.6	18.2
	[88.5,105.2]	[72.2,86.2]	[31.1,41.4]	[38.0,48.8]	[14.9,20.9]	[79.1,84.1]	[32.7,41.8]	[40.7,48.6]	[15.9,20.9]
Sudan	68.9	59.7	30.4	29.3	9.2	86.7	44.1	42.6	13.3
	[65.3,72.7]	[56.6,63.0]	[27.9,33.1]	[27.0,31.8]	[7.8,10.8]	[84.7,88.4]	[41.0,47.3]	[39.9,45.2]	[11.6,15.3]
Swaziland	79.3	68.9	18.4	50.5	10.4	86.9	23.3	63.6	13.1
	[66.6,94.2]	[57.4,82.6]	[14.0,24.2]	[41.2,61.8]	[7.1,15.2]	[81.7,90.8]	[18.3,29.1]	[58.5,68.5]	[9.2,18.3]
Tanzania	78.1	64.7	29.0	35.7	13.4	82.9	37.2	45.7	17.1
	[72.6,83.9]	[60.1,69.6]	[26.2,32.1]	[32.1,39.6]	[11.3,15.8]	[80.3,85.2]	[34.1,40.4]	[42.5,48.9]	[14.8,19.7]

© 2022 Karlsson O et al. *JAMA Network Open*.

Uganda	73.2	59.0	27.4	31.6	14.2	80.6	37.4	43.2	19.4
	[68.8,77.9]	[55.4,62.9]	[25.3,29.7]	[29.2,34.2]	[12.6,16.0]	[78.6,82.4]	[35.5,39.5]	[40.9,45.4]	[17.6,21.4]
Zambia	64.3	51.4	25.0	26.4	12.9	79.9	38.9	41.1	20.1
	[58.1,71.0]	[46.2,57.1]	[21.8,28.6]	[23.4,29.8]	[11.1,15.0]	[77.5,82.2]	[35.5,42.4]	[38.2,43.9]	[17.8,22.5]
Zimbabwe	72.7	61.2	31.5	29.7	11.5	84.1	43.3	40.9	15.9
	[65.6,80.5]	[55.9,66.8]	[27.6,35.8]	[25.8,34.2]	[8.9,15.0]	[80.8,86.9]	[38.8,47.9]	[36.5,45.4]	[13.1,19.2]
West & Central Africa	116.0	81.9	33.5	48.4	34.0	70.7	28.9	41.7	29.3
	[113.2,118.7]	[80.1,83.8]	[32.4,34.7]	[47.0,49.8]	[32.7,35.4]	[69.9,71.4]	[28.2,29.7]	[40.8,42.6]	[28.6,30.1]
Benin	102.1	73.8	32.5	41.2	28.3	72.3	31.9	40.4	27.7
	[96.2,108.2]	[69.0,78.8]	[29.6,35.8]	[37.8,44.9]	[25.6,31.3]	[69.9,74.5]	[29.5,34.3]	[37.9,42.9]	[25.5,30.1]
Burkina Faso	147.7	105.6	34.3	71.2	42.1	71.5	23.2	48.2	28.5
	[140.2,155.4]	[100.1,111.3]	[31.6,37.3]	[66.9,75.9]	[39.1,45.4]	[70.1,72.9]	[21.8,24.8]	[46.3,50.2]	[27.1,29.9]
Cameroon	89.5	65.1	29.7	35.3	24.5	72.7	33.2	39.5	27.3
	[83.0,96.5]	[60.2,70.3]	[26.5,33.3]	[31.8,39.3]	[21.6,27.7]	[70.2,75.0]	[30.2,36.3]	[36.4,42.7]	[25.0,29.8]
Chad	147.8	107.7	36.2	71.5	40.1	72.9	24.5	48.4	27.1
	[135.5,161.1]	[94.8,122.1]	[32.5,40.4]	[61.7,82.7]	[37.8,42.6]	[69.6,75.9]	[23.1,26.0]	[45.2,51.5]	[24.1,30.4]
Congo	81.8	59.5	24.3	35.2	22.3	72.8	29.8	43.0	27.2
	[74.9,89.2]	[53.6,66.0]	[20.6,28.8]	[31.0,39.9]	[19.3,25.7]	[69.2,76.1]	[25.8,34.1]	[39.3,46.8]	[23.9,30.8]
Congo (DR)	111.6	81.8	30.2	51.7	29.8	73.3	27.0	46.3	26.7
	[105.0,118.5]	[77.0,86.9]	[27.1,33.6]	[47.8,55.8]	[26.7,33.1]	[71.3,75.3]	[24.7,29.5]	[43.6,49.0]	[24.7,28.7]
Cote D'Ivoire	101.1	79.5	35.4	44.0	21.7	78.6	35.0	43.5	21.4
	[94.4,108.3]	[73.9,85.4]	[32.1,39.1]	[39.7,48.8]	[18.6,25.3]	[75.6,81.2]	[32.2,38.0]	[40.3,46.8]	[18.8,24.4]
Gabon	63.3	50.2	25.3	24.9	13.1	79.3	40.0	39.3	20.7
	[56.7,70.6]	[43.8,57.5]	[20.2,31.6]	[20.7,30.0]	[10.5,16.2]	[74.5,83.5]	[33.3,47.1]	[33.4,45.6]	[16.5,25.5]
Gambia	59.2	48.2	29.3	18.9	11.0	81.4	49.5	31.9	18.6
	[54.4,64.3]	[44.3,52.4]	[26.0,33.1]	[17.0,20.9]	[9.1,13.2]	[78.4,84.1]	[46.0,53.1]	[28.7,35.2]	[15.9,21.6]
Ghana	70.1	55.0	30.9	24.1	15.1	78.5	44.0	34.5	21.5
	[63.8,76.8]	[50.3,60.1]	[26.5,35.9]	[20.9,27.8]	[11.8,19.2]	[74.0,82.4]	[39.3,48.9]	[29.5,39.7]	[17.6,26.0]
Guinea	108.5	80.2	33.4	46.9	28.2	74.0	30.8	43.2	26.0
	[99.1,118.6]	[72.8,88.3]	[29.6,37.7]	[41.0,53.5]	[25.0,31.8]	[71.8,76.1]	[27.7,34.0]	[39.8,46.6]	[23.9,28.2]
Liberia	112.9	89.3	33.7	55.5	23.7	79.0	29.9	49.2	21.0
	[102.1,124.7]	[80.6,98.7]	[28.3,40.1]	[50.1,61.5]	[19.9,28.1]	[76.3,81.5]	[26.7,33.2]	[45.0,53.3]	[18.5,23.7]
Mali	112.8	76.3	34.4	41.9	36.5	67.7	30.5	37.2	32.3
	[103.8,122.5]	[69.7,83.6]	[29.8,39.6]	[37.6,46.7]	[32.7,40.7]	[65.2,70.0]	[27.1,34.1]	[34.5,39.9]	[30.0,34.8]
Mauritania	52.7	44.4	27.6	16.9	8.2	84.4	52.3	32.1	15.6
	[47.7,58.1]	[40.0,49.3]	[24.2,31.4]	[14.7,19.4]	[6.8,9.9]	[81.6,86.8]	[48.3,56.3]	[28.6,35.8]	[13.2,18.4]
Niger	153.0	97.5	31.5	66.0	55.6	63.7	20.6	43.1	36.3
	[146.7,159.6]	[92.4,102.8]	[28.8,34.5]	[61.1,71.2]	[51.9,59.5]	[61.6,65.7]	[18.7,22.6]	[40.9,45.3]	[34.3,38.4]
Nigeria	128.8	87.9	36.9	51.0	40.9	68.2	28.6	39.6	31.8
	[122.0,135.9]	[83.3,92.7]	[33.8,40.2]	[48.1,54.1]	[37.8,44.3]	[66.7,69.7]	[26.8,30.5]	[38.0,41.3]	[30.3,33.3]
Sao Tome & Principe	43.0	35.7	18.1	17.6	7.3	83.1	42.1	41.0	16.9
	[35.2,52.5]	[29.0,44.0]	[13.1,25.0]	[13.6,22.8]	[4.7,11.3]	[75.4,88.7]	[33.2,51.4]	[32.0,50.6]	[11.3,24.6]
Senegal	48.4	40.5	23.9	16.6	7.9	83.7	49.4	34.3	16.3
	[43.3,54.2]	[36.0,45.6]	[20.7,27.6]	[14.3,19.4]	[6.0,10.3]	[79.5,87.2]	[44.7,54.1]	[30.7,38.2]	[12.8,20.5]
Sierra Leone	124.3	96.2	29.6	66.6	28.0	77.4	23.8	53.6	22.6
	[117.5,131.4]	[90.5,102.3]	[26.8,32.8]	[61.8,71.7]	[25.1,31.3]	[75.2,79.5]	[21.7,26.1]	[51.2,55.9]	[20.5,24.8]
Togo	74.5	51.0	27.9	23.0	23.5	68.4	37.5	30.9	31.6
	[67.8,81.9]	[45.5,57.1]	[23.7,32.9]	[19.7,26.9]	[19.4,28.5]	[63.3,73.1]	[32.8,42.5]	[26.8,35.4]	[26.9,36.7]
Latin America & Caribbean	32.0	28.6	15.5	13.1	3.4	89.3	48.3	40.9	10.7
	[30.5,33.6]	[27.2,30.0]	[14.6,16.4]	[12.1,14.2]	[3.0,3.9]	[87.9,90.4]	[46.0,50.6]	[38.5,43.4]	[9.6,12.1]
Colombia	18.7	17.4	9.8	7.7	1.3	93.0	52.2	40.8	7.0
	[16.2,21.7]	[15.1,20.2]	[8.1,11.8]	[6.3,9.3]	[0.9,2.0]	[90.0,95.2]	[46.2,58.1]	[35.6,46.3]	[4.8,10.0]
Dominican Republic	35.3	33.0	24.8	8.1	2.3	93.5	70.4	23.1	6.5

© 2022 Karlsson O et al. *JAMA Network Open*.

	[31.6,39.3]	[29.4,37.0]	[21.5,28.6]	[6.4,10.3]	[1.6,3.3]	[90.7,95.4]	[64.4,75.7]	[18.6,28.3]	[4.6,9.3]
El Salvador	19.9	18.2	10.0	8.1	1.8	91.0	50.2	40.8	9.0
	[16.4,24.2]	[14.7,22.4]	[7.5,13.3]	[6.2,10.8]	[0.9,3.5]	[83.0,95.5]	[41.4,58.9]	[31.7,50.6]	[4.5,17.0]
Guatemala	38.5	35.0	17.5	17.4	3.6	90.7	45.4	45.3	9.3
	[35.5,41.8]	[31.9,38.3]	[15.7,19.5]	[15.1,20.1]	[2.8,4.7]	[88.0,92.9]	[41.9,49.1]	[40.8,49.9]	[7.1,12.0]
Guyana	36.5	33.3	21.2	12.2	3.1	91.4	58.1	33.3	8.6
	[29.7,44.8]	[26.5,41.9]	[15.7,28.6]	[8.9,16.5]	[1.5,6.6]	[82.4,96.0]	[47.8,67.7]	[25.1,42.7]	[4.0,17.6]
Haiti	82.9	69.0	31.6	37.4	13.9	83.3	38.1	45.2	16.7
	[76.2,90.1]	[62.4,76.2]	[27.0,36.9]	[32.7,42.8]	[11.7,16.4]	[80.2,85.9]	[33.5,42.9]	[40.7,49.7]	[14.1,19.8]
Honduras	29.5	26.4	16.6	9.8	3.1	89.4	56.2	33.2	10.6
	[27.4,31.8]	[24.3,28.6]	[14.8,18.6]	[8.2,11.7]	[2.4,4.1]	[86.2,92.0]	[50.8,61.4]	[28.2,38.7]	[8.0,13.8]
Paraguay	19.6	18.9	8.2	10.7	0.6	96.8	42.1	54.7	3.2
	[15.7,24.4]	[15.1,23.7]	[6.4,10.6]	[7.5,15.2]	[0.2,1.6]	[92.2,98.7]	[31.9,53.0]	[43.8,65.2]	[1.3,7.8]
Peru	25.2	22.5	12.6	10.0	2.7	89.3	49.8	39.5	10.7
	[22.9,27.8]	[20.3,25.0]	[10.9,14.4]	[8.6,11.6]	[1.9,3.8]	[85.2,92.4]	[45.1,54.6]	[34.9,44.3]	[7.6,14.8]
Suriname	19.9	18.0	13.1	4.8	2.0	90.2	65.8	24.3	9.8
	[14.9,26.6]	[13.4,24.0]	[9.3,18.4]	[3.0,7.9]	[0.9,4.1]	[81.5,95.0]	[52.4,77.1]	[15.6,35.8]	[5.0,18.5]
Low-Income Countries	91.6	70.9	30.7	40.2	20.7	77.4	33.5	43.9	22.6
	[89.6,93.6]	[69.4,72.4]	[29.8,31.6]	[38.9,41.4]	[19.8,21.6]	[76.7,78.1]	[32.6,34.5]	[43.0,44.7]	[21.9,23.3]
Lower-Middle-Income Countries	64.6	53.3	30.1	23.2	11.3	82.5	46.6	35.9	17.5
	[63.2,66.0]	[52.1,54.4]	[29.4,30.8]	[22.4,23.9]	[10.9,11.7]	[82.0,82.9]	[45.7,47.5]	[35.2,36.5]	[17.1,18.0]
Upper-Middle-Income Countries	31.6	28.3	15.9	12.4	3.3	89.6	50.3	39.3	10.4
	[30.1,33.2]	[26.9,29.8]	[14.8,17.1]	[11.6,13.3]	[2.9,3.7]	[88.3,90.7]	[47.9,52.7]	[37.0,41.6]	[9.3,11.7]

Notes: 95% confidence intervals are shown in brackets.

© 2022 Karlsson O et al. *JAMA Network Open*.

eTable 6. Share of Deaths Among Children Younger Than 5 Years Occurring at Different Ages Using Detailed Age Intervals

	<1 month	1–2 months	3–5 months	6–11 months	12–23 months	24–35 months	36–47 months	48–59 months
Pooled	43.0	8.2	7.1	11.5	11.7	9.0	6.0	3.5
	[42.4, 43.7]	[7.9, 8.5]	[6.8, 7.4]	[11.2, 11.9]	[11.4, 12.1]	[8.6, 9.4]	[5.8, 6.2]	[3.3, 3.7]
Least Developed Countries	36.2	8.6	7.8	13.2	13.1	10.2	6.8	4.0
	[35.4, 37.0]	[8.2, 9.0]	[7.4, 8.3]	[12.8, 13.7]	[12.7, 13.6]	[9.8, 10.7]	[6.5, 7.1]	[3.8, 4.2]
East Asia & Pacific	48.5	12.0	8.1	10.4	8.4	5.0	5.1	2.6
	[46.3, 50.7]	[10.7, 13.5]	[7.1, 9.4]	[9.1, 11.8]	[7.3, 9.6]	[4.1, 6.0]	[4.0, 6.4]	[2.0, 3.2]
Cambodia	44.2	18.0	11.2	7.3	7.0	3.1	6.2	3.0
	[40.2, 48.3]	[14.8, 21.8]	[8.5, 14.5]	[5.5, 9.6]	[5.1, 9.5]	[2.1, 4.4]	[4.3, 8.9]	[1.8, 5.0]
Indonesia	50.9	8.6	7.5	11.0	9.5	4.5	5.1	2.8
	[47.8, 54.0]	[6.7, 11.1]	[5.8, 9.6]	[8.9, 13.7]	[8.0, 11.3]	[3.1, 6.3]	[3.7, 6.8]	[2.1, 3.9]
Lao	41.2	26.1	14.8	7.0	2.1	4.1	2.7	1.9
	[37.3, 45.2]	[23.0, 29.5]	[12.4, 17.6]	[5.5, 9.1]	[1.2, 3.6]	[3.2, 5.3]	[1.9, 3.9]	[1.0, 3.3]
Mongolia	43.9	15.1	6.6	12.7	5.3	8.3	7.1	1.2
	[35.3, 52.8]	[9.7, 22.7]	[3.3, 12.7]	[7.9, 19.8]	[3.0, 9.2]	[4.7, 14.1]	[3.4, 14.0]	[0.5, 2.8]
Myanmar	44.4	20.0	7.6	9.3	6.7	4.9	5.9	1.2
	[38.6, 50.4]	[15.9, 24.9]	[5.3, 10.6]	[6.9, 12.5]	[4.8, 9.2]	[3.2, 7.4]	[4.3, 8.1]	[0.8, 2.0]
Papua New Guinea	45.7	6.3	9.5	11.2	8.1	9.8	5.9	3.5
	[39.7, 51.7]	[4.1, 9.6]	[5.4, 16.1]	[9.0, 14.0]	[5.5, 11.7]	[7.4, 12.9]	[4.3, 8.2]	[2.1, 5.8]
Philippines	49.4	9.8	8.6	10.7	8.7	6.0	3.8	3.0
	[44.1, 54.6]	[7.1, 13.4]	[6.1, 12.1]	[8.0, 14.1]	[6.4, 11.8]	[4.4, 8.1]	[2.5, 5.8]	[1.7, 5.3]
Timor-Leste	41.6	10.1	8.0	14.3	6.2	8.3	6.9	4.6
	[36.2, 47.2]	[7.5, 13.3]	[5.9, 10.7]	[11.2, 18.2]	[4.3, 9.0]	[6.3, 11.1]	[4.6, 10.2]	[2.9, 7.2]
Tonga	45.6	10.3	8.5	27.3	1.4	2.4	4.3	NA
	[31.0, 60.9]	[1.8, 42.5]	[3.4, 19.9]	[11.6, 51.8]	[0.1, 13.5]	[0.2, 24.9]	[0.7, 23.9]	
South Asia	57.7	9.1	6.5	8.5	7.6	4.4	3.8	2.4
	[56.7, 58.8]	[8.5, 9.6]	[6.0, 7.0]	[7.9, 9.1]	[7.1, 8.2]	[4.1, 4.8]	[3.5, 4.2]	[2.1, 2.6]
Afghanistan	40.1	13.0	11.6	15.2	9.5	5.9	3.1	1.5
	[37.1, 43.1]	[11.1, 15.2]	[10.0, 13.3]	[12.9, 17.9]	[8.1, 11.3]	[4.7, 7.4]	[2.3, 4.3]	[1.1, 2.2]
Bangladesh	64.0	7.2	5.6	6.5	5.8	4.9	3.7	2.5
	[61.1, 66.7]	[6.0, 8.5]	[4.5, 6.9]	[5.4, 7.7]	[4.5, 7.5]	[3.9, 6.1]	[2.8, 4.8]	[1.8, 3.4]
India	58.4	8.5	6.0	8.0	7.9	4.5	4.2	2.5
	[57.5, 59.4]	[8.2, 8.9]	[5.6, 6.4]	[7.6, 8.5]	[7.4, 8.3]	[4.2, 4.8]	[3.9, 4.4]	[2.3, 2.7]
Maldives	58.3	7.4	13.0	6.2	4.7	3.1	3.0	4.3
	[43.7, 71.7]	[3.8, 14.0]	[5.1, 29.2]	[1.7, 20.1]	[2.4, 9.0]	[1.2, 7.8]	[1.0, 8.4]	[1.6, 11.3]
Nepal	53.8	12.7	9.9	9.0	3.7	6.2	2.0	2.8
	[48.1, 59.4]	[9.6, 16.8]	[6.4, 14.8]	[6.0, 13.2]	[2.1, 6.4]	[3.2, 11.7]	[1.0, 3.9]	[1.4, 5.3]
Pakistan	57.1	10.3	7.2	9.1	7.3	3.9	3.1	2.0
	[53.3, 60.8]	[8.5, 12.4]	[5.7, 9.0]	[7.2, 11.3]	[5.7, 9.3]	[2.8, 5.6]	[2.3, 4.3]	[1.4, 2.9]
Europe & Central Asia	49.0	10.6	9.3	17.1	5.5	4.7	2.6	1.3
	[43.2, 54.8]	[7.3, 15.0]	[5.9, 14.4]	[12.4, 23.2]	[3.8, 8.0]	[2.3, 9.2]	[1.6, 4.1]	[0.7, 2.3]
Albania	58.3	2.1	7.7	20.9	8.8	NA	NA	2.2
	[32.7, 80.1]	[0.3, 13.4]	[1.6, 30.4]	[7.2, 47.2]	[2.4, 27.7]			[0.3, 14.1]
Armenia	50.7	12.4	6.3	9.4	12.6	4.4	0.7	3.5
	[31.7, 69.5]	[3.0, 39.4]	[1.6, 22.5]	[2.5, 29.0]	[5.6, 26.1]	[0.8, 20.5]	[0.1, 8.1]	[0.6, 16.9]
Kyrgyz Republic	63.6	8.0	3.2	13.0	4.6	4.5	1.6	1.4
	[51.1, 74.4]	[4.4, 14.2]	[1.1, 8.9]	[6.4, 24.5]	[1.6, 12.8]	[1.8, 10.8]	[0.5, 5.3]	[0.2, 8.2]
Moldova	75.2	11.6	1.1	6.3	NA	4.8	NA	0.9
	[61.1, 85.5]	[4.3, 28.0]	[0.1, 8.6]	[1.4, 23.8]		[1.0, 20.0]		[0.1, 7.9]

© 2022 Karlsson O et al. *JAMA Network Open*.

Tajikistan	44.6	7.3	10.5	19.3	9.0	3.0	3.6	2.6
	[38.1, 51.3]	[5.0, 10.6]	[7.7, 14.2]	[15.2, 24.3]	[6.1, 12.9]	[1.4, 6.0]	[1.8, 7.2]	[1.2, 5.9]
Turkey	46.3	12.4	9.8	18.7	4.8	5.1	2.1	0.8
	[36.4, 56.5]	[7.4, 20.2]	[5.1, 17.9]	[10.8, 30.5]	[2.0, 10.9]	[1.6, 14.6]	[1.0, 4.6]	[0.1, 4.6]
Turkmenistan	54.0	8.8	10.5	10.3	4.2	5.9	4.7	1.7
	[46.7, 61.0]	[5.9, 13.0]	[6.8, 15.9]	[6.7, 15.6]	[2.3, 7.6]	[3.3, 10.3]	[2.3, 9.1]	[0.4, 6.7]
Middle East & North Africa	51.4	10.8	9.7	12.8	6.6	4.4	2.7	1.6
	[49.1, 53.7]	[9.5, 12.3]	[8.4, 11.2]	[11.4, 14.4]	[5.7, 7.7]	[3.5, 5.4]	[2.0, 3.6]	[1.2, 2.2]
Egypt	53.8	12.1	9.5	10.2	5.8	4.2	3.1	1.1
	[50.2, 57.4]	[9.8, 15.0]	[7.8, 11.6]	[8.0, 13.0]	[4.3, 7.8]	[3.1, 5.5]	[2.0, 4.9]	[0.5, 2.5]
Iraq	52.7	8.1	11.3	14.7	3.7	4.1	3.3	2.2
	[48.6, 56.8]	[6.5, 10.1]	[8.2, 15.2]	[11.5, 18.6]	[2.5, 5.2]	[2.8, 5.9]	[1.9, 5.4]	[1.5, 3.3]
Jordan	55.4	7.3	10.4	15.0	4.1	3.9	2.9	1.0
	[46.4, 64.0]	[4.4, 12.0]	[6.9, 15.2]	[9.5, 22.8]	[2.2, 7.5]	[2.3, 6.5]	[1.1, 7.8]	[0.4, 2.7]
State of Palestine	50.2	14.6	9.0	10.4	6.4	3.2	3.0	3.2
	[44.2, 56.1]	[11.0, 19.2]	[6.5, 12.5]	[7.1, 14.9]	[4.2, 9.5]	[1.5, 6.8]	[1.6, 5.6]	[1.7, 6.0]
Tunisia	59.8	13.2	4.2	9.5	5.2	3.6	3.4	1.0
	[47.7, 70.8]	[7.9, 21.2]	[1.4, 12.1]	[5.3, 16.6]	[2.5, 10.8]	[1.4, 9.3]	[1.3, 8.5]	[0.1, 8.1]
Yemen	46.1	10.3	9.5	15.7	9.8	5.0	1.7	2.0
	[43.1, 49.2]	[8.6, 12.2]	[8.0, 11.2]	[13.9, 17.7]	[8.4, 11.5]	[3.6, 6.8]	[1.1, 2.5]	[1.4, 2.7]
Eastern & Southern Africa	39.4	9.0	8.2	13.2	12.3	8.3	6.0	3.7
	[38.0, 40.9]	[8.3, 9.7]	[7.6, 8.7]	[12.5, 13.8]	[11.6, 13.0]	[7.8, 8.8]	[5.5, 6.6]	[3.4, 4.1]
Angola	30.6	10.4	7.1	14.7	12.1	11.7	8.5	4.8
	[27.6, 33.9]	[8.7, 12.4]	[5.7, 8.8]	[12.1, 17.9]	[10.4, 14.0]	[10.2, 13.4]	[6.7, 10.9]	[3.5, 6.4]
Burundi	30.5	9.6	7.2	13.3	15.2	10.1	9.3	4.6
	[27.8, 33.3]	[8.0, 11.6]	[6.2, 8.4]	[11.7, 15.1]	[13.5, 17.1]	[8.7, 11.8]	[7.9, 11.0]	[3.7, 5.8]
Comoros	51.0	14.1	7.4	4.6	6.7	7.1	5.6	3.5
	[43.9, 58.1]	[9.0, 21.2]	[4.7, 11.5]	[2.6, 8.3]	[4.1, 10.9]	[3.8, 12.6]	[3.1, 9.9]	[1.7, 7.0]
Ethiopia	46.7	10.7	7.2	10.4	9.1	6.3	6.0	3.7
	[42.4, 51.0]	[8.6, 13.2]	[5.4, 9.6]	[8.9, 12.2]	[7.0, 11.7]	[5.0, 8.1]	[4.4, 8.1]	[2.5, 5.3]
Kenya	41.0	7.9	10.1	13.7	11.0	6.8	5.9	3.6
	[37.6, 44.4]	[6.5, 9.6]	[8.6, 11.7]	[12.1, 15.6]	[9.2, 13.1]	[5.4, 8.5]	[4.6, 7.4]	[2.6, 4.9]
Lesotho	48.0	8.7	11.0	15.2	6.0	5.1	3.3	2.7
	[41.1, 55.1]	[6.1, 12.3]	[7.7, 15.5]	[11.3, 20.2]	[3.5, 10.1]	[2.8, 9.1]	[1.5, 6.9]	[1.5, 4.9]
Madagascar	36.0	11.3	7.2	13.4	6.9	12.4	6.9	5.9
	[32.9, 39.3]	[9.3, 13.5]	[5.6, 9.1]	[11.6, 15.4]	[5.6, 8.5]	[10.6, 14.4]	[5.3, 9.0]	[4.6, 7.6]
Malawi	35.5	7.5	6.6	12.4	14.6	11.9	6.7	4.8
	[32.6, 38.5]	[6.4, 8.7]	[5.6, 7.7]	[10.9, 14.2]	[12.8, 16.5]	[10.3, 13.7]	[5.7, 7.9]	[3.6, 6.2]
Mozambique	29.6	10.8	9.6	15.7	13.3	9.7	7.8	3.5
	[27.2, 32.1]	[9.1, 12.7]	[8.3, 11.1]	[13.8, 17.8]	[11.2, 15.7]	[8.4, 11.2]	[6.4, 9.5]	[2.9, 4.3]
Namibia	31.8	11.0	11.8	14.2	13.4	6.3	8.5	3.0
	[27.3, 36.6]	[8.6, 14.1]	[9.5, 14.6]	[11.1, 18.1]	[10.4, 17.0]	[4.1, 9.6]	[5.8, 12.4]	[1.5, 5.6]
Rwanda	33.1	8.3	6.7	14.3	14.7	8.6	8.5	5.8
	[30.3, 36.1]	[6.8, 10.0]	[5.3, 8.4]	[12.3, 16.7]	[12.7, 16.9]	[7.1, 10.4]	[6.5, 10.9]	[4.5, 7.5]
South Africa	48.8	6.2	12.6	17.3	7.4	3.2	2.1	2.4
	[41.6, 56.1]	[4.1, 9.3]	[8.3, 18.7]	[12.0, 24.3]	[4.6, 11.6]	[1.9, 5.3]	[1.1, 3.9]	[1.3, 4.3]
South Sudan	37.1	9.9	11.3	11.2	12.2	8.4	5.4	4.5
	[32.7, 41.8]	[8.0, 12.2]	[9.2, 13.7]	[9.6, 13.2]	[10.5, 14.1]	[7.0, 10.0]	[4.2, 6.9]	[3.5, 5.6]
Sudan	44.1	7.8	8.5	12.1	14.2	7.6	3.3	2.4
	[41.0, 47.3]	[6.0, 10.1]	[7.1, 10.1]	[10.1, 14.4]	[12.4, 16.2]	[6.2, 9.3]	[2.5, 4.4]	[1.7, 3.3]
Swaziland	23.3	8.1	16.1	29.8	9.6	6.5	4.2	2.4
	[18.3, 29.1]	[5.5, 11.9]	[11.8, 21.7]	[25.4, 34.6]	[6.4, 14.1]	[3.8, 11.1]	[2.4, 7.2]	[0.9, 5.9]

© 2022 Karlsson O et al. *JAMA Network Open*.

Tanzania	37.2	6.4	8.0	14.3	17.0	7.9	5.8	3.4
	[34.1, 40.4]	[4.6, 8.9]	[6.4, 9.9]	[12.3, 16.6]	[14.5, 19.8]	[6.6, 9.5]	[4.6, 7.2]	[2.5, 4.7]
Uganda	37.4	7.9	7.1	12.9	15.3	10.1	5.8	3.6
	[35.5, 39.5]	[6.6, 9.4]	[6.1, 8.2]	[11.4, 14.6]	[13.8, 16.8]	[8.5, 11.8]	[4.8, 6.9]	[2.9, 4.4]
Zambia	38.9	8.0	6.5	12.6	14.0	9.7	6.0	4.4
	[35.5, 42.4]	[6.4, 10.1]	[4.8, 8.7]	[10.6, 14.9]	[11.3, 17.1]	[8.0, 11.8]	[4.5, 7.8]	[3.1, 6.1]
Zimbabwe	43.3	9.0	7.4	12.8	11.6	7.4	5.6	2.9
	[38.8, 47.9]	[7.1, 11.4]	[5.9, 9.3]	[10.7, 15.2]	[9.3, 14.5]	[5.5, 9.8]	[3.9, 7.9]	[2.1, 4.0]
West & Central Africa	28.9	5.8	6.4	13.2	16.3	15.3	9.0	5.1
	[28.2, 29.7]	[5.5, 6.1]	[6.0, 6.9]	[12.8, 13.7]	[15.7, 16.9]	[14.7, 15.9]	[8.5, 9.5]	[4.7, 5.4]
Benin	31.9	6.3	6.0	14.1	14.0	12.3	10.4	5.1
	[29.5, 34.3]	[5.1, 7.7]	[5.0, 7.2]	[12.7, 15.8]	[12.6, 15.5]	[10.8, 13.8]	[9.2, 11.8]	[4.1, 6.2]
Burkina Faso	23.2	5.9	7.4	16.0	18.9	15.0	8.7	4.8
	[21.8, 24.8]	[5.1, 6.9]	[6.7, 8.2]	[14.6, 17.5]	[17.7, 20.3]	[13.7, 16.4]	[7.9, 9.5]	[4.1, 5.8]
Cameroon	33.2	7.1	6.7	12.3	13.3	12.3	9.6	5.4
	[30.2, 36.3]	[5.6, 8.9]	[5.8, 7.8]	[10.6, 14.3]	[11.3, 15.7]	[10.7, 14.1]	[8.2, 11.2]	[4.0, 7.2]
Chad	24.5	6.8	7.6	16.3	17.6	14.1	8.4	4.6
	[23.1, 26.0]	[5.9, 7.9]	[6.6, 8.8]	[15.3, 17.4]	[15.5, 19.8]	[12.4, 16.0]	[7.1, 10.0]	[3.9, 5.5]
Congo	29.8	6.2	7.2	14.4	15.2	14.0	8.1	5.2
	[25.8, 34.1]	[4.7, 8.1]	[5.3, 9.6]	[12.3, 16.9]	[12.3, 18.6]	[11.3, 17.1]	[5.9, 11.0]	[3.6, 7.3]
Congo (DR)	27.0	5.8	9.1	16.4	15.0	12.5	8.5	5.7
	[24.7, 29.5]	[4.7, 7.2]	[7.9, 10.5]	[15.2, 17.7]	[13.3, 16.9]	[11.1, 14.1]	[7.3, 9.8]	[4.8, 6.7]
Cote D'Ivoire	35.0	8.1	9.4	11.9	14.0	10.7	7.0	3.7
	[32.2, 38.0]	[6.5, 10.1]	[7.7, 11.4]	[10.2, 13.9]	[12.2, 16.1]	[9.1, 12.5]	[5.6, 8.7]	[2.8, 5.0]
Gabon	40.0	3.7	7.4	16.7	11.5	8.3	7.2	5.1
	[33.3, 47.1]	[2.5, 5.4]	[4.9, 11.0]	[12.9, 21.5]	[8.4, 15.6]	[5.7, 12.0]	[4.3, 11.8]	[3.1, 8.3]
Gambia	49.5	7.2	6.5	8.1	10.1	9.0	5.9	3.7
	[46.0, 53.1]	[5.3, 9.6]	[5.1, 8.4]	[6.4, 10.4]	[8.1, 12.4]	[7.3, 11.0]	[4.5, 7.7]	[2.6, 5.2]
Ghana	44.0	6.4	6.7	10.5	10.8	8.9	6.9	5.7
	[39.3, 48.9]	[4.7, 8.7]	[4.7, 9.4]	[8.0, 13.7]	[8.6, 13.5]	[6.9, 11.5]	[4.6, 10.3]	[4.0, 8.0]
Guinea	30.8	7.1	8.1	13.7	14.2	14.8	7.2	4.0
	[27.7, 34.0]	[6.0, 8.4]	[6.4, 10.3]	[12.1, 15.6]	[12.4, 16.2]	[12.9, 17.0]	[5.9, 8.8]	[3.1, 5.2]
Liberia	29.9	7.0	9.3	15.4	17.5	9.6	6.6	4.8
	[26.7, 33.2]	[5.5, 8.7]	[7.7, 11.2]	[12.8, 18.4]	[14.7, 20.8]	[7.6, 12.0]	[5.0, 8.6]	[3.3, 6.9]
Mali	30.5	6.4	5.7	10.9	14.2	16.9	10.4	5.0
	[27.1, 34.1]	[5.1, 8.0]	[4.9, 6.7]	[9.4, 12.6]	[12.5, 16.0]	[14.9, 19.2]	[8.9, 12.2]	[4.2, 6.0]
Mauritania	52.3	12.8	5.0	7.6	6.7	8.6	4.5	2.5
	[48.3, 56.3]	[10.9, 14.8]	[3.6, 6.8]	[5.8, 9.9]	[4.8, 9.4]	[7.0, 10.7]	[2.8, 7.0]	[1.6, 3.9]
Niger	20.6	5.4	4.9	11.5	21.3	19.7	9.9	6.8
	[18.7, 22.6]	[4.5, 6.3]	[4.0, 6.0]	[10.2, 13.0]	[19.8, 22.8]	[18.2, 21.2]	[8.9, 11.0]	[5.8, 7.8]
Nigeria	28.6	4.9	5.0	12.2	17.4	17.4	9.4	5.0
	[26.8, 30.5]	[4.4, 5.5]	[4.5, 5.7]	[11.3, 13.2]	[16.3, 18.6]	[16.2, 18.7]	[8.6, 10.3]	[4.3, 5.7]
Sao Tome & Principe	42.1	6.4	9.1	16.5	9.0	8.3	3.8	4.8
	[33.2, 51.4]	[3.3, 12.0]	[5.4, 15.1]	[11.2, 23.7]	[4.9, 15.9]	[4.4, 15.1]	[2.0, 6.9]	[1.8, 12.4]
Senegal	49.4	8.7	6.2	10.9	8.5	8.1	5.6	2.6
	[44.7, 54.1]	[6.8, 11.1]	[4.3, 9.0]	[8.3, 14.2]	[6.5, 10.9]	[6.0, 10.9]	[3.9, 7.8]	[1.6, 4.2]
Sierra Leone	23.8	10.0	9.6	18.9	15.1	11.7	6.9	3.9
	[21.7, 26.1]	[8.6, 11.6]	[8.2, 11.2]	[17.3, 20.7]	[13.6, 16.7]	[10.2, 13.5]	[6.0, 8.0]	[3.1, 4.8]
Togo	37.5	3.7	6.7	12.6	7.9	14.2	12.8	4.5
	[32.8, 42.5]	[2.4, 5.6]	[4.8, 9.2]	[9.8, 16.1]	[5.4, 11.2]	[11.4, 17.6]	[10.4, 15.7]	[3.1, 6.5]
Latin America & Caribbean	48.3	10.2	8.3	11.7	10.7	5.2	3.5	2.0
	[46.0, 50.6]	[9.2, 11.3]	[7.0, 9.8]	[10.4, 13.2]	[9.6, 11.9]	[4.5, 6.1]	[2.8, 4.5]	[1.5, 2.5]

© 2022 Karlsson O et al. *JAMA Network Open*.

Colombia	52.2	11.4	10.2	10.6	8.6	3.7	1.0	2.3
	[46.2, 58.1]	[8.0, 16.0]	[7.0, 14.8]	[7.5, 14.8]	[6.3, 11.5]	[2.3, 5.9]	[0.5, 2.1]	[1.3, 4.0]
Dominican Republic	70.4	6.7	4.0	6.6	5.8	2.6	1.7	2.3
	[64.4, 75.7]	[5.0, 8.9]	[2.5, 6.5]	[4.8, 9.1]	[3.6, 9.0]	[1.5, 4.2]	[0.9, 3.2]	[1.1, 4.7]
El Salvador	50.2	12.3	11.5	10.5	6.6	5.8	0.8	2.4
	[41.4, 58.9]	[7.0, 20.5]	[7.3, 17.5]	[6.6, 16.3]	[3.2, 13.1]	[2.5, 12.7]	[0.2, 2.5]	[0.7, 7.9]
Guatemala	45.4	12.7	9.2	10.6	12.9	5.1	3.2	1.0
	[41.9, 49.1]	[9.7, 16.5]	[7.0, 12.0]	[8.3, 13.3]	[10.3, 16.0]	[3.5, 7.3]	[2.2, 4.7]	[0.5, 2.2]
Guyana	58.1	6.6	8.8	11.8	6.1	1.4	5.3	1.9
	[47.8, 67.7]	[3.9, 11.0]	[5.0, 15.1]	[7.5, 18.1]	[2.6, 13.4]	[0.4, 4.6]	[2.0, 13.2]	[0.4, 8.7]
Haiti	38.1	8.5	8.8	14.9	13.0	7.7	6.6	2.5
	[33.5, 42.9]	[6.9, 10.3]	[6.8, 11.4]	[12.8, 17.1]	[11.2, 15.0]	[6.1, 9.7]	[4.8, 9.1]	[1.6, 3.8]
Honduras	56.2	7.7	6.2	10.3	9.1	4.9	4.2	1.5
	[50.8, 61.4]	[5.7, 10.3]	[4.3, 8.8]	[7.5, 14.0]	[6.7, 12.1]	[3.3, 7.2]	[2.6, 6.5]	[0.8, 2.7]
Paraguay	42.1	15.8	14.6	18.4	5.9	1.8	1.1	0.3
	[31.9, 53.0]	[8.1, 28.5]	[8.1, 24.8]	[9.0, 33.9]	[2.2, 14.9]	[0.6, 5.5]	[0.2, 5.6]	[0.0, 4.0]
Peru	49.8	10.7	5.9	11.1	11.8	5.2	3.2	2.3
	[45.1, 54.6]	[7.7, 14.7]	[4.0, 8.6]	[7.4, 16.2]	[8.5, 16.1]	[3.1, 8.6]	[1.4, 7.0]	[1.4, 3.8]
Suriname	65.8	6.7	6.9	9.4	1.2	5.7	4.1	NA
	[52.4, 77.1]	[3.2, 13.7]	[2.9, 15.6]	[4.2, 20.0]	[0.4, 3.8]	[2.3, 13.7]	[1.5, 10.5]	
Low-Income Countries	33.5	8.1	8.0	13.8	13.9	11.1	7.1	4.3
	[32.6, 34.5]	[7.6, 8.7]	[7.6, 8.4]	[13.2, 14.5]	[13.3, 14.5]	[10.7, 11.6]	[6.8, 7.5]	[4.0, 4.6]
Lower-Middle-Income Countries	46.6	8.1	6.5	10.3	11.0	8.5	5.8	3.2
	[45.7, 47.5]	[7.8, 8.4]	[6.2, 6.8]	[9.8, 10.8]	[10.5, 11.5]	[8.2, 8.9]	[5.5, 6.0]	[3.1, 3.4]
Upper-Middle-Income Countries	50.3	8.8	9.1	13.0	8.4	4.3	3.8	2.4
	[47.9, 52.7]	[7.8, 10.0]	[8.0, 10.3]	[11.4, 14.9]	[7.3, 9.6]	[3.5, 5.2]	[3.0, 4.7]	[1.9, 3.0]

Notes: 95% confidence intervals are shown in brackets.

© 2022 Karlsson O et al. *JAMA Network Open*.

eTable 7. Mortality (Deaths per 1000) at Different Ages by Living Standards

	0–59 months			0–23 months			Neonates			1–23 months			24–59 months		
	Worst	Best	Difference	Worst	Best	Difference	Worst	Best	Difference	Worst	Best	Difference	Worst	Best	Difference
Pooled	84.5	40.0	44.5	67.2	35.1	32.1	33.7	21.1	12.5	33.5	13.9	19.5	17.3	4.9	12.4
	[82.3,86.8]	[38.3,41.8]	[41.8,47.1]	[65.4,68.9]	[33.5,36.8]	[29.9,34.3]	[32.6,34.8]	[20.0,22.4]	[11.0,14.1]	[32.1,34.9]	[13.1,14.8]	[17.8,21.2]	[16.4,18.3]	[4.5,5.4]	[11.7,13.2]
Least Developed Countries	92.9	56.8	36.1	72.1	47.9	24.2	30.4	25.1	5.3	41.7	22.8	18.9	20.9	8.9	11.9
	[89.8,96.1]	[54.5,59.2]	[31.5,40.7]	[69.4,74.8]	[45.6,50.3]	[19.8,28.5]	[28.8,32.1]	[23.0,27.4]	[2.9,7.7]	[39.8,43.6]	[21.1,24.7]	[15.6,22.1]	[19.9,21.9]	[8.1,9.9]	[10.5,13.4]
East Asia & Pacific	57.0	21.4	35.6	48.7	19.4	29.3	23.1	12.1	11.0	25.6	7.3	18.4	8.3	2.0	6.3
	[53.5,60.8]	[18.0,25.5]	[29.7,41.5]	[45.6,52.1]	[16.1,23.3]	[24.3,34.4]	[20.7,25.8]	[9.7,15.1]	[7.1,14.9]	[23.1,28.5]	[5.5,9.6]	[14.9,21.8]	[7.0,9.9]	[1.2,3.4]	[4.4,8.2]
Cambodia	76.4	18.7	57.7	67.3	16.8	50.5	26.8	11.7	15.1	40.5	5.1	35.4	9.1	1.9	7.2
	[63.9,91.1]	[13.5,25.9]	[41.6,73.8]	[55.8,81.1]	[11.6,24.3]	[35.5,65.5]	[19.7,36.4]	[7.9,17.5]	[5.4,24.7]	[32.0,51.1]	[2.6,10.1]	[24.7,46.1]	[5.4,15.3]	[0.7,4.8]	[2.7,11.7]
Indonesia	52.5	23.9	28.6	45.3	21.5	23.8	22.6	12.6	10.0	22.7	8.8	13.9	7.2	2.4	4.7
	[47.5,58.0]	[20.0,28.5]	[19.6,37.6]	[41.2,49.8]	[17.7,26.0]	[15.6,32.1]	[19.3,26.4]	[9.9,16.1]	[4.1,15.8]	[19.4,26.6]	[6.5,12.1]	[8.7,19.0]	[4.8,10.7]	[1.4,4.2]	[1.5,7.9]
Lao	67.0	20.5	46.5	61.1	17.8	43.2	23.6	14.0	9.6	37.4	3.8	33.6	6.0	2.7	3.3
	[59.4,75.6]	[13.5,31.0]	[34.8,58.2]	[54.0,69.0]	[11.1,28.5]	[32.0,54.4]	[20.0,28.0]	[8.5,23.0]	[2.5,16.8]	[31.9,43.9]	[1.7,8.7]	[25.9,41.3]	[4.3,8.3]	[1.2,6.0]	[-0.3,6.9]
Mongolia	29.8	14.2	15.6	25.4	10.8	14.5	13.6	6.4	7.1	11.8	4.4	7.4	4.4	3.4	1.0
	[24.9,35.6]	[7.4,27.2]	[4.8,26.4]	[20.3,31.6]	[4.5,25.6]	[3.6,25.4]	[9.6,19.0]	[2.3,17.7]	[0.4,13.9]	[8.4,16.6]	[1.7,11.1]	[1.3,13.5]	[2.0,9.5]	[1.1,9.9]	[-4.3,6.4]
Myanmar	98.8	25.9	72.9	84.7	24.0	60.7	35.0	17.1	17.9	49.6	6.9	42.8	14.2	2.0	12.2
	[83.9,116.0]	[16.3,41.1]	[51.0,94.7]	[69.9,102.2]	[14.8,38.6]	[40.1,81.2]	[27.7,44.2]	[9.8,29.7]	[4.8,31.0]	[39.3,62.5]	[2.9,16.2]	[27.4,58.2]	[9.7,20.5]	[0.4,9.8]	[6.8,17.6]
Papua New Guinea	69.2	35.9	33.2	54.6	31.0	23.7	28.7	19.7	9.0	26.0	11.3	14.7	14.5	5.0	9.5
	[54.8,86.9]	[27.4,47.0]	[11.7,54.7]	[42.1,70.6]	[23.8,40.2]	[4.3,43.1]	[22.0,37.2]	[14.7,26.4]	[-1.6,19.5]	[17.8,37.7]	[8.2,15.4]	[1.2,28.2]	[9.7,21.5]	[2.5,9.7]	[3.1,15.9]
Philippines	42.0	11.2	30.8	34.9	10.7	24.2	17.9	7.6	10.3	17.0	3.1	13.9	7.1	0.5	6.6
	[34.9,50.4]	[6.9,18.0]	[21.4,40.3]	[28.7,42.5]	[6.6,17.3]	[15.4,33.0]	[13.8,23.1]	[4.1,13.9]	[3.8,16.9]	[13.3,21.6]	[1.4,7.0]	[8.8,19.0]	[4.8,10.4]	[0.1,3.1]	[3.8,9.5]
Timor-Leste	54.8	25.1	29.7	40.3	20.4	20.0	16.8	16.6	0.2	23.5	3.8	19.7	14.4	4.7	9.7
	[45.4,66.0]	[16.7,37.6]	[17.8,41.5]	[32.5,49.9]	[13.4,30.9]	[9.4,30.5]	[12.0,23.5]	[10.3,26.5]	[-9.0,9.5]	[18.9,29.2]	[2.0,7.3]	[14.8,24.6]	[10.0,20.9]	[2.2,10.1]	[4.4,15.0]
Tonga	15.7	1.1	14.6	15.7	1.1	14.6	5.9	NA	NA	9.8	1.1	8.8	NA	NA	NA
	[7.1,34.3]	[0.2,4.7]	[3.2,26.1]	[7.1,34.3]	[0.2,4.7]	[3.2,26.1]	[2.8,12.4]			[2.8,33.6]	[0.2,4.7]	[-2.4,19.9]			
South Asia	76.4	30.2	46.2	66.4	27.8	38.6	41.5	19.9	21.6	24.9	7.9	17.0	10.0	2.4	7.6
	[74.3,78.5]	[27.7,32.9]	[42.9,49.5]	[64.5,68.3]	[25.2,30.6]	[35.8,41.4]	[40.1,43.0]	[17.8,22.2]	[19.2,24.1]	[23.6,26.3]	[6.9,9.0]	[14.9,19.1]	[9.2,10.9]	[1.8,3.2]	[6.5,8.7]
Afghanistan	80.5	39.9	40.6	70.9	36.4	34.4	32.1	17.3	14.9	38.7	19.2	19.6	9.7	3.5	6.2
	[73.7,87.9]	[33.6,47.5]	[26.4,54.8]	[64.3,78.0]	[30.3,43.8]	[21.2,47.6]	[26.5,38.8]	[12.3,24.1]	[3.4,26.4]	[34.0,44.1]	[14.2,25.8]	[9.4,29.7]	[7.3,12.7]	[2.2,5.6]	[3.3,9.0]
Bangladesh	50.6	28.2	22.3	44.5	25.7	18.8	30.2	18.8	11.4	14.3	6.9	7.4	6.1	2.5	3.6
	[45.8,55.8]	[24.0,33.2]	[16.3,28.3]	[39.8,49.7]	[21.5,30.7]	[12.7,24.8]	[26.7,34.2]	[15.0,23.7]	[6.1,16.6]	[11.7,17.5]	[4.9,9.6]	[4.3,10.5]	[4.8,7.6]	[1.7,3.8]	[1.8,5.4]
India	75.2	24.5	50.7	65.3	22.4	42.9	41.6	15.4	26.2	23.7	7.0	16.7	9.9	2.1	7.8
	[73.2,77.3]	[22.8,26.3]	[48.2,53.2]	[63.4,67.3]	[20.8,24.1]	[40.7,45.1]	[39.8,43.4]	[14.3,16.6]	[24.5,27.8]	[22.7,24.7]	[5.9,8.2]	[15.6,17.9]	[9.4,10.5]	[1.6,2.8]	[6.7,8.9]
Maldives	15.0	22.6	-7.6	11.7	20.6	-9.0	8.0	20.6	-12.7	3.7	NA	NA	3.3	2.0	1.3
	[9.9,22.7]	[10.4,48.4]	[-28.6,13.3]	[7.5,18.2]	[9.2,45.8]	[-29.5,11.5]	[4.8,13.1]	[9.2,45.8]	[-32.3,7.0]	[1.8,7.6]			[1.6,6.8]	[0.3,12.0]	[-3.6,6.3]
Nepal	39.7	19.7	20.1	35.4	15.1	20.3	22.0	12.8	9.2	13.4	2.3	11.1	4.3	4.5	-0.2
	[31.9,49.3]	[12.6,30.6]	[9.4,30.7]	[27.7,45.1]	[9.1,25.1]	[10.1,30.5]	[15.2,31.6]	[7.3,22.3]	[-0.2,18.5]	[10.0,18.1]	[0.7,8.0]	[6.6,15.6]	[2.4,7.7]	[0.9,22.3]	[-6.2,5.8]
Pakistan	97.5	54.0	43.5	84.2	50.8	33.5	49.9	40.5	9.4	34.3	10.3	24.0	13.2	3.2	10.0
	[85.2,111.3]	[42.2,68.8]	[28.2,58.7]	[72.8,97.4]	[40.0,64.2]	[18.5,48.4]	[42.7,58.3]	[30.3,53.8]	[-0.4,19.3]	[25.7,45.7]	[6.7,15.8]	[13.4,34.7]	[10.3,16.9]	[1.2,8.3]	[5.8,14.2]
Europe & Central Asia	30.2	11.1	19.1	27.7	10.6	17.1	13.1	5.1	8.0	14.5	5.5	9.0	2.5	0.5	2.0
	[25.7,35.4]	[7.3,16.9]	[11.4,26.8]	[23.5,32.6]	[6.8,16.5]	[9.4,24.7]	[10.2,16.8]	[3.2,8.2]	[3.8,12.2]	[11.5,18.3]	[2.4,12.6]	[3.6,14.5]	[1.2,5.2]	[0.2,1.3]	[0.3,3.7]
Albania	14.3	0.4	13.9	13.8	0.4	13.4	5.2	0.4	4.8	8.6	NA	NA	0.5	NA	NA
	[8.1,25.3]	[0.1,2.0]	[6.8,21.1]	[7.7,24.7]	[0.1,2.0]	[6.4,20.4]	[2.6,10.6]	[0.1,2.0]	[1.1,8.6]	[3.5,20.7]			[0.1,3.5]		
Armenia	14.6	4.5	10.1	14.6	4.5	10.1	4.0	2.2	1.9	10.6	2.4	8.3	NA	NA	NA
	[7.8,27.3]	[1.6,12.7]	[-0.3,20.5]	[7.8,27.3]	[1.6,12.7]	[-0.3,20.5]	[1.3,12.8]	[0.5,9.0]	[-2.9,6.7]	[5.2,21.6]	[0.4,13.8]	[-1.3,17.8]			

© 2022 Karlsson O et al. *JAMA Network Open*.

Kyrgyz Republic	21.3	11.0	10.4	21.1	10.5	10.7	12.8	7.9	4.8	8.4	2.5	5.9	0.2	0.5	-0.3
	[14.7,30.9]	[6.1,19.7]	[-1.0,21.7]	[14.6,30.6]	[5.7,19.2]	[-0.5,21.9]	[7.6,21.3]	[4.0,15.9]	[-6.1,15.8]	[4.2,16.7]	[1.1,5.6]	[-1.4,13.2]	[0.0,1.0]	[0.1,3.1]	[-1.4,0.7]
Moldova	29.8	12.5	17.3	29.8	12.5	17.3	22.5	10.2	12.2	7.4	2.3	5.1	NA	NA	NA
	[16.8,52.5]	[7.8,19.9]	[0.0,34.6]	[16.8,52.5]	[7.8,19.9]	[0.0,34.6]	[11.5,43.6]	[5.7,18.3]	[-2.7,27.2]	[2.2,24.2]	[0.5,11.5]	[-3.6,13.7]			
Tajikistan	46.7	20.4	26.2	45.1	19.6	25.5	19.6	12.0	7.6	25.4	7.5	17.9	1.6	0.9	0.7
	[37.7,57.7]	[16.0,26.1]	[13.2,39.3]	[36.2,56.0]	[15.3,24.9]	[12.9,38.2]	[14.3,27.0]	[8.3,17.3]	[-1.2,16.4]	[19.0,34.0]	[4.9,11.5]	[8.8,27.0]	[0.5,4.7]	[0.3,2.9]	[-1.3,2.6]
Turkey	27.9	8.1	19.8	25.0	7.8	17.3	11.2	1.8	9.3	13.9	5.9	7.9	2.8	0.3	2.5
	[21.0,36.9]	[2.9,22.4]	[8.5,31.1]	[18.6,33.7]	[2.7,22.4]	[6.0,28.6]	[7.5,16.6]	[0.2,13.9]	[3.5,15.2]	[9.0,21.3]	[1.9,18.0]	[-2.0,17.8]	[1.2,6.6]	[0.0,2.4]	[0.4,4.6]
Turkmenistan	39.5	23.1	16.5	34.1	21.3	12.8	21.1	16.4	4.8	13.0	5.0	8.0	5.4	1.7	3.7
	[30.1,51.7]	[16.4,32.4]	[-1.4,34.3]	[25.7,45.1]	[15.0,30.3]	[-3.2,28.7]	[14.4,30.9]	[10.6,25.1]	[-7.8,17.3]	[8.3,20.2]	[2.5,9.8]	[-0.1,16.1]	[2.6,11.1]	[0.4,6.7]	[-1.0,8.4]
Middle East & North Africa	41.4	20.9	20.5	37.3	19.5	17.8	20.7	11.8	9.0	16.6	7.8	8.8	4.1	1.3	2.7
	[37.9,45.2]	[17.9,24.3]	[15.0,26.0]	[33.8,41.2]	[16.7,22.9]	[12.5,23.0]	[18.4,23.3]	[9.7,14.3]	[5.8,12.1]	[14.4,19.1]	[6.2,9.7]	[5.5,12.2]	[3.3,5.1]	[0.8,2.4]	[1.7,3.8]
Egypt	42.2	19.2	22.9	38.6	18.6	19.9	23.1	11.1	11.9	15.5	7.5	8.0	3.6	0.6	3.0
	[35.0,50.8]	[15.4,24.0]	[14.4,31.5]	[31.7,46.9]	[15.0,23.2]	[11.3,28.6]	[18.0,29.5]	[8.4,14.7]	[5.6,18.2]	[12.2,19.7]	[5.5,10.3]	[2.9,13.1]	[2.1,6.2]	[0.1,2.8]	[0.8,5.2]
Iraq	29.3	16.3	13.0	25.5	14.3	11.3	15.4	8.0	7.4	10.2	6.3	3.9	3.8	2.0	1.7
	[25.2,34.1]	[11.8,22.5]	[6.5,19.5]	[21.6,30.2]	[10.2,19.9]	[4.9,17.6]	[12.5,18.8]	[5.7,11.2]	[2.9,11.8]	[8.1,12.7]	[3.7,10.7]	[0.2,7.5]	[3.0,4.8]	[0.9,4.5]	[-0.3,3.8]
Jordan	19.4	16.3	3.1	18.0	16.2	1.8	10.0	10.9	-0.9	8.0	5.3	2.7	1.4	0.1	1.3
	[14.6,25.6]	[8.8,30.0]	[-7.5,13.6]	[13.4,24.2]	[8.7,29.9]	[-8.9,12.5]	[7.2,13.9]	[5.4,22.0]	[-9.2,7.5]	[4.5,14.2]	[1.9,14.3]	[-2.5,7.8]	[0.7,2.6]	[0.0,0.7]	[0.4,2.2]
State of Palestine	24.7	16.4	8.3	23.6	14.2	9.3	10.8	6.4	4.4	12.8	7.8	5.0	1.1	2.1	-1.0
	[18.7,32.4]	[11.7,22.9]	[-0.3,16.9]	[17.8,31.1]	[10.3,19.8]	[0.7,18.0]	[7.8,14.9]	[3.8,10.9]	[-0.6,9.3]	[8.4,19.4]	[5.4,11.2]	[-1.5,11.5]	[0.4,2.9]	[0.8,5.3]	[-3.1,1.1]
Tunisia	25.9	5.4	20.5	22.8	4.8	18.1	15.2	3.1	12.1	7.6	1.7	5.9	3.1	0.7	2.4
	[18.2,36.7]	[2.6,11.3]	[10.6,30.3]	[15.8,32.8]	[2.1,10.7]	[8.5,27.6]	[9.3,24.8]	[1.1,8.9]	[3.9,20.3]	[4.0,14.3]	[0.5,5.8]	[1.4,10.5]	[1.0,9.5]	[0.1,3.9]	[-0.6,5.4]
Yemen	69.1	37.8	31.3	61.9	35.1	26.8	28.5	22.4	6.2	33.4	12.7	20.7	7.2	2.8	4.5
	[61.5,77.7]	[32.2,44.4]	[21.1,41.6]	[55.0,69.6]	[29.7,41.4]	[16.9,36.8]	[24.3,33.5]	[18.1,27.5]	[-1.3,13.6]	[27.4,40.6]	[8.4,19.2]	[13.4,27.9]	[5.4,9.7]	[1.5,5.1]	[2.2,6.8]
Eastern & Southern Africa	84.4	57.3	27.1	66.5	50.5	16.0	29.0	28.8	0.2	37.5	21.6	15.9	17.9	6.8	11.1
	[81.0,88.0]	[53.7,61.1]	[20.5,33.7]	[63.4,69.8]	[47.0,54.2]	[10.0,22.1]	[26.6,31.5]	[25.6,32.4]	[-4.1,4.4]	[35.0,40.2]	[19.2,24.4]	[12.1,19.6]	[16.5,19.5]	[5.8,8.1]	[8.9,13.3]
Angola	102.2	38.8	63.3	74.2	28.8	45.4	31.1	11.5	19.6	43.1	17.3	25.8	28.0	10.0	18.0
	[92.1,113.2]	[27.4,54.7]	[42.1,84.6]	[66.5,82.7]	[19.9,41.6]	[27.6,63.2]	[24.8,38.9]	[7.1,18.7]	[8.9,30.2]	[35.9,51.6]	[10.7,27.7]	[13.6,38.1]	[23.1,33.9]	[6.0,16.8]	[10.3,25.6]
Burundi	119.0	51.6	67.4	89.5	42.7	46.8	33.8	21.0	12.8	55.7	21.8	34.0	29.5	8.9	20.6
	[107.1,132.0]	[42.0,63.3]	[54.0,80.8]	[80.7,99.2]	[33.7,54.1]	[34.2,59.4]	[27.1,42.1]	[15.7,28.0]	[3.4,22.3]	[49.6,62.6]	[17.0,27.9]	[26.5,41.4]	[24.0,36.2]	[6.4,12.3]	[14.5,26.7]
Comoros	52.0	40.1	11.9	39.4	38.3	1.1	18.9	22.7	-3.8	20.5	15.6	4.9	12.6	1.8	10.8
	[38.7,69.4]	[26.4,60.4]	[-13.3,37.1]	[27.6,56.0]	[25.0,58.1]	[-26.6,28.8]	[11.1,32.2]	[12.1,42.2]	[-23.1,15.5]	[11.8,35.2]	[7.8,31.0]	[-12.5,22.3]	[6.1,25.6]	[0.3,10.8]	[1.0,20.5]
Ethiopia	89.7	66.7	23.0	71.5	60.8	10.7	36.3	40.0	-3.8	35.2	20.7	14.5	18.3	5.9	12.3
	[74.0,108.4]	[53.3,83.2]	[2.0,44.0]	[58.7,86.8]	[47.1,78.1]	[-9.5,31.0]	[28.9,45.5]	[27.2,58.5]	[-20.8,13.3]	[25.1,49.1]	[14.0,30.6]	[3.7,25.3]	[13.4,24.8]	[2.1,16.5]	[4.7,19.9]
Kenya	56.7	46.6	10.1	45.8	41.9	3.9	20.1	25.6	-5.5	25.8	16.3	9.4	10.9	4.7	6.2
	[51.4,62.5]	[39.2,55.3]	[0.3,19.9]	[41.4,50.8]	[34.7,50.5]	[-6.6,14.5]	[17.1,23.6]	[20.1,32.5]	[-13.0,1.9]	[22.6,29.3]	[11.8,22.5]	[3.5,15.4]	[8.7,13.6]	[2.4,9.0]	[2.8,9.6]
Lesotho	83.3	80.3	3.0	76.2	65.1	11.1	35.9	38.4	-2.5	40.3	26.7	13.6	7.1	15.2	-8.1
	[69.9,99.1]	[57.3,111.3]	[-27.8,33.8]	[63.2,91.6]	[43.6,96.0]	[-21.4,43.7]	[26.6,48.4]	[22.3,65.4]	[-29.7,24.8]	[32.1,50.4]	[16.3,43.4]	[-4.2,31.3]	[4.0,12.7]	[6.8,33.5]	[-19.6,3.4]
Madagascar	74.6	39.1	35.6	51.3	33.0	18.3	22.6	19.1	3.5	28.7	14.0	14.8	23.3	6.0	17.3
	[63.8,87.1]	[31.5,48.4]	[23.2,47.9]	[43.5,60.5]	[25.6,42.5]	[7.7,28.9]	[17.7,28.9]	[13.9,26.1]	[-3.2,10.3]	[24.0,34.3]	[9.2,21.3]	[7.4,22.1]	[17.5,30.9]	[3.4,10.7]	[10.1,24.4]
Malawi	83.2	60.1	23.0	61.0	49.2	11.9	27.5	25.3	2.2	33.5	23.8	9.7	22.1	10.9	11.2
	[74.6,92.6]	[50.6,71.3]	[7.1,39.0]	[53.4,69.7]	[41.1,58.8]	[-1.2,24.9]	[23.0,32.8]	[19.6,32.6]	[-5.5,9.8]	[28.3,39.8]	[18.7,30.3]	[1.9,17.5]	[18.4,26.6]	[7.1,16.8]	[5.2,17.2]
Mozambique	129.4	90.7	38.7	98.2	77.4	20.8	33.1	32.7	0.5	65.0	44.7	20.3	31.2	13.3	17.9
	[113.3,147.5]	[80.4,102.2]	[16.6,60.8]	[86.0,111.8]	[67.6,88.4]	[1.3,40.3]	[25.9,42.3]	[27.1,39.4]	[-8.3,9.3]	[55.6,76.0]	[37.0,54.0]	[4.3,36.3]	[23.5,41.3]	[9.9,17.9]	[10.4,25.4]
Namibia	66.8	30.7	36.1	58.1	26.2	31.9	23.1	11.4	11.6	35.0	14.8	20.2	8.7	4.5	4.3
	[58.7,75.9]	[19.1,48.8]	[16.5,55.7]	[49.8,67.6]	[15.5,43.9]	[13.7,50.0]	[17.1,31.0]	[6.5,19.9]	[-0.5,23.7]	[28.2,43.3]	[7.6,28.5]	[9.2,31.3]	[5.6,13.6]	[1.5,13.0]	[-1.5,10.0]
Rwanda	84.4	40.0	44.4	63.6	31.8	31.8	23.5	13.2	10.3	40.1	18.6	21.5	20.8	8.2	12.6
	[74.1,96.1]	[31.8,50.2]	[31.1,57.7]	[55.2,73.1]	[25.4,39.8]	[21.1,42.5]	[18.7,29.4]	[9.3,18.8]	[4.1,16.4]	[33.3,48.2]	[13.3,25.9]	[11.7,31.3]	[15.6,27.7]	[5.3,12.7]	[6.1,19.1]

© 2022 Karlsson O et al. *JAMA Network Open*.

South Africa	67.2	41.3	25.8	58.9	39.8	19.1	25.3	29.4	-4.1	33.6	10.4	23.2	8.3	1.5	6.8
	[53.1,84.6]	[19.0,87.6]	[-11.6,63.3]	[46.2,74.8]	[18.0,85.8]	[-18.3,56.4]	[18.0,35.5]	[10.3,81.3]	[-32.4,24.2]	[25.5,44.0]	[3.3,32.5]	[5.8,40.5]	[4.4,15.6]	[0.3,7.9]	[2.1,11.4]
South Sudan	94.2	105.0	-10.8	76.8	87.8	-11.0	34.7	38.6	-3.9	42.1	49.2	-7.1	17.4	17.2	0.2
	[80.7,109.8]	[92.7,118.8]	[-28.4,6.8]	[65.8,89.6]	[75.7,101.7]	[-30.5,8.5]	[27.0,44.5]	[31.4,47.4]	[-15.6,7.9]	[34.4,51.5]	[40.3,60.0]	[-21.8,7.6]	[12.7,23.8]	[12.1,24.3]	[-8.9,9.3]
Sudan	80.7	42.6	38.0	70.3	38.0	32.4	36.7	25.7	11.0	33.7	12.2	21.4	10.4	4.7	5.7
	[71.6,90.8]	[36.3,50.0]	[26.6,49.5]	[61.9,79.8]	[32.0,44.9]	[21.5,43.2]	[29.4,45.7]	[19.6,33.7]	[2.8,19.1]	[28.6,39.6]	[8.5,17.6]	[14.0,28.8]	[7.9,13.5]	[2.8,7.9]	[2.0,9.4]
Swaziland	102.6	50.8	51.9	94.7	35.1	59.6	27.1	9.3	17.8	67.6	25.8	41.8	8.0	15.7	-7.7
	[83.7,125.2]	[31.5,80.9]	[19.6,84.1]	[77.0,115.9]	[22.6,54.3]	[33.0,86.1]	[18.5,39.7]	[3.7,23.3]	[5.1,30.5]	[53.6,84.9]	[15.9,41.5]	[21.4,62.1]	[3.9,16.2]	[4.8,49.7]	[-26.2,10.8]
Tanzania	77.6	72.8	4.9	60.1	67.9	-7.7	20.2	37.3	-17.1	39.9	30.5	9.3	17.5	4.9	12.6
	[66.2,90.8]	[61.9,85.3]	[-12.7,22.4]	[49.6,72.7]	[57.7,79.7]	[-23.2,7.7]	[16.1,25.4]	[30.7,45.3]	[-28.3,-5.9]	[30.6,51.8]	[22.9,40.6]	[-1.4,20.1]	[12.7,24.1]	[2.5,9.6]	[4.4,20.9]
Uganda	88.4	52.6	35.7	69.3	44.5	24.8	27.8	25.7	2.1	41.5	18.7	22.8	19.1	8.2	10.9
	[81.0,96.4]	[44.0,62.8]	[21.7,49.8]	[62.8,76.5]	[36.9,53.5]	[12.7,37.0]	[24.1,32.1]	[19.7,33.6]	[-7.3,11.5]	[36.5,47.2]	[14.7,23.8]	[16.6,28.9]	[15.9,22.8]	[5.1,12.9]	[5.7,16.2]
Zambia	66.6	57.4	9.1	51.3	53.1	-1.8	25.8	25.6	0.2	25.4	27.5	-2.0	15.3	4.4	10.9
	[57.1,77.4]	[47.6,69.1]	[-6.4,24.7]	[43.1,60.9]	[43.5,64.5]	[-15.8,12.2]	[20.9,31.9]	[18.9,34.6]	[-10.9,11.4]	[19.2,33.6]	[20.9,35.9]	[-11.4,7.3]	[11.8,19.8]	[2.3,8.3]	[6.6,15.2]
Zimbabwe	91.1	51.0	40.1	75.2	43.4	31.8	35.3	26.2	9.1	40.0	17.2	22.7	15.8	7.6	8.3
	[69.7,118.2]	[40.3,64.3]	[11.7,68.4]	[59.6,94.5]	[34.8,54.1]	[9.7,53.9]	[27.8,44.7]	[20.2,34.0]	[-4.3,22.5]	[27.9,57.0]	[12.4,23.9]	[5.7,39.7]	[9.2,27.3]	[4.5,12.8]	[-0.9,17.5]
West & Central Africa	142.8	65.4	77.4	96.2	53.1	43.1	34.6	27.1	7.5	61.6	26.0	35.6	46.6	12.3	34.3
	[137.4,148.4]	[61.5,69.6]	[70.9,84.0]	[92.6,99.9]	[49.6,56.8]	[37.9,48.3]	[32.4,37.0]	[24.7,29.7]	[4.5,10.4]	[58.8,64.4]	[23.3,28.9]	[31.2,40.0]	[43.4,50.0]	[11.2,13.6]	[30.9,37.7]
Benin	107.8	60.1	47.7	78.6	46.9	31.7	29.9	24.2	5.8	48.6	22.7	25.9	29.2	13.2	16.1
	[98.2,118.3]	[52.8,68.3]	[34.5,61.0]	[70.8,87.2]	[40.0,54.9]	[18.2,45.2]	[25.2,35.5]	[18.3,31.8]	[-2.7,14.2]	[42.6,55.5]	[18.4,28.1]	[16.4,35.5]	[24.4,35.1]	[10.4,16.7]	[9.3,22.9]
Burkina Faso	174.8	96.6	78.3	125.3	70.5	54.9	33.1	25.4	7.6	92.3	45.0	47.2	49.5	26.1	23.4
	[160.8,189.8]	[85.5,108.8]	[61.4,95.2]	[114.5,136.9]	[59.5,83.3]	[40.3,69.4]	[27.8,39.2]	[19.6,33.0]	[0.4,14.9]	[82.5,103.1]	[37.5,53.9]	[35.7,58.7]	[42.8,57.2]	[20.0,33.9]	[14.7,32.2]
Cameroon	110.7	49.0	61.7	74.7	43.1	31.6	29.9	24.5	5.4	44.9	18.6	26.2	36.0	5.9	30.1
	[96.7,126.4]	[41.2,58.2]	[42.8,80.6]	[63.9,87.2]	[36.0,51.6]	[15.0,48.2]	[22.9,38.9]	[18.5,32.4]	[-7.0,17.7]	[37.8,53.2]	[13.0,26.7]	[14.3,38.1]	[28.8,44.9]	[3.7,9.5]	[22.0,38.2]
Chad	160.6	138.2	22.4	120.5	98.5	22.0	35.5	36.8	-1.3	85.0	61.8	23.3	40.1	39.7	0.4
	[137.7,186.5]	[126.3,151.0]	[-3.2,48.0]	[98.5,146.7]	[89.7,108.1]	[-2.2,46.2]	[29.6,42.5]	[31.4,43.0]	[-10.3,7.8]	[66.5,108.2]	[54.7,69.7]	[3.5,43.1]	[34.8,46.0]	[33.6,46.7]	[-7.5,8.3]
Congo	89.3	54.4	34.9	63.5	41.6	21.9	22.1	19.2	2.9	41.3	22.4	18.9	25.9	12.8	13.0
	[82.4,96.8]	[42.4,69.6]	[17.1,52.7]	[57.0,70.6]	[30.9,55.8]	[6.9,36.8]	[18.5,26.5]	[11.6,31.6]	[-7.3,13.2]	[35.5,48.0]	[14.3,35.0]	[6.3,31.6]	[22.3,30.0]	[6.8,24.2]	[3.1,23.0]
Congo (DR)	117.1	76.4	40.8	84.8	63.3	21.5	26.6	22.1	4.5	58.2	41.3	17.0	32.3	13.0	19.3
	[103.5,132.3]	[67.0,86.9]	[23.0,58.6]	[75.0,95.8]	[54.3,73.7]	[4.2,38.9]	[22.0,32.2]	[16.6,29.3]	[-4.4,13.5]	[50.8,66.6]	[32.2,52.6]	[2.8,31.1]	[26.7,39.1]	[9.8,17.3]	[12.8,25.7]
Cote D'Ivoire	121.4	72.9	48.5	100.3	60.1	40.2	38.9	37.0	1.9	61.4	23.1	38.3	21.1	12.8	8.3
	[111.6,132.0]	[57.3,92.3]	[30.6,66.4]	[90.0,111.6]	[47.2,76.3]	[23.6,56.8]	[32.4,46.6]	[26.5,51.6]	[-13.1,16.8]	[54.0,69.9]	[16.0,33.2]	[26.2,50.5]	[16.6,26.8]	[6.9,23.4]	[-0.1,16.8]
Gabon	75.3	50.3	25.0	51.7	47.4	4.3	20.9	19.5	1.4	30.8	27.9	2.9	23.6	2.9	20.7
	[65.0,87.1]	[32.3,77.6]	[1.3,48.7]	[44.3,60.4]	[29.8,74.6]	[-17.2,25.8]	[16.4,26.6]	[9.1,41.4]	[-13.0,15.7]	[25.6,37.2]	[15.6,49.3]	[-11.7,17.6]	[17.9,31.0]	[1.1,7.6]	[13.2,28.2]
Gambia	76.6	39.1	37.6	58.0	34.4	23.6	35.0	21.9	13.1	23.0	12.5	10.5	18.6	4.7	13.9
	[65.6,89.4]	[29.9,50.8]	[24.3,50.9]	[50.1,67.1]	[25.4,46.4]	[12.7,34.6]	[29.3,41.9]	[14.6,32.9]	[2.4,23.8]	[18.6,28.3]	[7.1,21.7]	[1.6,19.5]	[13.4,25.7]	[2.0,10.6]	[7.6,20.2]
Ghana	92.0	63.6	28.4	67.6	58.0	9.7	32.4	40.5	-8.0	35.2	17.5	17.7	24.4	5.6	18.8
	[81.0,104.4]	[50.6,79.7]	[5.2,51.7]	[57.6,79.2]	[45.1,74.2]	[-11.9,31.2]	[25.3,41.4]	[29.3,55.5]	[-23.9,7.8]	[28.3,43.8]	[11.4,26.7]	[6.0,29.5]	[19.3,30.9]	[2.1,15.0]	[10.8,26.7]
Guinea	132.9	44.4	88.5	95.1	38.1	57.0	36.3	22.3	14.1	58.8	15.8	43.0	37.8	6.3	31.5
	[114.1,154.3]	[34.2,57.4]	[68.8,108.3]	[80.9,111.5]	[28.8,50.2]	[40.8,73.2]	[28.7,46.0]	[15.1,32.8]	[1.5,26.6]	[48.6,70.9]	[11.0,22.6]	[32.1,53.9]	[30.3,47.2]	[3.4,11.7]	[23.6,39.4]
Liberia	129.8	99.4	30.4	102.7	78.2	24.5	38.0	32.4	5.6	64.6	45.7	18.9	27.2	21.2	6.0
	[114.2,147.3]	[81.2,121.1]	[4.9,56.0]	[91.4,115.1]	[62.0,98.1]	[1.7,47.3]	[31.9,45.3]	[22.6,46.3]	[-6.1,17.2]	[56.3,74.1]	[34.4,60.6]	[2.7,35.2]	[19.8,37.2]	[13.6,33.1]	[-5.8,17.7]
Mali	142.6	57.3	85.3	98.3	42.8	55.6	41.8	23.9	17.9	56.6	18.9	37.7	44.3	14.5	29.8
	[128.6,157.9]	[47.7,68.6]	[69.2,101.5]	[86.8,111.2]	[36.0,50.7]	[42.6,68.5]	[33.7,51.7]	[18.3,31.1]	[8.2,27.6]	[48.4,66.0]	[14.2,25.1]	[29.7,45.6]	[37.4,52.4]	[9.9,21.2]	[22.1,37.5]
Mauritania	67.5	39.7	27.8	53.0	34.9	18.1	33.8	20.7	13.1	19.2	14.2	5.0	14.5	4.8	9.7
	[56.8,80.2]	[32.2,48.9]	[15.3,40.4]	[44.6,62.9]	[27.4,44.3]	[6.1,30.2]	[28.2,40.5]	[16.1,26.6]	[2.0,24.2]	[14.4,25.6]	[9.2,21.9]	[-2.2,12.3]	[10.4,20.2]	[2.3,10.0]	[3.8,15.6]
Niger	143.9	113.7	30.2	90.2	73.5	16.7	34.8	27.5	7.3	55.4	46.0	9.4	53.7	40.2	13.4
	[129.0,160.1]	[99.2,130.1]	[10.3,50.0]	[78.8,103.0]	[64.5,83.6]	[1.2,32.2]	[28.7,42.1]	[22.4,33.9]	[-0.5,15.1]	[46.0,66.5]	[39.1,54.0]	[-1.5,20.4]	[46.7,61.7]	[32.1,50.3]	[3.5,23.4]
Nigeria	172.8	53.4	119.4	108.7	45.6	63.0	38.9	27.7	11.2	69.8	17.9	51.9	64.1	7.8	56.3
	[161.3,184.9]	[48.2,59.1]	[105.8,133.0]	[101.0,116.9]	[40.7,51.1]	[54.6,71.5]	[34.8,43.3]	[23.5,32.7]	[5.8,16.5]	[63.8,76.3]	[15.1,21.2]	[45.1,58.7]	[57.4,71.5]	[6.0,10.1]	[48.6,64.1]

© 2022 Karlsson O et al. *JAMA Network Open*.

Sao Tome & Principe	62.5	18.3	44.2	42.8	17.8	25.0	16.1	11.0	5.1	26.6	6.8	19.8	19.7	0.5	19.2
	[45.3,85.6]	[8.5,39.2]	[20.9,67.4]	[31.2,58.3]	[8.0,39.0]	[6.3,43.6]	[10.1,25.7]	[4.2,29.0]	[-8.5,18.7]	[17.9,39.5]	[1.8,24.8]	[7.0,32.7]	[10.2,37.9]	[0.1,2.9]	[8.7,29.8]
Senegal	72.1	27.2	45.0	56.0	26.1	29.9	27.3	20.1	7.3	28.6	6.0	22.6	16.2	1.1	15.1
	[58.1,89.2]	[17.8,41.2]	[27.5,62.4]	[45.5,68.6]	[16.8,40.3]	[15.4,44.4]	[22.5,33.3]	[11.8,33.8]	[-4.2,18.8]	[20.9,39.1]	[2.7,13.2]	[12.8,32.5]	[11.1,23.6]	[0.3,4.7]	[9.0,21.1]
Sierra Leone	144.8	89.1	55.7	108.1	74.5	33.7	29.7	33.2	-3.4	78.4	41.3	37.1	36.6	14.6	22.0
	[130.3,160.5]	[74.4,106.4]	[35.9,75.4]	[95.5,122.2]	[61.7,89.6]	[16.1,51.2]	[24.2,36.5]	[24.7,44.4]	[-15.2,8.3]	[68.2,90.1]	[33.0,51.6]	[22.8,51.4]	[29.7,45.1]	[10.5,20.3]	[12.2,31.8]
Togo	97.4	43.8	53.6	64.0	33.9	30.1	24.2	26.7	-2.5	39.8	7.2	32.7	33.3	9.9	23.4
	[78.0,120.9]	[32.1,59.6]	[30.0,77.1]	[53.9,75.8]	[24.6,46.5]	[14.9,45.3]	[17.5,33.4]	[18.5,38.4]	[-13.9,8.8]	[31.4,50.4]	[3.6,14.1]	[23.0,42.3]	[21.2,52.0]	[4.7,20.6]	[5.4,41.5]
Latin America & Caribbean	41.7	18.8	22.9	36.3	17.3	19.0	17.5	11.4	6.2	18.8	5.9	12.9	5.4	1.5	3.9
	[39.3,44.4]	[15.9,22.2]	[18.1,27.8]	[34.1,38.7]	[14.4,20.8]	[14.4,23.6]	[15.8,19.5]	[9.6,13.5]	[3.0,9.3]	[17.2,20.5]	[4.1,8.6]	[9.7,16.0]	[4.6,6.3]	[1.0,2.2]	[2.5,5.3]
Colombia	27.2	6.8	20.3	24.9	6.0	18.8	12.1	4.7	7.4	12.8	1.3	11.5	2.3	0.8	1.5
	[22.4,32.9]	[3.4,13.6]	[11.6,29.1]	[20.2,30.6]	[2.8,12.9]	[10.6,27.1]	[8.4,17.4]	[2.0,10.9]	[1.3,13.5]	[9.9,16.5]	[0.4,4.3]	[7.7,15.2]	[1.4,3.9]	[0.2,3.2]	[-0.0,3.0]
Dominican Republic	34.3	26.4	7.9	30.7	26.1	4.7	20.6	23.2	-2.6	10.1	2.9	7.3	3.6	0.3	3.3
	[29.7,39.7]	[19.0,36.5]	[-2.8,18.7]	[26.6,35.5]	[18.7,36.3]	[-5.9,15.3]	[17.5,24.3]	[16.3,32.9]	[-12.6,7.5]	[7.6,13.6]	[1.4,5.9]	[4.0,10.5]	[2.1,6.0]	[0.1,1.5]	[1.5,5.1]
El Salvador	30.7	13.2	17.5	26.0	12.8	13.2	12.1	9.6	2.5	13.9	3.3	10.7	4.7	0.3	4.4
	[22.8,41.4]	[6.5,26.4]	[5.1,30.0]	[18.8,35.9]	[6.3,26.2]	[1.0,25.3]	[7.4,19.6]	[4.2,21.8]	[-7.3,12.3]	[9.5,20.3]	[1.0,11.0]	[5.2,16.1]	[2.3,9.4]	[0.1,1.7]	[0.4,8.4]
Guatemala	56.0	20.1	36.0	49.6	18.0	31.6	24.1	12.8	11.3	25.5	5.2	20.3	6.4	2.0	4.4
	[49.4,63.5]	[15.2,26.4]	[27.1,44.8]	[43.4,56.6]	[13.3,24.3]	[23.5,39.6]	[20.0,28.9]	[9.2,17.8]	[5.3,17.2]	[21.2,30.7]	[3.2,8.5]	[13.2,27.4]	[4.4,9.4]	[0.8,5.0]	[1.0,7.9]
Guyana	39.4	30.6	8.7	36.0	26.7	9.3	18.1	24.2	-6.1	17.9	2.5	15.4	3.4	3.9	-0.6
	[28.9,53.4]	[19.3,48.4]	[-10.4,27.9]	[26.4,48.9]	[17.0,41.6]	[-7.9,26.4]	[10.5,31.0]	[14.6,39.7]	[-23.1,10.9]	[12.1,26.3]	[0.8,8.3]	[9.6,21.1]	[1.4,8.1]	[0.7,21.1]	[-6.7,5.5]
Haiti	93.3	58.6	34.7	75.4	50.9	24.5	27.6	31.3	-3.7	47.8	19.6	28.2	17.8	7.7	10.2
	[77.0,112.5]	[45.4,75.4]	[17.9,51.4]	[60.4,93.8]	[37.4,69.1]	[7.5,41.5]	[20.2,37.6]	[21.7,44.9]	[-18.3,10.8]	[37.6,60.6]	[10.8,35.2]	[15.2,41.2]	[13.3,23.9]	[3.6,16.1]	[4.3,16.1]
Honduras	38.9	20.2	18.7	32.9	19.5	13.4	19.0	12.5	6.5	13.9	7.0	7.0	6.0	0.8	5.2
	[33.3,45.4]	[15.0,27.4]	[10.0,27.4]	[27.7,39.1]	[14.2,26.6]	[4.9,22.0]	[15.0,24.1]	[8.3,18.8]	[0.1,12.8]	[11.0,17.5]	[4.1,11.7]	[1.9,12.0]	[4.0,8.9]	[0.1,5.0]	[2.7,7.8]
Paraguay	29.5	4.5	25.0	28.3	4.5	23.8	12.2	3.7	8.5	16.1	0.8	15.3	1.2	NA	NA
	[20.7,42.0]	[1.7,11.6]	[15.2,34.8]	[19.6,40.6]	[1.7,11.6]	[14.1,33.4]	[7.0,21.2]	[1.4,10.3]	[1.6,15.4]	[10.1,25.6]	[0.1,4.8]	[6.6,24.0]	[0.4,4.0]		
Peru	35.8	14.2	21.6	31.1	13.9	17.2	16.2	5.6	10.5	14.9	8.3	6.7	4.7	0.3	4.4
	[31.8,40.3]	[7.2,28.0]	[11.2,32.0]	[27.4,35.2]	[6.9,27.9]	[7.3,27.0]	[13.1,20.0]	[3.0,10.8]	[5.1,16.0]	[12.2,18.3]	[2.7,25.4]	[-1.7,15.0]	[3.3,6.7]	[0.0,2.0]	[2.3,6.6]
Suriname	20.5	10.5	10.0	17.7	10.5	7.2	13.4	6.5	6.9	4.2	4.0	0.2	2.8	NA	NA
	[13.3,31.2]	[3.5,31.1]	[-3.1,23.0]	[11.6,26.9]	[3.5,31.1]	[-4.2,18.5]	[8.2,21.8]	[1.9,22.6]	[0.3,13.5]	[1.7,10.5]	[0.9,17.3]	[-8.2,8.6]	[1.2,6.7]		
Low-Income Countries	103.7	65.6	38.0	78.8	54.2	24.6	31.6	26.8	4.8	47.2	27.4	19.8	24.8	11.4	13.4
	[99.2,108.3]	[62.0,69.4]	[31.8,44.3]	[75.1,82.7]	[50.7,58.0]	[19.1,30.0]	[29.4,34.0]	[24.3,29.6]	[1.2,8.4]	[44.5,50.1]	[24.9,30.2]	[16.2,23.5]	[23.3,26.4]	[10.1,12.9]	[11.5,15.3]
Lower-Middle-Income Countries	85.9	35.5	50.4	68.6	31.9	36.7	36.8	21.2	15.6	31.8	10.8	21.0	17.3	3.5	13.8
	[83.0,88.8]	[33.8,37.2]	[47.7,53.2]	[66.7,70.5]	[30.3,33.6]	[34.1,39.2]	[35.8,37.9]	[20.0,22.5]	[13.7,17.5]	[30.2,33.4]	[9.9,11.6]	[19.4,22.7]	[15.9,18.8]	[3.1,4.0]	[12.6,14.9]
Upper-Middle-Income Countries	44.4	21.1	23.3	38.8	19.4	19.5	19.1	11.7	7.4	19.7	7.6	12.1	5.6	1.8	3.8
	[41.5,47.5]	[17.4,25.7]	[18.1,28.4]	[36.2,41.6]	[15.9,23.6]	[14.5,24.4]	[17.2,21.3]	[8.9,15.4]	[3.4,11.3]	[17.6,22.1]	[6.1,9.5]	[8.9,15.3]	[4.3,7.2]	[1.1,2.9]	[2.3,5.3]

Notes: 95% confidence intervals are shown in brackets. 'Worst' refers to children in the 20% of households with the worst living standards and 'Best' refers to children in the 20% of households with the best living standards, within each country.

© 2022 Karlsson O et al. *JAMA Network Open*.

eTable 8. Shares of Deaths Among Children Younger Than 5 Years Occurring at Different Ages by Living Standards

	0–23 months			Neonates			1–23 months			24–59 months		
	Worst	Best	Difference	Worst	Best	Difference	Worst	Best	Difference	Worst	Best	Difference
Pooled	79.5	87.7	-8.2	39.9	52.8	-13.0	39.6	34.8	4.8	20.5	12.3	8.2
	[78.6,80.3]	[86.7,88.6]	[-9.2,-7.3]	[38.7,41.0]	[51.2,54.5]	[-15.4,-10.5]	[38.5,40.7]	[33.2,36.5]	[2.3,7.2]	[19.7,21.4]	[11.4,13.3]	[7.3,9.2]
Least Developed Countries	77.5	84.3	-6.7	32.7	44.1	-11.4	44.8	40.1	4.7	22.5	15.7	6.7
	[76.6,78.5]	[82.7,85.7]	[-8.8,-4.6]	[31.4,34.0]	[41.2,47.1]	[-14.3,-8.5]	[43.6,46.1]	[37.3,43.0]	[1.8,7.6]	[21.5,23.4]	[14.3,17.3]	[4.6,8.8]
East Asia & Pacific	85.4	90.5	-5.0	40.5	56.5	-16.0	45.0	34.0	11.0	14.6	9.5	5.0
	[83.0,87.6]	[84.8,94.2]	[-8.9,-1.2]	[36.7,44.4]	[48.5,64.2]	[-23.3,-8.8]	[41.1,48.8]	[27.3,41.3]	[3.3,18.7]	[12.4,17.0]	[5.8,15.2]	[1.2,8.9]
Cambodia	88.1	90.0	-1.9	35.1	62.8	-27.7	53.0	27.2	25.8	11.9	10.0	1.9
	[81.0,92.8]	[75.0,96.4]	[-11.5,7.7]	[27.3,43.7]	[46.1,76.9]	[-45.6,-9.8]	[43.6,62.2]	[15.1,43.9]	[10.4,41.3]	[7.2,19.0]	[3.6,25.0]	[-7.7,11.5]
Indonesia	86.4	89.8	-3.5	43.0	52.8	-9.8	43.3	37.0	6.3	13.6	10.2	3.5
	[80.8,90.5]	[82.7,94.2]	[-11.0,4.0]	[36.9,49.4]	[43.3,62.2]	[-20.5,1.0]	[37.7,49.1]	[28.2,46.8]	[-5.8,18.4]	[9.5,19.2]	[5.8,17.3]	[-4.0,11.0]
Lao	91.1	87.0	4.0	35.3	68.3	-33.1	55.8	18.7	37.1	8.9	13.0	-4.0
	[88.1,93.4]	[72.4,94.5]	[-10.2,18.3]	[31.0,39.8]	[52.3,80.9]	[-49.4,-16.7]	[50.5,61.0]	[9.2,34.2]	[24.3,49.9]	[6.6,11.9]	[5.5,27.6]	[-18.3,10.2]
Mongolia	85.2	76.4	8.9	45.5	45.4	0.2	39.7	31.0	8.7	14.8	23.6	-8.9
	[71.6,92.9]	[40.2,93.9]	[-22.8,40.5]	[33.8,57.8]	[23.1,69.7]	[-23.6,23.9]	[28.6,52.0]	[13.7,55.9]	[-17.0,34.4]	[7.1,28.4]	[6.1,59.8]	[-40.5,22.8]
Myanmar	85.7	92.4	-6.7	35.5	66.0	-30.5	50.2	26.4	23.8	14.3	7.6	6.7
	[79.1,90.4]	[70.5,98.4]	[-18.9,5.5]	[29.5,41.9]	[42.5,83.5]	[-52.9,-8.1]	[43.5,56.9]	[12.0,48.6]	[0.1,47.4]	[9.6,20.9]	[1.6,29.5]	[-5.5,18.9]
Papua New Guinea	79.0	86.2	-7.2	41.5	54.8	-13.4	37.6	31.3	6.2	21.0	13.8	7.2
	[70.7,85.5]	[76.8,92.2]	[-17.2,2.9]	[33.6,49.7]	[46.4,63.0]	[-28.0,1.2]	[29.6,46.2]	[24.8,38.7]	[-6.6,19.1]	[14.5,29.3]	[7.8,23.2]	[-2.9,17.2]
Philippines	83.1	95.7	-12.6	42.7	68.0	-25.3	40.5	27.7	12.7	16.9	4.3	12.6
	[76.6,88.1]	[73.4,99.5]	[-21.2,-4.0]	[34.7,51.0]	[41.8,86.2]	[-47.0,-3.7]	[35.1,46.1]	[11.3,53.6]	[-8.4,33.8]	[11.9,23.4]	[0.5,26.6]	[4.0,21.2]
Timor-Leste	73.6	81.1	-7.5	30.7	66.0	-35.3	42.9	15.1	27.8	26.4	18.9	7.5
	[64.3,81.2]	[66.7,90.2]	[-19.9,4.9]	[23.2,39.5]	[50.2,79.0]	[-50.6,-20.0]	[36.6,49.5]	[7.6,27.7]	[14.0,41.7]	[18.8,35.7]	[9.8,33.3]	[-4.9,19.9]
Tonga	NA	NA	NA	37.3	NA	NA	62.7	NA	NA	NA	NA	NA
				[9.6,77.0]			[23.0,90.4]					
South Asia	86.9	92.0	-5.1	54.3	65.8	-11.5	32.6	26.2	6.4	13.1	8.0	5.1
	[85.8,87.9]	[89.2,94.1]	[-7.5,-2.7]	[53.0,55.6]	[62.4,69.1]	[-15.5,-7.6]	[31.1,34.1]	[23.5,29.0]	[3.1,9.8]	[12.1,14.2]	[5.9,10.8]	[2.7,7.5]
Afghanistan	88.0	91.2	-3.2	39.9	43.2	-3.3	48.1	48.0	0.1	12.0	8.8	3.2
	[84.4,90.9]	[86.1,94.6]	[-7.6,1.2]	[34.3,45.7]	[32.8,54.3]	[-19.4,12.7]	[42.5,53.8]	[35.8,60.5]	[-15.7,15.9]	[9.1,15.6]	[5.4,13.9]	[-1.2,7.6]
Bangladesh	88.0	91.1	-3.1	59.7	66.7	-7.0	28.2	24.4	3.9	12.0	8.9	3.1
	[85.0,90.5]	[86.4,94.3]	[-8.1,1.9]	[55.2,64.2]	[57.9,74.5]	[-14.8,0.9]	[23.9,33.0]	[17.4,33.0]	[-4.2,11.9]	[9.5,15.0]	[5.7,13.6]	[-1.9,8.1]
India	86.8	91.3	-4.5	55.3	62.9	-7.6	31.5	28.4	3.1	13.2	8.7	4.5
	[86.1,87.5]	[88.9,93.3]	[-7.4,-1.7]	[53.8,56.8]	[59.5,66.3]	[-10.7,-4.6]	[30.3,32.8]	[25.0,32.0]	[0.2,6.0]	[12.5,13.9]	[6.7,11.1]	[1.7,7.4]
Maldives	77.7	91.1	-13.4	53.0	91.1	-38.1	24.7	NA	NA	22.3	8.9	13.4
	[60.5,88.8]	[49.7,99.1]	[-45.0,18.1]	[35.9,69.5]	[49.7,99.1]	[-68.7,-7.6]	[13.8,40.3]			[11.2,39.5]	[0.9,50.3]	[-18.1,45.0]
Nepal	89.1	76.9	12.2	55.3	65.0	-9.7	33.9	11.9	21.9	10.9	23.1	-12.2
	[80.7,94.2]	[35.2,95.3]	[-16.0,40.4]	[43.3,66.7]	[31.0,88.5]	[-39.0,19.5]	[24.3,44.9]	[3.4,34.4]	[6.4,37.5]	[5.8,19.3]	[4.7,64.8]	[-40.4,16.0]
Pakistan	86.5	94.1	-7.6	51.2	75.0	-23.8	35.2	19.1	16.2	13.5	5.9	7.6
	[82.9,89.3]	[85.9,97.6]	[-13.7,-1.6]	[44.5,57.9]	[63.0,84.1]	[-33.6,-14.0]	[28.2,42.9]	[12.3,28.5]	[7.1,25.2]	[10.7,17.1]	[2.4,14.1]	[1.6,13.7]
Europe & Central Asia	91.7	95.5	-3.8	43.5	45.9	-2.4	48.2	49.7	-1.5	8.3	4.5	3.8
	[83.9,95.9]	[87.5,98.5]	[-11.2,3.5]	[34.8,52.5]	[24.6,68.7]	[-22.8,18.0]	[39.9,56.6]	[26.7,72.7]	[-24.6,21.7]	[4.1,16.1]	[1.5,12.5]	[-3.5,11.2]
Albania	96.2	NA	NA	36.4	NA	NA	59.8	NA	NA	3.8	NA	NA
	[76.4,99.5]			[13.9,67.0]			[29.3,84.3]			[0.5,23.6]		
Armenia	NA	NA	NA	27.4	47.8	-20.4	72.6	52.2	20.4	NA	NA	NA
				[8.7,60.0]	[6.6,92.2]	[-84.8,44.0]	[40.0,91.3]	[7.8,93.4]	[-44.0,84.8]			
Kyrgyz Republic	99.2	95.4	3.8	59.9	72.5	-12.6	39.3	22.9	16.4	0.8	4.6	-3.8
	[95.0,99.9]	[74.2,99.3]	[-7.0,14.6]	[35.7,80.0]	[48.5,88.1]	[-50.0,24.8]	[19.4,63.5]	[9.2,46.4]	[-22.6,55.5]	[0.1,5.0]	[0.7,25.8]	[-14.6,7.0]
Moldova	NA	NA	NA	75.3	81.7	-6.4	24.7	18.3	6.4	NA	NA	NA

© 2022 Karlsson O et al. *JAMA Network Open*.

				[41.2,93.0]	[41.5,96.6]	[-35.3,22.6]	[7.0,58.8]	[3.4,58.5]	[-22.6,35.3]			
Tajikistan	96.7	95.8	0.9	42.1	58.8	-16.7	54.5	36.9	17.6	3.3	4.2	-0.9
	[90.6,98.9]	[87.0,98.7]	[-3.7,5.5]	[32.1,52.8]	[44.0,72.2]	[-35.5,2.1]	[44.3,64.4]	[23.9,52.2]	[-0.9,36.2]	[1.1,9.4]	[1.3,13.0]	[-5.5,3.7]
Turkey	89.8	95.8	-6.1	40.1	22.5	17.5	49.7	73.3	-23.6	10.2	4.2	6.1
	[78.4,95.5]	[9.6,100.0]	[-20.8,8.7]	[27.5,54.1]	[3.3,71.0]	[-22.0,57.1]	[36.0,63.5]	[28.8,94.9]	[-63.9,16.7]	[4.5,21.6]	[0.0,90.4]	[-8.7,20.8]
Turkmenistan	86.3	92.6	-6.3	53.5	71.0	-17.6	32.8	21.6	11.3	13.7	7.4	6.3
	[75.0,92.9]	[75.6,98.0]	[-20.3,7.7]	[41.2,65.4]	[54.5,83.3]	[-39.9,4.8]	[21.2,47.0]	[10.6,39.0]	[-11.1,33.6]	[7.1,25.0]	[2.0,24.4]	[-7.7,20.3]
Middle East & North Africa	90.1	93.5	-3.4	50.0	56.3	-6.3	40.1	37.2	2.9	9.9	6.5	3.4
	[87.7,92.1]	[89.0,96.3]	[-7.1,0.2]	[45.9,54.2]	[49.8,62.6]	[-13.7,1.1]	[36.4,43.9]	[31.4,43.5]	[-4.9,10.7]	[7.9,12.3]	[3.7,11.0]	[-0.2,7.1]
Egypt	91.4	96.9	-5.5	54.7	57.9	-3.2	36.8	39.1	-2.3	8.6	3.1	5.5
	[85.6,95.1]	[87.0,99.3]	[-12.4,1.4]	[47.4,61.7]	[47.5,67.5]	[-16.6,10.2]	[30.4,43.7]	[29.7,49.3]	[-15.8,11.3]	[4.9,14.4]	[0.7,13.0]	[-1.4,12.4]
Iraq	87.1	87.5	-0.4	52.4	48.9	3.6	34.7	38.6	-4.0	12.9	12.5	0.4
	[83.4,90.0]	[75.6,94.0]	[-11.9,11.1]	[46.4,58.4]	[36.4,61.5]	[-11.3,18.4]	[29.2,40.5]	[27.3,51.3]	[-16.6,8.7]	[10.0,16.6]	[6.0,24.4]	[-11.1,11.9]
Jordan	92.9	99.4	-6.5	51.8	66.9	-15.2	41.2	32.5	8.7	7.1	0.6	6.5
	[86.7,96.4]	[92.2,100.0]	[-11.8,-1.2]	[36.4,66.8]	[37.3,87.3]	[-41.3,11.0]	[26.2,57.9]	[12.4,62.0]	[-17.6,35.0]	[3.6,13.3]	[0.0,7.8]	[1.2,11.8]
State of Palestine	95.6	87.1	8.5	43.8	39.4	4.4	51.8	47.7	4.1	4.4	12.9	-8.5
	[88.2,98.4]	[72.8,94.5]	[-2.9,19.9]	[32.6,55.7]	[28.3,51.8]	[-14.5,23.3]	[39.2,64.1]	[33.4,62.4]	[-15.5,23.7]	[1.6,11.8]	[5.5,27.2]	[-19.9,2.9]
Tunisia	88.1	87.6	0.5	58.8	57.1	1.7	29.3	30.5	-1.1	11.9	12.4	-0.5
	[68.7,96.2]	[48.4,98.2]	[-28.6,29.6]	[38.9,76.1]	[24.2,84.7]	[-42.2,45.5]	[16.5,46.7]	[8.5,67.4]	[-42.7,40.4]	[3.8,31.3]	[1.8,51.6]	[-29.6,28.6]
Yemen	89.5	92.7	-3.2	41.3	59.1	-17.8	48.3	33.6	14.6	10.5	7.3	3.2
	[86.6,91.9]	[87.1,96.0]	[-7.2,0.8]	[35.3,47.5]	[47.1,70.1]	[-30.0,-5.7]	[42.0,54.6]	[23.8,45.1]	[2.6,26.7]	[8.1,13.4]	[4.0,12.9]	[-0.8,7.2]
Eastern & Southern Africa	78.8	88.1	-9.3	34.3	50.3	-16.0	44.4	37.8	6.7	21.2	11.9	9.3
	[77.1,80.4]	[86.0,89.9]	[-12.3,-6.3]	[31.9,36.9]	[46.0,54.6]	[-20.3,-11.7]	[42.1,46.8]	[33.7,42.0]	[2.5,10.9]	[19.6,22.9]	[10.1,14.0]	[6.3,12.3]
Angola	72.6	74.2	-1.6	30.4	29.7	0.7	42.2	44.5	-2.3	27.4	25.8	1.6
	[68.4,76.5]	[63.7,82.6]	[-12.6,9.4]	[24.2,37.5]	[19.4,42.7]	[-12.5,13.9]	[36.2,48.4]	[33.6,56.0]	[-15.6,11.0]	[23.5,31.6]	[17.4,36.3]	[-9.4,12.6]
Burundi	75.2	82.8	-7.6	28.4	40.6	-12.2	46.8	42.2	4.7	24.8	17.2	7.6
	[71.3,78.7]	[76.3,87.8]	[-14.8,-0.4]	[24.0,33.3]	[34.2,47.4]	[-20.8,-3.7]	[41.3,52.4]	[36.4,48.1]	[-2.9,12.2]	[21.3,28.7]	[12.2,23.7]	[0.4,14.8]
Comoros	75.8	95.5	-19.7	36.4	56.7	-20.3	39.4	38.8	0.6	24.2	4.5	19.7
	[57.1,88.1]	[75.1,99.3]	[-41.1,1.6]	[22.8,52.6]	[31.8,78.6]	[-49.7,9.0]	[22.7,59.1]	[17.9,64.9]	[-27.8,29.0]	[11.9,42.9]	[0.7,24.9]	[-1.6,41.1]
Ethiopia	79.6	91.1	-11.5	40.4	60.0	-19.6	39.2	31.1	8.1	20.4	8.9	11.5
	[74.4,84.0]	[77.7,96.8]	[-20.9,-2.0]	[31.4,50.2]	[44.6,73.7]	[-34.7,-4.5]	[31.2,47.8]	[20.7,43.8]	[-4.3,20.6]	[16.0,25.6]	[3.2,22.3]	[2.0,20.9]
Kenya	80.8	90.0	-9.1	35.4	55.0	-19.5	45.4	35.0	10.4	19.2	10.0	9.1
	[77.0,84.2]	[81.5,94.8]	[-16.6,-1.6]	[31.0,40.2]	[45.6,64.0]	[-28.9,-10.2]	[40.9,50.0]	[26.1,45.1]	[1.3,19.5]	[15.8,23.0]	[5.2,18.5]	[1.6,16.6]
Lesotho	91.5	81.1	10.4	43.1	47.8	-4.7	48.3	33.2	15.1	8.5	18.9	-10.4
	[85.3,95.2]	[61.9,91.9]	[-5.3,26.1]	[34.7,52.0]	[30.8,65.4]	[-25.8,16.4]	[39.4,57.4]	[21.4,47.7]	[-4.7,34.9]	[4.8,14.7]	[8.1,38.1]	[-26.1,5.3]
Madagascar	68.8	84.6	-15.8	30.3	48.8	-18.5	38.5	35.8	2.7	31.2	15.4	15.8
	[62.0,74.9]	[73.9,91.4]	[-25.8,-5.8]	[24.6,36.7]	[38.6,59.1]	[-30.4,-6.6]	[33.8,43.4]	[24.3,49.2]	[-9.1,14.5]	[25.1,38.0]	[8.6,26.1]	[5.8,25.8]
Malawi	73.4	81.8	-8.4	33.1	42.1	-9.1	40.3	39.7	0.7	26.6	18.2	8.4
	[68.6,77.7]	[74.1,87.6]	[-15.1,-1.8]	[28.7,37.7]	[34.2,50.5]	[-16.6,-1.6]	[35.7,45.1]	[32.9,46.9]	[-6.3,7.6]	[22.3,31.4]	[12.4,25.9]	[1.8,15.1]
Mozambique	75.9	85.3	-9.4	25.6	36.0	-10.4	50.3	49.3	0.9	24.1	14.7	9.4
	[70.2,80.8]	[80.6,89.0]	[-14.8,-4.1]	[20.9,30.9]	[30.4,42.0]	[-17.6,-3.2]	[44.0,56.5]	[43.2,55.5]	[-6.5,8.4]	[19.2,29.8]	[11.0,19.4]	[4.1,14.8]
Namibia	86.9	85.4	1.5	34.5	37.3	-2.8	52.4	48.1	4.3	13.1	14.6	-1.5
	[80.1,91.7]	[65.1,94.8]	[-11.7,14.7]	[26.0,44.2]	[23.2,54.0]	[-20.7,15.2]	[43.6,61.1]	[32.2,64.5]	[-17.6,26.1]	[8.3,19.9]	[5.2,34.9]	[-14.7,11.7]
Rwanda	75.3	79.5	-4.1	27.8	33.0	-5.2	47.5	46.4	1.1	24.7	20.5	4.1
	[69.0,80.7]	[72.0,85.4]	[-13.4,5.1]	[22.4,34.0]	[23.6,44.1]	[-16.7,6.3]	[41.3,53.8]	[35.7,57.5]	[-10.1,12.3]	[19.3,31.0]	[14.6,28.0]	[-5.1,13.4]
South Africa	87.6	96.3	-8.7	37.7	71.1	-33.5	50.0	25.2	24.8	12.4	3.7	8.7
	[78.4,93.3]	[81.3,99.4]	[-20.3,3.0]	[29.6,46.5]	[34.1,92.2]	[-65.6,-1.4]	[40.6,59.3]	[5.6,65.8]	[-9.2,58.8]	[6.7,21.6]	[0.6,18.7]	[-3.0,20.3]
South Sudan	81.5	83.6	-2.1	36.8	36.7	0.1	44.7	46.9	-2.2	18.5	16.4	2.1
	[76.5,85.7]	[77.3,88.4]	[-11.6,7.5]	[30.2,44.0]	[30.3,43.6]	[-9.6,9.8]	[37.4,52.3]	[40.8,53.0]	[-12.7,8.3]	[14.3,23.5]	[11.6,22.7]	[-7.5,11.6]
Sudan	87.2	89.0	-1.9	45.4	60.3	-14.8	41.7	28.7	13.0	12.8	11.0	1.9
	[83.7,90.0]	[82.4,93.3]	[-8.2,4.5]	[38.7,52.4]	[48.9,70.7]	[-25.3,-4.4]	[35.6,48.1]	[19.7,39.9]	[2.8,23.2]	[10.0,16.3]	[6.7,17.6]	[-4.5,8.2]
Swaziland	92.2	69.2	23.1	26.4	18.4	8.1	65.8	50.8	15.0	7.8	30.8	-23.1

© 2022 Karlsson O et al. *JAMA Network Open*.

	[85.6,96.0]	[39.3,88.6]	[-1.7,47.9]	[19.0,35.5]	[7.2,39.5]	[-6.4,22.6]	[56.5,74.0]	[29.3,72.1]	[-11.0,41.0]	[4.0,14.4]	[11.4,60.7]	[-47.9,1.7]
Tanzania	77.4	93.3	-15.8	26.1	51.3	-25.2	51.4	42.0	9.4	22.6	6.7	15.8
	[70.0,83.5]	[87.5,96.5]	[-25.0,-6.7]	[20.5,32.5]	[42.3,60.2]	[-37.5,-13.0]	[42.9,59.8]	[34.3,50.0]	[-1.2,20.0]	[16.5,30.0]	[3.5,12.5]	[6.7,25.0]
Uganda	78.4	84.5	-6.1	31.5	48.9	-17.4	47.0	35.6	11.4	21.6	15.5	6.1
	[74.7,81.7]	[77.1,89.8]	[-13.1,1.0]	[27.9,35.2]	[39.7,58.2]	[-27.4,-7.5]	[42.7,51.3]	[29.5,42.2]	[3.3,19.5]	[18.3,25.3]	[10.2,22.9]	[-1.0,13.1]
Zambia	77.0	92.4	-15.4	38.8	44.6	-5.8	38.2	47.8	-9.6	23.0	7.6	15.4
	[71.5,81.8]	[86.1,96.0]	[-21.4,-9.4]	[32.1,46.0]	[34.6,55.0]	[-18.5,7.0]	[30.9,46.1]	[38.5,57.3]	[-23.1,3.8]	[18.2,28.5]	[4.0,13.9]	[9.4,21.4]
Zimbabwe	82.6	85.2	-2.6	38.7	51.3	-12.6	43.9	33.8	10.1	17.4	14.8	2.6
	[75.6,87.9]	[77.9,90.3]	[-11.4,6.3]	[29.2,49.2]	[43.4,59.2]	[-27.8,2.5]	[36.7,51.3]	[25.7,43.0]	[-5.6,25.8]	[12.1,24.4]	[9.7,22.1]	[-6.3,11.4]
West & Central Africa	67.3	81.2	-13.8	24.2	41.5	-17.2	43.1	39.7	3.4	32.7	18.8	13.8
	[65.7,68.9]	[79.5,82.7]	[-16.1,-11.5]	[22.9,25.6]	[38.4,44.6]	[-20.5,-14.0]	[41.5,44.8]	[36.5,42.9]	[-0.4,7.2]	[31.1,34.3]	[17.3,20.5]	[11.5,16.1]
Benin	72.9	78.1	-5.2	27.8	40.2	-12.5	45.1	37.8	7.3	27.1	21.9	5.2
	[68.8,76.6]	[72.5,82.7]	[-13.5,3.2]	[23.9,32.0]	[32.4,48.6]	[-20.5,-4.4]	[40.8,49.5]	[30.5,45.8]	[-2.1,16.7]	[23.4,31.2]	[17.3,27.5]	[-3.2,13.5]
Burkina Faso	71.7	73.0	-1.3	18.9	26.3	-7.4	52.8	46.6	6.1	28.3	27.0	1.3
	[68.4,74.7]	[65.4,79.4]	[-8.0,5.4]	[15.8,22.5]	[20.9,32.6]	[-13.1,-1.7]	[49.6,55.9]	[41.3,52.1]	[0.7,11.5]	[25.3,31.6]	[20.6,34.6]	[-5.4,8.0]
Cameroon	67.5	88.0	-20.5	27.0	50.0	-23.0	40.5	38.0	2.5	32.5	12.0	20.5
	[61.5,73.0]	[81.7,92.3]	[-28.7,-12.3]	[22.1,32.5]	[37.5,62.4]	[-36.3,-9.7]	[34.7,46.6]	[27.5,49.8]	[-12.9,17.9]	[27.0,38.5]	[7.7,18.3]	[12.3,28.7]
Chad	75.1	71.3	3.8	22.1	26.6	-4.5	53.0	44.7	8.3	24.9	28.7	-3.8
	[70.3,79.3]	[67.7,74.7]	[-1.4,8.9]	[18.7,25.9]	[22.8,30.8]	[-9.9,0.9]	[47.7,58.2]	[41.2,48.2]	[2.7,13.9]	[20.7,29.7]	[25.3,32.3]	[-8.9,1.4]
Congo	71.0	76.4	-5.4	24.8	35.3	-10.5	46.3	41.1	5.1	29.0	23.6	5.4
	[66.8,74.9]	[60.2,87.4]	[-19.7,8.9]	[20.8,29.2]	[22.5,50.7]	[-26.1,5.1]	[41.2,51.4]	[26.8,57.2]	[-13.0,23.2]	[25.1,33.2]	[12.6,39.8]	[-8.9,19.7]
Congo (DR)	72.4	82.9	-10.5	22.7	28.9	-6.2	49.7	54.0	-4.3	27.6	17.1	10.5
	[68.8,75.8]	[77.8,87.1]	[-17.6,-3.4]	[19.4,26.4]	[21.4,37.8]	[-14.5,2.1]	[45.9,53.5]	[44.8,63.0]	[-14.4,5.8]	[24.2,31.2]	[12.9,22.2]	[3.4,17.6]
Cote D'Ivoire	82.6	82.5	0.1	32.0	50.8	-18.8	50.6	31.7	18.9	17.4	17.5	-0.1
	[78.1,86.4]	[71.7,89.8]	[-8.7,8.9]	[27.7,36.7]	[38.3,63.2]	[-31.5,-6.0]	[45.4,55.8]	[23.0,41.9]	[6.6,31.2]	[13.6,21.9]	[10.2,28.3]	[-8.9,8.7]
Gabon	68.7	94.3	-25.6	27.7	38.8	-11.1	41.0	55.5	-14.5	31.3	5.7	25.6
	[62.1,74.6]	[84.5,98.0]	[-35.8,-15.4]	[22.2,34.1]	[19.2,62.8]	[-32.8,10.6]	[35.6,46.6]	[33.4,75.6]	[-34.9,5.8]	[25.4,37.9]	[2.0,15.5]	[15.4,35.8]
Gambia	75.7	88.1	-12.3	45.7	56.2	-10.4	30.0	31.9	-1.9	24.3	11.9	12.3
	[69.4,81.1]	[74.0,95.0]	[-22.2,-2.5]	[40.3,51.3]	[39.5,71.6]	[-27.0,6.2]	[25.0,35.6]	[18.7,48.8]	[-18.9,15.1]	[18.9,30.6]	[5.0,26.0]	[2.5,22.2]
Ghana	73.5	91.1	-17.6	35.2	63.6	-28.4	38.3	27.5	10.8	26.5	8.9	17.6
	[67.3,78.8]	[77.7,96.8]	[-27.2,-8.0]	[28.9,42.1]	[50.7,74.8]	[-42.1,-14.7]	[31.6,45.4]	[17.8,40.0]	[-0.9,22.5]	[21.2,32.7]	[3.2,22.3]	[8.0,27.2]
Guinea	71.5	85.7	-14.2	27.3	50.1	-22.8	44.2	35.6	8.6	28.5	14.3	14.2
	[66.9,75.8]	[75.6,92.1]	[-22.0,-6.4]	[22.5,32.8]	[37.2,63.1]	[-36.9,-8.8]	[39.4,49.2]	[26.8,45.5]	[-3.9,21.1]	[24.2,33.1]	[7.9,24.4]	[6.4,22.0]
Liberia	79.1	78.6	0.4	29.3	32.6	-3.4	49.8	46.0	3.8	20.9	21.4	-0.4
	[73.7,83.6]	[68.6,86.1]	[-9.0,9.9]	[25.2,33.7]	[24.8,41.6]	[-11.8,5.0]	[44.9,54.7]	[35.3,57.1]	[-5.5,13.1]	[16.4,26.3]	[13.9,31.4]	[-9.9,9.0]
Mali	68.9	74.7	-5.7	29.3	41.7	-12.4	39.7	33.0	6.7	31.1	25.3	5.7
	[64.4,73.1]	[67.2,80.9]	[-13.2,1.8]	[23.9,35.3]	[33.1,50.8]	[-20.2,-4.5]	[35.6,43.9]	[25.1,42.0]	[-2.2,15.6]	[26.9,35.6]	[19.1,32.8]	[-1.8,13.2]
Mauritania	78.5	87.8	-9.3	50.0	52.1	-2.1	28.5	35.7	-7.3	21.5	12.2	9.3
	[72.5,83.5]	[76.1,94.2]	[-20.0,1.4]	[44.0,56.1]	[42.6,61.5]	[-16.9,12.7]	[22.9,34.9]	[25.2,47.9]	[-22.0,7.5]	[16.5,27.5]	[5.8,23.9]	[-1.4,20.0]
Niger	62.7	64.6	-1.9	24.2	24.2	-0.0	38.5	40.4	-1.9	37.3	35.4	1.9
	[58.6,66.6]	[59.7,69.3]	[-8.0,4.1]	[20.6,28.3]	[19.8,29.3]	[-4.5,4.5]	[33.5,43.7]	[36.1,44.9]	[-7.9,4.0]	[33.4,41.4]	[30.7,40.3]	[-4.1,8.0]
Nigeria	62.9	85.5	-22.6	22.5	51.9	-29.4	40.4	33.6	6.8	37.1	14.5	22.6
	[60.2,65.6]	[81.6,88.7]	[-27.2,-17.9]	[20.5,24.6]	[46.4,57.4]	[-35.3,-23.5]	[37.7,43.2]	[28.7,38.9]	[1.2,12.5]	[34.4,39.8]	[11.3,18.4]	[17.9,27.2]
Sao Tome & Principe	68.4	97.3	-28.8	25.8	60.3	-34.5	42.6	37.0	5.6	31.6	2.7	28.8
	[52.2,81.2]	[75.0,99.8]	[-43.6,-14.1]	[15.3,40.2]	[21.2,89.6]	[-78.1,9.2]	[32.1,53.8]	[9.3,77.1]	[-40.4,51.6]	[18.8,47.8]	[0.2,25.0]	[14.1,43.6]
Senegal	77.6	96.0	-18.4	37.9	73.9	-36.0	39.7	22.1	17.6	22.4	4.0	18.4
	[71.2,82.9]	[82.8,99.2]	[-26.3,-10.5]	[31.2,45.1]	[54.3,87.1]	[-57.5,-14.5]	[32.9,46.9]	[9.7,42.8]	[-2.1,37.3]	[17.1,28.8]	[0.8,17.2]	[10.5,26.3]
Sierra Leone	74.7	83.6	-8.9	20.5	37.2	-16.7	54.2	46.4	7.8	25.3	16.4	8.9
	[69.8,79.0]	[78.6,87.6]	[-16.4,-1.4]	[17.0,24.5]	[30.6,44.5]	[-26.1,-7.3]	[49.5,58.8]	[38.5,54.4]	[-3.9,19.5]	[21.0,30.2]	[12.4,21.4]	[1.4,16.4]
Togo	65.8	77.4	-11.6	24.9	61.0	-36.2	40.9	16.4	24.5	34.2	22.6	11.6
	[55.8,74.5]	[62.5,87.5]	[-30.2,6.9]	[17.5,34.0]	[46.2,74.1]	[-52.4,-20.0]	[32.4,49.9]	[8.5,29.1]	[12.9,36.2]	[25.5,44.2]	[12.5,37.5]	[-6.9,30.2]
Latin America & Caribbean	87.1	92.2	-5.1	42.0	60.6	-18.6	45.1	31.6	13.5	12.9	7.8	5.1

© 2022 Karlsson O et al. *JAMA Network Open*.

	[85.2,88.7]	[87.8,95.1]	[-10.0,-0.2]	[38.7,45.5]	[52.3,68.3]	[-29.0,-8.1]	[42.1,48.1]	[23.8,40.6]	[2.9,24.0]	[11.3,14.8]	[4.9,12.2]	[0.2,10.0]
Colombia	91.6	88.4	3.2	44.5	69.1	-24.6	47.0	19.3	27.8	8.4	11.6	-3.2
	[86.0,95.0]	[51.3,98.2]	[-11.9,18.2]	[33.4,56.3]	[40.9,87.9]	[-52.2,3.0]	[37.4,56.9]	[5.8,48.0]	[1.0,54.5]	[5.0,14.0]	[1.8,48.7]	[-18.2,11.9]
Dominican Republic	89.6	98.9	-9.3	60.1	88.0	-27.9	29.5	10.9	18.6	10.4	1.1	9.3
	[83.8,93.5]	[92.6,99.8]	[-14.2,-4.4]	[51.9,67.8]	[77.8,93.9]	[-39.5,-16.3]	[23.3,36.6]	[5.4,20.8]	[8.6,28.7]	[6.5,16.2]	[0.2,7.4]	[4.4,14.2]
El Salvador	84.7	97.4	-12.7	39.3	72.7	-33.4	45.4	24.7	20.6	15.3	2.6	12.7
	[71.8,92.3]	[71.2,99.8]	[-25.8,0.4]	[27.5,52.5]	[41.9,90.8]	[-56.9,-9.8]	[33.3,58.1]	[7.5,57.0]	[-2.4,43.7]	[7.7,28.2]	[0.2,28.8]	[-0.4,25.8]
Guatemala	88.5	89.9	-1.4	43.0	63.9	-20.9	45.5	26.0	19.5	11.5	10.1	1.4
	[83.8,92.0]	[75.5,96.2]	[-11.5,8.8]	[37.4,48.7]	[51.1,74.9]	[-35.0,-6.9]	[39.3,52.0]	[17.3,37.1]	[5.8,33.3]	[8.0,16.2]	[3.8,24.5]	[-8.8,11.5]
Guyana	91.4	87.2	4.3	46.0	78.9	-32.9	45.5	8.3	37.2	8.6	12.8	-4.3
	[81.1,96.4]	[59.0,97.0]	[-12.9,21.4]	[30.4,62.4]	[55.9,91.7]	[-54.8,-11.0]	[30.8,60.9]	[2.4,24.8]	[20.5,53.9]	[3.6,18.9]	[3.0,41.0]	[-21.4,12.9]
Haiti	80.9	86.9	-6.1	29.6	53.5	-23.9	51.3	33.4	17.8	19.1	13.1	6.1
	[74.9,85.7]	[72.5,94.4]	[-15.0,2.9]	[23.4,36.6]	[37.9,68.4]	[-40.4,-7.3]	[45.5,57.0]	[19.8,50.6]	[1.1,34.5]	[14.3,25.1]	[5.6,27.5]	[-2.9,15.0]
Honduras	84.6	96.2	-11.6	48.8	61.9	-13.0	35.7	34.4	1.4	15.4	3.8	11.6
	[77.8,89.6]	[78.6,99.4]	[-20.0,-3.3]	[40.9,56.9]	[44.3,76.8]	[-28.8,2.7]	[29.5,42.5]	[21.6,49.9]	[-14.6,17.4]	[10.4,22.2]	[0.6,21.4]	[3.3,20.0]
Paraguay	95.8	NA	NA	41.3	82.6	-41.3	54.6	17.4	37.1	4.2	NA	NA
	[86.9,98.8]			[25.3,59.3]	[24.9,98.5]	[-87.6,5.0]	[38.4,69.8]	[1.5,75.1]	[-9.1,83.4]	[1.2,13.1]		
Peru	86.8	97.9	-11.1	45.1	39.7	5.4	41.7	58.2	-16.5	13.2	2.1	11.1
	[82.0,90.4]	[77.7,99.8]	[-19.0,-3.2]	[37.8,52.7]	[15.2,70.8]	[-26.1,37.0]	[34.4,49.4]	[26.3,84.4]	[-48.1,15.0]	[9.6,18.0]	[0.2,22.3]	[3.2,19.0]
Suriname	86.4	NA	NA	65.7	61.9	3.8	20.6	38.1	-17.4	13.6	NA	NA
	[74.2,93.3]			[45.7,81.3]	[23.8,89.5]	[-43.8,51.3]	[9.2,39.9]	[10.5,76.2]	[-61.4,26.6]	[6.7,25.8]		
Low-Income Countries	76.0	82.6	-6.6	30.5	40.9	-10.4	45.5	41.7	3.8	24.0	17.4	6.6
	[74.8,77.2]	[80.4,84.6]	[-8.6,-4.5]	[28.8,32.2]	[37.8,44.0]	[-14.0,-6.8]	[43.8,47.3]	[38.7,44.8]	[0.5,7.1]	[22.8,25.2]	[15.4,19.6]	[4.5,8.6]
Lower-Middle-Income Countries	79.9	90.1	-10.2	42.9	59.7	-16.9	37.0	30.3	6.7	20.1	9.9	10.2
	[78.7,81.0]	[88.9,91.1]	[-12.1,-8.3]	[41.5,44.2]	[57.9,61.6]	[-19.7,-14.0]	[35.8,38.3]	[28.5,32.2]	[4.3,9.1]	[19.0,21.3]	[8.9,11.1]	[8.3,12.1]
Upper-Middle-Income Countries	87.4	91.6	-4.2	43.0	55.6	-12.5	44.4	36.0	8.4	12.6	8.4	4.2
	[84.3,90.0]	[86.9,94.7]	[-8.8,0.4]	[39.2,46.9]	[47.9,62.9]	[-21.8,-3.2]	[40.2,48.6]	[29.6,43.1]	[-1.4,18.1]	[10.0,15.7]	[5.3,13.1]	[-0.4,8.8]

Notes: 95% confidence intervals are shown in brackets. 'Worst' refers to children in the 20% of households with the worst living standards and 'Best' refers to children in the 20% of households with the best living standards, within each country.

© 2022 Karlsson O et al. *JAMA Network Open*.

eTable 9. Mortality (Deaths per 1000) at Different Ages by Sex

	0–59 months			0–23 months			Neonates			1–23 months			24–59 months		
	Female	Male	Difference	Female	Male	Difference	Female	Male	Difference	Female	Male	Difference	Female	Male	Difference
Pooled	62.2	69.9	7.7	49.9	57.7	7.8	24.4	32.3	8.0	25.5	25.4	-0.2	12.3	12.2	-0.1
	[60.9,63.4]	[68.6,71.1]	[6.2,9.2]	[48.9,50.9]	[56.7,58.7]	[6.5,9.1]	[23.8,24.9]	[31.7,33.0]	[7.1,8.8]	[24.8,26.3]	[24.8,26.0]	[-0.9,0.6]	[11.7,12.9]	[11.7,12.7]	[-0.6,0.4]
Least Developed Countries	75.1	87.1	12.0	58.4	69.7	11.3	24.6	34.0	9.4	33.8	35.7	1.9	16.7	17.4	0.7
	[73.3,76.8]	[85.7,88.5]	[10.4,13.6]	[57.0,59.7]	[68.3,71.0]	[9.7,12.9]	[23.6,25.6]	[32.9,35.2]	[8.1,10.7]	[32.6,35.0]	[34.7,36.7]	[0.7,3.1]	[16.0,17.4]	[16.6,18.2]	[-0.3,1.7]
East Asia & Pacific	34.8	40.5	5.7	30.7	35.1	4.4	16.5	19.9	3.4	14.2	15.2	1.0	4.1	5.4	1.3
	[32.8,36.9]	[37.8,43.4]	[2.5,8.9]	[28.7,32.8]	[32.6,37.9]	[1.5,7.3]	[14.8,18.5]	[18.2,21.9]	[1.1,5.7]	[13.1,15.3]	[13.7,16.9]	[-1.0,3.0]	[3.4,4.9]	[4.6,6.2]	[0.1,2.4]
Cambodia	41.5	53.8	12.3	35.4	48.1	12.7	20.1	22.1	2.0	15.3	26.0	10.7	6.1	5.7	-0.4
	[34.9,49.3]	[47.0,61.4]	[4.2,20.4]	[29.0,43.0]	[42.0,55.0]	[5.4,20.0]	[16.3,24.6]	[18.2,26.8]	[-3.7,7.7]	[11.7,20.0]	[21.7,31.1]	[5.8,15.5]	[4.5,8.2]	[3.9,8.3]	[-3.2,2.4]
Indonesia	31.3	36.7	5.4	27.8	31.7	3.9	15.3	19.2	3.9	12.5	12.5	0.0	3.4	4.9	1.5
	[27.7,35.2]	[33.5,40.1]	[0.9,9.9]	[24.4,31.6]	[28.9,34.8]	[-0.4,8.3]	[12.9,18.2]	[17.3,21.4]	[0.7,7.0]	[10.4,14.9]	[10.6,14.7]	[-2.8,2.8]	[2.5,4.8]	[3.6,6.8]	[-0.3,3.2]
Lao	45.4	53.0	7.6	40.9	48.9	8.0	19.4	21.2	1.9	21.5	27.7	6.2	4.5	4.1	-0.4
	[40.9,50.3]	[48.0,58.5]	[1.9,13.3]	[36.3,46.0]	[43.9,54.5]	[2.4,13.7]	[16.6,22.6]	[18.1,24.9]	[-2.6,6.3]	[18.1,25.5]	[23.9,32.1]	[1.5,10.9]	[3.4,5.9]	[3.1,5.4]	[-2.1,1.2]
Mongolia	15.6	20.7	5.2	13.6	16.7	3.1	6.3	9.6	3.2	7.3	7.1	-0.2	1.9	4.0	2.1
	[11.8,20.5]	[16.5,26.1]	[-1.0,11.4]	[10.2,18.1]	[12.6,22.1]	[-2.8,8.9]	[4.1,9.7]	[6.7,13.7]	[-1.2,7.7]	[4.9,10.8]	[4.5,11.3]	[-5.0,4.6]	[0.8,4.4]	[2.2,7.3]	[-0.7,4.9]
Myanmar	65.6	77.9	12.3	59.1	67.1	8.0	29.3	34.4	5.1	29.8	32.7	3.0	6.5	10.7	4.3
	[57.1,75.1]	[67.8,89.2]	[1.4,23.1]	[51.4,67.9]	[58.1,77.5]	[-2.6,18.6]	[24.0,35.8]	[28.5,41.5]	[-3.4,13.6]	[24.0,37.0]	[26.0,41.2]	[-5.2,11.1]	[4.3,9.6]	[7.7,14.9]	[-0.1,8.6]
Papua New Guinea	50.3	53.3	3.0	42.0	41.8	-0.2	21.4	25.8	4.5	20.6	16.0	-4.6	8.3	11.5	3.1
	[43.5,58.2]	[46.3,61.3]	[-5.7,11.6]	[36.1,48.7]	[35.5,49.2]	[-7.4,7.1]	[17.1,26.6]	[21.6,30.9]	[-0.5,9.5]	[16.8,25.2]	[11.3,22.5]	[-12.4,3.2]	[6.0,11.6]	[8.8,15.0]	[-1.0,7.3]
Philippines	26.3	29.4	3.1	22.6	26.0	3.4	12.7	14.8	2.0	9.9	11.2	1.3	3.7	3.5	-0.3
	[22.3,31.0]	[24.9,34.7]	[-3.0,9.3]	[18.6,27.5]	[21.7,31.0]	[-2.7,9.4]	[10.2,15.9]	[11.6,18.8]	[-2.4,6.5]	[7.2,13.5]	[8.9,14.1]	[-2.3,4.9]	[2.6,5.3]	[2.4,5.0]	[-2.3,1.8]
Timor-Leste	35.7	45.1	9.4	28.8	36.0	7.2	12.7	20.8	8.1	16.1	15.3	-0.8	6.9	9.1	2.2
	[31.0,41.2]	[39.1,52.1]	[1.1,17.8]	[25.1,33.1]	[30.7,42.2]	[-0.1,14.6]	[9.7,16.6]	[16.6,25.9]	[2.5,13.7]	[13.4,19.4]	[11.8,19.8]	[-6.3,4.6]	[4.9,9.6]	[6.5,12.8]	[-1.6,6.0]
Tonga	10.1	12.3	2.2	10.1	10.8	0.7	5.2	5.0	-0.2	4.8	5.8	1.0	NA	1.5	NA
	[6.0,16.8]	[7.1,21.2]	[-6.6,11.0]	[6.0,16.8]	[5.9,19.7]	[-7.6,9.1]	[2.7,10.3]	[2.3,10.7]	[-6.5,6.0]	[1.7,13.7]	[2.2,15.1]	[-7.2,9.1]		[0.4,5.3]	
South Asia	51.8	57.6	5.8	45.4	52.3	6.9	26.9	36.1	9.2	18.6	16.2	-2.4	6.4	5.3	-1.1
	[50.0,53.7]	[55.8,59.4]	[3.9,7.7]	[43.8,47.2]	[50.6,54.0]	[5.1,8.6]	[25.8,28.0]	[35.0,37.2]	[7.8,10.7]	[17.5,19.7]	[15.2,17.3]	[-3.4,-1.3]	[6.0,6.8]	[5.0,5.6]	[-1.6,-0.5]
Afghanistan	58.0	65.8	7.9	52.0	58.7	6.7	21.0	28.4	7.4	31.0	30.3	-0.7	5.9	7.1	1.2
	[52.6,63.8]	[58.9,73.4]	[0.6,15.1]	[47.4,57.1]	[51.5,66.9]	[-1.8,15.2]	[18.5,24.0]	[25.2,32.1]	[3.5,11.4]	[27.0,35.6]	[25.6,35.8]	[-6.9,5.5]	[4.3,8.3]	[5.5,9.1]	[-2.0,4.3]
Bangladesh	36.6	44.3	7.7	32.3	39.7	7.4	22.1	29.5	7.5	10.2	10.1	-0.0	4.3	4.6	0.3
	[34.0,39.3]	[41.4,47.3]	[3.6,11.8]	[29.8,34.9]	[37.0,42.6]	[3.6,11.2]	[20.2,24.1]	[27.3,32.0]	[4.6,10.3]	[8.6,12.0]	[8.6,11.8]	[-2.1,2.0]	[3.4,5.5]	[3.7,5.7]	[-1.3,1.8]
India	50.4	53.8	3.4	44.0	48.5	4.5	26.6	34.0	7.4	17.4	14.5	-2.9	6.4	5.3	-1.1
	[49.4,51.4]	[52.4,55.2]	[2.0,4.8]	[43.2,44.9]	[47.2,49.9]	[3.0,6.0]	[26.0,27.3]	[32.9,35.2]	[6.2,8.5]	[16.7,18.0]	[13.8,15.3]	[-3.7,-2.0]	[6.0,6.8]	[4.9,5.7]	[-1.7,-0.5]
Maldives	17.8	25.1	7.2	17.0	21.6	4.6	10.2	14.9	4.7	6.8	6.7	-0.1	0.9	3.5	2.7
	[13.5,23.6]	[18.9,33.2]	[-1.0,15.4]	[12.6,22.8]	[15.6,29.8]	[-3.6,12.8]	[6.8,15.1]	[9.7,22.8]	[-3.6,13.0]	[3.8,12.1]	[3.4,13.1]	[-3.8,3.6]	[0.3,2.5]	[1.7,7.2]	[0.0,5.3]
Nepal	30.9	34.6	3.7	26.2	32.1	5.9	11.6	23.1	11.5	14.6	8.9	-5.6	4.7	2.6	-2.1
	[26.5,36.0]	[29.4,40.7]	[-2.3,9.8]	[21.7,31.5]	[27.0,38.0]	[-0.3,12.0]	[8.6,15.6]	[19.2,27.8]	[6.4,16.6]	[11.4,18.6]	[6.6,12.0]	[-9.8,-1.5]	[2.6,8.5]	[1.5,4.3]	[-5.1,0.8]
Pakistan	66.2	82.0	15.9	58.5	76.3	17.8	32.8	51.7	18.8	25.6	24.6	-1.0	7.7	5.7	-2.0
	[58.1,75.3]	[73.5,91.5]	[4.8,27.0]	[51.4,66.4]	[68.1,85.4]	[7.7,27.9]	[27.8,38.7]	[46.6,57.3]	[11.6,26.0]	[21.4,30.6]	[19.6,31.0]	[-7.2,5.2]	[5.8,10.2]	[4.4,7.5]	[-4.6,0.6]
Europe & Central Asia	22.6	22.8	0.2	20.9	20.6	-0.3	10.5	11.6	1.1	10.3	9.0	-1.3	1.7	2.2	0.5
	[18.5,27.6]	[19.7,26.3]	[-5.8,6.3]	[16.9,25.9]	[17.7,24.0]	[-6.3,5.8]	[8.0,13.9]	[10.0,13.5]	[-2.4,4.6]	[7.6,14.0]	[6.9,11.7]	[-5.4,2.8]	[1.1,2.7]	[1.4,3.4]	[-0.6,1.6]
Albania	3.7	7.8	4.0	3.5	7.8	4.3	2.0	4.7	2.6	1.5	3.1	1.6	0.3	NA	NA
	[1.7,8.0]	[4.1,14.6]	[-1.1,9.1]	[1.5,8.0]	[4.1,14.6]	[-0.8,9.3]	[0.6,6.8]	[2.5,8.6]	[-1.6,6.9]	[0.4,5.1]	[1.0,9.2]	[-1.3,4.5]	[0.0,1.5]		
Armenia	11.3	9.8	-1.5	11.1	8.3	-2.9	6.0	4.7	-1.2	5.2	3.5	-1.6	0.2	1.5	1.4
	[7.2,17.7]	[6.3,15.3]	[-8.3,5.3]	[7.1,17.5]	[4.9,13.8]	[-9.7,4.0]	[3.4,10.4]	[2.4,9.5]	[-6.0,3.6]	[2.4,11.0]	[1.5,8.3]	[-7.3,4.0]	[0.0,0.7]	[0.6,4.2]	[-0.4,3.2]

© 2022 Karlsson O et al. *JAMA Network Open*.

Kyrgyz Republic	19.6	24.7	5.0	18.9	22.1	3.2	12.6	15.6	3.0	6.4	6.5	0.1	0.7	2.6	1.9
	[14.1,27.3]	[19.4,31.3]	[-3.7,13.8]	[13.4,26.8]	[16.9,28.9]	[-5.5,11.8]	[8.7,18.1]	[10.9,22.3]	[-3.9,10.0]	[3.4,12.0]	[3.8,11.0]	[-4.3,4.6]	[0.3,1.7]	[1.2,5.7]	[-0.2,4.0]
Moldova	15.4	16.1	0.7	15.1	14.6	-0.5	11.5	12.2	0.7	3.6	2.4	-1.2	0.3	1.4	1.2
	[10.0,23.6]	[9.8,26.3]	[-8.5,9.9]	[9.8,23.3]	[8.9,24.0]	[-10.3,9.3]	[7.0,18.8]	[7.6,19.5]	[-7.4,8.7]	[1.4,9.1]	[0.6,9.5]	[-5.8,3.5]	[0.1,1.3]	[0.1,16.1]	[-1.2,3.5]
Tajikistan	26.9	39.6	12.7	24.4	35.9	11.6	10.9	18.7	7.7	13.4	17.3	3.8	2.5	3.6	1.1
	[22.9,31.5]	[34.5,45.4]	[5.3,20.2]	[21.0,28.3]	[31.3,41.2]	[4.9,18.2]	[8.4,14.2]	[15.3,22.7]	[3.3,12.2]	[10.9,16.5]	[13.1,22.7]	[-2.0,9.7]	[1.1,5.5]	[2.0,6.5]	[-1.9,4.2]
Turkey	22.7	17.6	-5.1	21.1	16.0	-5.1	10.1	8.5	-1.6	11.0	7.5	-3.5	1.6	1.6	0.0
	[16.8,30.6]	[13.1,23.7]	[-14.2,4.0]	[15.2,29.3]	[11.8,21.6]	[-14.0,3.8]	[6.4,16.0]	[6.0,12.1]	[-7.1,3.8]	[7.2,16.7]	[4.6,12.1]	[-9.9,2.9]	[0.7,3.7]	[0.6,4.1]	[-1.4,1.4]
Turkmenistan	26.6	44.1	17.4	23.3	38.8	15.5	14.0	24.1	10.2	9.3	14.7	5.4	3.4	5.3	1.9
	[20.9,33.9]	[36.7,52.9]	[7.3,27.6]	[18.0,30.0]	[32.2,46.6]	[6.3,24.8]	[9.8,19.8]	[19.2,30.2]	[3.0,17.4]	[6.0,14.5]	[10.6,20.3]	[-0.8,11.6]	[1.7,6.8]	[3.3,8.5]	[-1.6,5.4]
Middle East & North Africa	30.9	33.1	2.2	28.3	30.2	1.9	14.8	18.1	3.3	13.5	12.1	-1.4	2.6	3.0	0.4
	[28.5,33.5]	[31.0,35.4]	[-0.2,4.7]	[26.0,30.8]	[28.2,32.3]	[-0.6,4.4]	[13.5,16.2]	[16.6,19.7]	[1.5,5.1]	[12.2,15.0]	[11.0,13.4]	[-3.3,0.4]	[2.2,3.1]	[2.4,3.7]	[-0.5,1.2]
Egypt	30.1	30.4	0.3	27.9	27.5	-0.4	15.0	17.5	2.5	12.9	10.0	-2.9	2.2	2.9	0.7
	[27.2,33.3]	[27.6,33.5]	[-3.9,4.5]	[25.1,31.1]	[24.8,30.5]	[-4.3,3.4]	[12.7,17.7]	[15.3,20.0]	[-0.2,5.1]	[11.1,15.0]	[8.6,11.6]	[-5.7,-0.1]	[1.5,3.1]	[1.9,4.5]	[-0.8,2.3]
Iraq	21.9	26.8	4.9	19.8	24.3	4.5	11.6	14.1	2.5	8.2	10.2	2.0	2.1	2.5	0.4
	[18.3,26.2]	[22.5,31.9]	[-0.6,10.5]	[16.4,23.9]	[20.3,29.1]	[-1.0,10.0]	[9.3,14.4]	[11.4,17.4]	[-1.1,6.1]	[6.4,10.5]	[8.1,12.8]	[-1.0,5.0]	[1.4,3.4]	[1.8,3.5]	[-0.6,1.4]
Jordan	13.9	19.4	5.5	12.9	17.7	4.8	6.7	11.6	4.9	6.2	6.1	-0.1	0.9	1.7	0.7
	[10.8,17.7]	[15.5,24.3]	[0.5,10.6]	[10.0,16.7]	[14.1,22.3]	[0.1,9.6]	[5.0,9.0]	[8.6,15.7]	[1.1,8.8]	[4.1,9.5]	[4.5,8.3]	[-2.7,2.5]	[0.4,2.3]	[0.9,2.9]	[-0.5,1.9]
State of Palestine	20.3	25.3	5.0	18.5	22.8	4.4	9.8	13.1	3.3	8.7	9.7	1.0	1.8	2.5	0.6
	[17.3,23.8]	[22.1,28.9]	[-0.8,10.8]	[15.6,21.9]	[20.1,25.8]	[-1.2,10.0]	[8.1,11.7]	[10.6,16.2]	[-0.9,7.5]	[6.6,11.5]	[7.7,12.3]	[-2.4,4.4]	[1.0,3.5]	[1.4,4.2]	[-0.8,2.1]
Tunisia	15.0	16.6	1.6	13.9	15.2	1.3	9.7	9.2	-0.5	4.2	6.0	1.8	1.1	1.4	0.3
	[10.8,20.8]	[12.8,21.6]	[-4.9,8.1]	[9.9,19.5]	[11.5,20.1]	[-4.6,7.3]	[6.4,14.6]	[6.7,12.7]	[-4.6,3.7]	[2.1,8.2]	[3.9,9.0]	[-2.6,6.1]	[0.3,3.6]	[0.7,2.9]	[-1.5,2.1]
Yemen	55.7	59.3	3.5	50.4	54.7	4.3	22.7	30.2	7.5	27.7	24.5	-3.2	5.3	4.6	-0.7
	[51.1,60.7]	[54.0,65.1]	[-2.3,9.3]	[46.2,55.0]	[49.9,59.9]	[-1.1,9.6]	[20.0,25.8]	[26.8,34.0]	[3.2,11.8]	[24.4,31.5]	[21.1,28.4]	[-8.1,1.7]	[4.1,6.9]	[3.3,6.4]	[-2.2,0.7]
Eastern & Southern Africa	66.6	80.8	14.2	53.5	67.2	13.7	23.5	34.5	11.0	30.0	32.8	2.7	13.1	13.5	0.5
	[64.0,69.3]	[78.2,83.4]	[10.9,17.5]	[51.1,56.1]	[65.0,69.5]	[10.5,17.0]	[22.0,25.1]	[32.7,36.3]	[8.4,13.5]	[28.5,31.6]	[31.2,34.5]	[1.1,4.4]	[12.1,14.1]	[12.4,14.8]	[-0.7,1.6]
Angola	72.5	86.8	14.3	52.9	66.7	13.8	18.8	30.0	11.3	34.1	36.6	2.5	19.7	20.2	0.5
	[65.0,80.9]	[77.5,97.1]	[5.0,23.5]	[47.1,59.3]	[59.0,75.3]	[6.0,21.6]	[15.4,22.9]	[25.2,35.8]	[5.0,17.6]	[30.0,38.8]	[31.4,42.7]	[-2.9,7.9]	[16.5,23.5]	[16.7,24.3]	[-3.2,4.1]
Burundi	77.9	86.0	8.1	58.1	66.3	8.2	23.2	26.7	3.5	34.9	39.6	4.7	19.9	19.7	-0.2
	[72.3,84.0]	[80.9,91.4]	[-0.1,16.2]	[52.8,63.9]	[61.0,72.1]	[0.3,16.2]	[20.0,27.0]	[23.1,30.9]	[-1.4,8.4]	[30.9,39.3]	[36.4,43.1]	[-1.1,10.6]	[17.0,23.1]	[17.4,22.3]	[-3.8,3.5]
Comoros	51.6	48.1	-3.5	40.4	43.0	2.6	22.8	27.9	5.1	17.6	15.1	-2.4	11.2	5.1	-6.1
	[41.4,64.0]	[38.5,60.0]	[-17.5,10.6]	[31.2,52.2]	[34.3,53.8]	[-11.0,16.3]	[16.0,32.4]	[21.6,36.0]	[-5.6,15.8]	[11.9,25.8]	[10.8,21.0]	[-9.7,4.8]	[7.5,16.5]	[3.0,8.7]	[-10.6,-1.6]
Ethiopia	67.7	94.2	26.6	54.4	81.4	27.0	26.4	48.7	22.3	28.0	32.7	4.7	13.2	12.8	-0.4
	[59.7,76.6]	[86.4,102.7]	[18.0,35.2]	[47.3,62.6]	[74.8,88.6]	[18.6,35.4]	[21.4,32.7]	[43.6,54.4]	[15.3,29.3]	[23.2,33.7]	[28.0,38.1]	[-0.8,10.2]	[10.6,16.4]	[9.7,17.0]	[-4.7,3.9]
Kenya	52.4	59.6	7.2	43.9	49.8	5.9	20.7	25.1	4.4	23.2	24.7	1.5	8.5	9.7	1.2
	[48.0,57.2]	[55.7,63.7]	[1.5,12.8]	[39.6,48.6]	[46.0,53.9]	[0.2,11.7]	[18.0,23.8]	[22.1,28.5]	[0.8,8.0]	[19.9,26.9]	[22.0,27.7]	[-2.4,5.5]	[7.1,10.3]	[7.9,12.0]	[-0.9,3.4]
Lesotho	74.5	98.0	23.5	64.1	89.4	25.3	37.5	45.4	7.9	26.6	44.0	17.4	10.4	8.6	-1.8
	[63.4,87.4]	[83.1,115.3]	[6.8,40.2]	[53.8,76.2]	[76.2,104.6]	[9.3,41.3]	[28.7,48.8]	[35.7,57.5]	[-4.1,19.8]	[19.3,36.5]	[36.0,53.8]	[5.9,29.0]	[5.9,18.4]	[4.6,16.1]	[-9.0,5.3]
Madagascar	54.7	65.4	10.7	40.8	49.0	8.1	19.7	23.6	4.0	21.2	25.4	4.2	13.8	16.4	2.6
	[49.9,59.9]	[59.5,71.9]	[3.4,18.1]	[36.9,45.2]	[44.3,54.1]	[2.1,14.2]	[16.9,22.8]	[20.6,27.0]	[-0.3,8.2]	[18.5,24.2]	[22.0,29.3]	[0.7,7.7]	[11.5,16.6]	[13.9,19.5]	[-1.0,6.2]
Malawi	64.7	84.8	20.1	48.6	65.9	17.2	20.5	32.5	12.0	28.1	33.4	5.3	16.1	19.0	2.8
	[59.9,69.9]	[79.7,90.3]	[13.7,26.5]	[44.3,53.3]	[61.6,70.3]	[11.6,22.9]	[17.6,24.0]	[29.4,35.9]	[8.5,15.5]	[25.4,31.0]	[30.4,36.6]	[1.1,9.4]	[14.0,18.6]	[16.1,22.4]	[-0.2,5.9]
Mozambique	102.8	113.5	10.7	81.4	89.3	7.9	27.2	36.7	9.5	54.2	52.6	-1.6	21.3	24.1	2.8
	[94.4,111.8]	[106.3,121.1]	[1.2,20.2]	[74.6,88.8]	[82.8,96.4]	[-0.4,16.3]	[23.5,31.5]	[32.1,42.0]	[3.4,15.6]	[48.4,60.6]	[47.3,58.4]	[-9.7,6.4]	[17.5,26.0]	[20.4,28.5]	[-2.8,8.4]
Namibia	53.8	64.0	10.2	44.3	52.6	8.2	14.2	23.4	9.2	30.2	29.2	-1.0	9.5	11.5	2.0
	[47.7,60.6]	[55.4,74.0]	[0.6,19.9]	[38.3,51.2]	[45.5,60.6]	[-1.0,17.4]	[10.8,18.6]	[19.0,28.7]	[3.7,14.6]	[25.1,36.2]	[23.7,35.9]	[-8.7,6.8]	[6.8,13.1]	[8.3,15.8]	[-3.3,7.3]
Rwanda	64.0	70.2	6.2	49.7	53.9	4.2	19.8	24.6	4.8	29.8	29.3	-0.5	14.3	16.3	1.9
	[58.3,70.2]	[63.9,77.0]	[-1.5,13.8]	[44.9,54.9]	[48.7,59.6]	[-2.2,10.6]	[17.0,23.2]	[21.5,28.1]	[0.6,8.9]	[26.4,33.6]	[25.4,33.7]	[-5.5,4.4]	[11.8,17.4]	[13.8,19.2]	[-1.8,5.7]

© 2022 Karlsson O et al. *JAMA Network Open*.

South Africa	44.1	56.4	12.3	41.1	51.8	10.7	20.0	28.9	9.0	21.1	22.9	1.7	3.0	4.6	1.5
	[35.0,55.5]	[46.7,67.9]	[-1.4,26.0]	[31.9,52.7]	[42.2,63.4]	[-2.8,24.2]	[12.6,31.4]	[21.2,39.4]	[-2.7,20.7]	[14.7,30.2]	[17.7,29.4]	[-7.3,10.7]	[1.7,5.4]	[2.9,7.4]	[-1.2,4.3]
South Sudan	87.5	105.2	17.7	71.5	86.1	14.7	31.3	40.2	8.9	40.2	45.9	5.7	16.0	19.1	3.1
	[79.5,96.2]	[94.8,116.7]	[7.6,27.9]	[64.8,78.8]	[77.2,96.0]	[5.6,23.7]	[26.5,37.0]	[34.5,47.0]	[3.7,14.2]	[34.0,47.4]	[40.5,51.9]	[-0.5,11.9]	[13.0,19.8]	[15.8,23.2]	[-0.8,6.9]
Sudan	60.7	76.7	16.0	52.8	66.4	13.6	24.8	35.8	11.0	28.0	30.6	2.6	8.0	10.3	2.4
	[56.0,65.9]	[71.2,82.7]	[8.3,23.8]	[48.2,57.7]	[61.1,72.1]	[5.5,21.7]	[21.7,28.3]	[32.5,39.4]	[6.6,15.3]	[24.4,32.0]	[26.9,34.7]	[-3.4,8.7]	[6.2,10.2]	[8.4,12.7]	[-0.5,5.3]
Swaziland	69.4	89.3	19.9	62.4	75.4	13.0	17.6	19.3	1.7	44.8	56.1	11.3	7.0	13.8	6.8
	[57.6,83.5]	[71.4,111.1]	[2.0,37.7]	[51.3,75.7]	[61.0,93.0]	[-3.6,29.7]	[12.9,23.9]	[14.2,26.3]	[-5.8,9.3]	[34.6,57.8]	[42.5,73.7]	[-4.2,26.9]	[4.2,11.8]	[8.2,23.2]	[-0.9,14.6]
Tanzania	75.9	80.1	4.2	59.6	69.6	10.0	25.2	32.7	7.5	34.4	36.9	2.4	16.3	10.5	-5.8
	[68.8,83.6]	[72.5,88.4]	[-6.3,14.7]	[53.6,66.3]	[63.0,76.8]	[0.8,19.1]	[21.5,29.6]	[28.6,37.4]	[1.4,13.6]	[29.9,39.6]	[32.4,41.8]	[-3.4,8.3]	[13.0,20.3]	[8.2,13.5]	[-10.2,-1.3]
Uganda	65.6	80.8	15.1	52.5	65.4	12.9	23.5	31.3	7.8	29.1	34.1	5.1	13.1	15.3	2.2
	[60.7,71.0]	[75.3,86.5]	[9.2,21.0]	[48.1,57.4]	[61.1,70.1]	[7.8,18.0]	[21.1,26.1]	[28.3,34.6]	[4.2,11.5]	[25.8,32.7]	[31.1,37.5]	[0.8,9.3]	[11.0,15.6]	[12.8,18.3]	[-1.5,5.9]
Zambia	58.8	69.7	11.0	47.4	55.3	7.9	20.0	30.0	10.0	27.4	25.4	-2.1	11.4	14.4	3.1
	[52.3,66.0]	[61.8,78.7]	[2.6,19.3]	[42.0,53.6]	[48.0,63.7]	[-0.6,16.4]	[16.2,24.7]	[24.3,36.9]	[1.6,18.3]	[23.2,32.4]	[22.2,28.9]	[-6.8,2.7]	[8.9,14.5]	[11.9,17.5]	[-0.8,7.0]
Zimbabwe	67.8	77.7	9.9	56.4	65.9	9.5	27.3	35.7	8.4	29.2	30.3	1.1	11.3	11.7	0.4
	[60.1,76.3]	[68.5,87.9]	[0.0,19.8]	[50.4,63.1]	[58.7,73.9]	[0.6,18.4]	[23.4,31.7]	[30.1,42.2]	[2.0,14.8]	[24.4,34.8]	[25.4,35.9]	[-5.0,7.2]	[7.9,16.2]	[8.7,15.8]	[-4.1,5.0]
West & Central Africa	110.1	121.6	11.6	76.8	86.9	10.0	29.5	37.4	7.9	47.3	49.5	2.2	33.2	34.8	1.5
	[106.5,113.8]	[118.5,124.8]	[7.9,15.2]	[74.2,79.5]	[84.4,89.5]	[7.3,12.8]	[28.0,31.1]	[36.1,38.8]	[6.1,9.6]	[45.4,49.3]	[47.6,51.4]	[0.3,4.1]	[31.6,35.0]	[33.1,36.5]	[-0.3,3.3]
Benin	93.4	110.5	17.1	66.2	81.2	15.0	25.8	39.1	13.3	40.3	42.1	1.7	27.2	29.4	2.2
	[86.3,101.0]	[103.3,118.2]	[8.5,25.8]	[60.5,72.4]	[75.3,87.4]	[8.1,21.9]	[22.9,29.2]	[34.7,44.0]	[8.3,18.2]	[36.0,45.2]	[37.9,46.7]	[-3.9,7.3]	[23.8,31.1]	[26.0,33.2]	[-2.3,6.6]
Burkina Faso	141.3	153.7	12.4	100.1	110.8	10.8	30.8	37.7	6.9	69.2	73.2	3.9	41.3	42.9	1.7
	[134.4,148.5]	[143.9,164.2]	[3.7,21.1]	[94.5,105.9]	[103.7,118.4]	[4.0,17.6]	[27.8,34.2]	[33.8,42.0]	[2.2,11.5]	[64.8,74.0]	[67.4,79.3]	[-1.8,9.7]	[38.2,44.5]	[38.5,47.9]	[-3.3,6.7]
Cameroon	80.5	98.2	17.7	57.8	72.0	14.3	25.7	33.6	7.9	32.1	38.4	6.3	22.7	26.1	3.4
	[73.6,88.0]	[88.9,108.3]	[7.2,28.1]	[52.4,63.7]	[65.0,79.7]	[5.9,22.6]	[22.2,29.6]	[29.0,38.9]	[2.5,13.4]	[27.4,37.6]	[33.8,43.7]	[-0.3,12.9]	[19.2,26.9]	[22.0,31.0]	[-2.2,9.0]
Chad	140.1	155.0	14.9	99.8	115.1	15.3	29.5	42.6	13.1	70.3	72.6	2.2	40.3	39.9	-0.4
	[127.6,153.6]	[141.7,169.4]	[6.5,23.4]	[87.3,113.9]	[101.0,130.9]	[7.3,23.3]	[25.5,34.0]	[38.0,47.7]	[8.5,17.7]	[60.5,81.7]	[62.1,84.6]	[-4.0,8.5]	[36.9,44.1]	[36.7,43.5]	[-5.5,4.7]
Congo	77.5	85.9	8.3	59.9	59.1	-0.8	21.3	27.4	6.1	38.7	31.7	-6.9	17.6	26.8	9.1
	[69.5,86.4]	[77.5,95.1]	[-1.1,17.8]	[52.1,68.8]	[52.2,66.9]	[-10.4,8.8]	[16.6,27.1]	[22.3,33.6]	[-0.9,13.1]	[31.9,46.7]	[27.3,36.9]	[-15.6,1.7]	[13.8,22.5]	[22.3,32.1]	[2.5,15.8]
Congo (DR)	108.3	114.9	6.5	79.8	83.9	4.1	28.1	32.2	4.1	51.7	51.7	0.0	28.6	31.0	2.4
	[100.2,117.1]	[107.9,122.3]	[-1.5,14.5]	[73.1,87.0]	[78.5,89.6]	[-3.7,11.9]	[23.8,33.2]	[28.5,36.3]	[-1.6,9.8]	[46.7,57.1]	[47.1,56.7]	[-6.0,6.0]	[25.6,31.8]	[26.9,35.6]	[-1.6,6.5]
Cote D'Ivoire	91.1	110.8	19.8	71.8	86.8	15.0	30.5	40.2	9.7	41.3	46.6	5.2	19.2	24.0	4.8
	[82.4,100.5]	[101.7,120.7]	[10.0,29.5]	[64.0,80.5]	[78.5,95.8]	[5.9,24.0]	[25.8,36.0]	[36.3,44.6]	[3.1,16.4]	[35.6,48.0]	[40.0,54.1]	[-1.1,11.5]	[16.2,22.9]	[19.3,30.0]	[-0.4,10.0]
Gabon	56.3	70.5	14.2	45.7	54.9	9.2	23.7	27.0	3.3	22.0	27.9	5.9	10.7	15.6	4.9
	[46.6,67.9]	[60.1,82.6]	[2.9,25.5]	[36.8,56.6]	[45.7,65.8]	[-2.7,21.2]	[16.9,33.1]	[20.6,35.3]	[-5.4,12.0]	[16.8,28.8]	[21.6,36.1]	[-1.4,13.3]	[7.3,15.6]	[11.5,21.1]	[-2.0,11.9]
Gambia	51.2	66.8	15.7	40.8	55.2	14.4	23.0	35.3	12.3	17.8	19.9	2.1	10.4	11.6	1.2
	[45.4,57.6]	[60.3,74.0]	[7.7,23.6]	[35.9,46.3]	[49.9,61.1]	[7.6,21.3]	[19.2,27.5]	[31.5,39.7]	[6.7,18.0]	[14.9,21.3]	[16.3,24.3]	[-2.7,6.9]	[7.9,13.6]	[9.1,14.7]	[-2.7,5.2]
Ghana	62.2	77.6	15.5	49.9	59.9	10.1	26.8	34.7	7.9	23.0	25.2	2.1	12.3	17.7	5.4
	[55.8,69.2]	[69.5,86.7]	[7.2,23.8]	[44.2,56.2]	[52.9,67.9]	[0.7,19.4]	[21.1,34.1]	[29.4,41.0]	[-0.0,15.9]	[19.5,27.2]	[20.6,30.8]	[-3.6,7.9]	[9.5,15.8]	[13.0,24.1]	[0.1,10.7]
Guinea	100.6	115.9	15.3	73.5	86.6	13.1	28.5	38.0	9.5	45.0	48.6	3.6	27.1	29.3	2.2
	[90.2,111.9]	[105.0,127.7]	[4.5,26.1]	[65.2,82.7]	[77.5,96.7]	[3.2,23.1]	[24.7,32.8]	[32.5,44.4]	[3.4,15.7]	[38.3,52.8]	[41.6,56.7]	[-4.1,11.3]	[22.9,32.1]	[25.3,33.8]	[-3.6,8.0]
Liberia	111.3	114.5	3.3	86.8	91.6	4.8	30.1	37.2	7.2	56.7	54.4	-2.3	24.5	22.9	-1.6
	[99.3,124.5]	[102.4,127.9]	[-8.1,14.6]	[78.7,95.6]	[80.3,104.4]	[-5.6,15.3]	[24.1,37.4]	[30.7,45.1]	[-0.0,14.3]	[49.8,64.5]	[47.0,62.9]	[-12.5,7.8]	[18.6,32.1]	[20.0,26.1]	[-8.0,4.8]
Mali	105.3	120.0	14.7	70.4	82.0	11.6	30.9	37.7	6.8	39.5	44.3	4.8	34.9	38.0	3.1
	[97.1,114.2]	[107.7,133.5]	[3.1,26.2]	[63.3,78.3]	[73.4,91.6]	[2.3,20.8]	[26.0,36.7]	[32.5,43.8]	[1.9,11.7]	[35.0,44.6]	[38.2,51.3]	[-2.3,11.9]	[30.7,39.6]	[32.8,44.0]	[-3.1,9.3]
Mauritania	48.4	56.8	8.3	40.1	48.7	8.7	23.5	31.5	8.0	16.5	17.2	0.7	8.4	8.1	-0.3
	[43.3,54.2]	[50.0,64.4]	[0.9,15.7]	[35.1,45.7]	[42.8,55.5]	[1.5,15.8]	[20.0,27.7]	[26.8,37.0]	[2.5,13.4]	[13.5,20.3]	[14.4,20.6]	[-3.7,5.1]	[6.7,10.6]	[5.9,11.1]	[-3.7,3.0]
Niger	146.0	159.8	13.7	90.3	104.4	14.1	24.7	38.1	13.4	65.6	66.3	0.7	55.8	55.4	-0.3
	[138.0,154.5]	[151.3,168.6]	[2.9,24.6]	[84.6,96.4]	[97.5,111.7]	[6.0,22.1]	[22.1,27.6]	[34.2,42.4]	[9.2,17.6]	[60.3,71.3]	[60.3,72.8]	[-5.3,6.7]	[51.0,61.0]	[50.2,61.1]	[-7.4,6.7]
Nigeria	123.8	133.5	9.7	82.8	92.7	9.9	32.8	40.8	8.0	50.0	52.0	1.9	41.0	40.8	-0.2
	[115.7,132.5]	[126.6,140.8]	[2.6,16.8]	[76.7,89.4]	[88.0,97.7]	[3.7,16.1]	[28.9,37.2]	[37.7,44.0]	[4.3,11.6]	[46.5,53.8]	[48.4,55.7]	[-2.3,6.1]	[37.8,44.5]	[37.0,45.0]	[-3.7,3.3]

© 2022 Karlsson O et al. *JAMA Network Open*.

Sao Tome & Principe	35.1	50.4	15.3	31.3	39.8	8.5	12.4	23.4	11.0	18.9	16.4	-2.5	3.8	10.6	6.7
	[25.5,48.1]	[36.7,68.8]	[-6.4,37.0]	[23.0,42.5]	[28.3,55.9]	[-10.1,27.2]	[7.9,19.3]	[15.6,34.9]	[0.5,21.5]	[12.4,28.8]	[10.5,25.7]	[-14.8,9.9]	[1.4,10.2]	[6.6,16.8]	[0.8,12.7]
Senegal	44.0	52.8	8.8	36.2	44.9	8.7	20.8	27.0	6.2	15.3	17.9	2.6	7.8	7.9	0.1
	[37.0,52.3]	[46.5,60.0]	[-0.7,18.4]	[30.1,43.4]	[38.6,52.3]	[-0.8,18.3]	[16.1,26.9]	[22.9,31.8]	[-0.9,13.3]	[12.5,18.8]	[13.9,23.0]	[-3.3,8.4]	[5.5,11.1]	[5.9,10.7]	[-2.9,3.1]
Sierra Leone	113.1	135.1	22.0	88.1	104.1	16.0	24.0	35.1	11.2	64.2	69.0	4.8	25.0	31.0	6.0
	[105.1,121.7]	[126.9,143.7]	[12.1,31.9]	[81.5,95.2]	[96.6,112.2]	[8.1,23.9]	[20.2,28.4]	[30.9,39.9]	[5.8,16.5]	[58.8,69.9]	[63.1,75.4]	[-2.0,11.7]	[21.1,29.6]	[25.7,37.3]	[-1.9,13.9]
Togo	67.6	81.1	13.6	47.7	54.1	6.5	25.7	30.0	4.3	21.9	24.1	2.2	19.9	27.0	7.1
	[58.1,78.5]	[72.2,91.1]	[-0.2,27.3]	[39.3,57.6]	[47.6,61.6]	[-4.8,17.8]	[20.0,33.0]	[25.0,36.0]	[-3.4,12.1]	[16.6,28.9]	[19.0,30.6]	[-7.3,11.6]	[15.2,26.0]	[21.5,33.9]	[-0.1,14.3]
Latin America & Caribbean	30.0	33.9	3.9	26.5	30.5	4.0	14.1	16.7	2.6	12.4	13.8	1.3	3.5	3.4	-0.1
	[27.9,32.3]	[31.9,36.0]	[1.8,5.9]	[24.5,28.7]	[28.8,32.3]	[2.0,5.9]	[12.7,15.7]	[15.6,18.0]	[0.9,4.3]	[11.3,13.7]	[12.5,15.2]	[-0.2,2.9]	[2.9,4.2]	[2.9,4.0]	[-0.7,0.6]
Colombia	18.5	19.0	0.5	17.1	17.7	0.6	9.1	10.4	1.3	8.0	7.3	-0.7	1.3	1.3	-0.1
	[15.5,22.0]	[16.0,22.5]	[-4.0,5.0]	[14.3,20.5]	[14.8,21.1]	[-3.7,4.8]	[7.2,11.7]	[8.0,13.5]	[-1.9,4.4]	[6.0,10.7]	[5.8,9.1]	[-3.9,2.5]	[0.8,2.2]	[0.7,2.1]	[-0.9,0.7]
Dominican Republic	32.4	37.9	5.5	29.9	35.8	5.8	22.9	26.6	3.7	7.0	9.2	2.1	2.5	2.2	-0.3
	[27.5,38.1]	[32.9,43.6]	[-1.4,12.5]	[25.4,35.2]	[30.9,41.4]	[-0.8,12.4]	[18.6,28.1]	[22.2,31.8]	[-2.9,10.3]	[5.2,9.5]	[6.7,12.5]	[-1.3,5.6]	[1.4,4.4]	[1.4,3.4]	[-2.0,1.3]
El Salvador	19.1	20.7	1.6	17.5	18.8	1.3	8.6	11.3	2.7	8.9	7.4	-1.5	1.6	2.0	0.3
	[14.9,24.4]	[15.6,27.4]	[-7.1,10.4]	[13.5,22.7]	[14.3,24.5]	[-6.1,8.6]	[6.0,12.3]	[8.3,15.5]	[-2.4,7.9]	[5.7,13.9]	[5.0,11.1]	[-6.2,3.2]	[0.6,4.4]	[0.7,5.2]	[-2.1,2.8]
Guatemala	35.8	41.1	5.3	32.2	37.7	5.5	14.7	20.2	5.5	17.5	17.4	-0.0	3.7	3.5	-0.2
	[32.2,39.9]	[36.6,46.2]	[-0.3,10.9]	[28.6,36.1]	[33.3,42.5]	[0.3,10.7]	[12.2,17.7]	[17.3,23.6]	[1.3,9.7]	[15.0,20.3]	[14.2,21.3]	[-4.1,4.1]	[2.4,5.6]	[2.4,5.1]	[-1.8,1.4]
Guyana	31.4	41.3	10.0	27.3	39.1	11.8	17.2	24.9	7.7	10.0	14.2	4.1	4.1	2.2	-1.9
	[23.3,42.1]	[32.7,52.1]	[-1.5,21.4]	[19.7,37.6]	[30.8,49.5]	[1.3,22.4]	[11.5,25.7]	[18.2,34.1]	[-3.3,18.7]	[6.1,16.6]	[8.8,22.7]	[-2.6,10.8]	[1.5,11.2]	[0.8,6.0]	[-6.4,2.7]
Haiti	76.8	88.8	12.0	63.4	74.4	11.1	30.3	32.8	2.4	33.0	41.7	8.6	13.4	14.3	0.9
	[68.9,85.5]	[79.8,98.6]	[1.0,23.0]	[55.7,71.9]	[66.5,83.3]	[2.0,20.2]	[25.1,36.7]	[26.6,40.3]	[-5.3,10.2]	[28.4,38.3]	[35.5,48.8]	[2.7,14.6]	[10.6,17.0]	[10.9,18.7]	[-4.5,6.2]
Honduras	29.3	29.7	0.3	25.7	27.1	1.4	17.0	16.2	-0.8	8.7	10.9	2.2	3.7	2.6	-1.1
	[25.3,34.0]	[26.2,33.6]	[-4.3,5.0]	[21.9,30.0]	[23.7,30.9]	[-2.9,5.7]	[13.7,21.0]	[13.5,19.4]	[-4.5,3.0]	[7.0,10.7]	[8.4,14.0]	[-0.4,4.7]	[2.6,5.2]	[1.7,3.9]	[-2.8,0.6]
Paraguay	17.4	21.5	4.1	16.9	20.8	3.9	5.3	10.9	5.6	11.6	9.9	-1.7	0.6	0.7	0.1
	[11.9,25.5]	[17.1,27.1]	[-3.8,11.9]	[11.4,25.0]	[16.4,26.5]	[-4.1,12.0]	[3.6,7.8]	[7.9,15.1]	[1.7,9.5]	[6.8,19.5]	[6.5,15.0]	[-8.8,5.4]	[0.2,1.5]	[0.2,2.2]	[-0.6,0.9]
Peru	23.2	27.1	4.0	20.4	24.6	4.2	11.4	13.6	2.2	8.9	10.9	2.0	2.8	2.6	-0.2
	[20.0,26.9]	[23.2,31.8]	[-1.6,9.5]	[17.4,23.9]	[20.7,29.1]	[-1.2,9.6]	[8.9,14.8]	[11.2,16.6]	[-1.5,5.9]	[7.5,10.6]	[8.0,14.9]	[-1.7,5.7]	[1.8,4.6]	[1.6,4.1]	[-2.1,1.6]
Suriname	15.5	24.1	8.6	13.9	21.7	7.8	10.1	16.0	5.9	3.9	5.7	1.8	1.5	2.4	0.9
	[10.0,23.8]	[17.8,32.6]	[-0.3,17.5]	[8.9,21.7]	[15.9,29.5]	[-0.5,16.0]	[5.7,17.7]	[11.4,22.3]	[-1.7,13.5]	[2.1,7.2]	[2.9,11.2]	[-2.3,6.0]	[0.4,6.1]	[1.2,4.8]	[-1.6,3.3]
Low-Income Countries	84.6	98.3	13.8	64.5	77.0	12.4	25.5	35.7	10.2	39.0	41.3	2.3	20.0	21.3	1.3
	[81.9,87.3]	[96.2,100.5]	[11.4,16.1]	[62.6,66.6]	[75.0,78.9]	[10.1,14.7]	[24.2,26.9]	[34.1,37.3]	[8.4,11.9]	[37.3,40.8]	[39.8,42.9]	[0.8,3.8]	[19.0,21.1]	[20.5,22.2]	[0.1,2.6]
Lower-Middle-Income Countries	61.2	67.7	6.5	49.6	56.7	7.1	25.9	34.0	8.1	23.6	22.7	-0.9	11.6	11.0	-0.6
	[59.5,62.9]	[66.2,69.2]	[4.7,8.3]	[48.3,50.9]	[55.5,57.9]	[5.5,8.7]	[25.1,26.8]	[33.2,34.8]	[6.6,9.5]	[22.7,24.6]	[22.0,23.5]	[-1.8,-0.0]	[11.1,12.3]	[10.4,11.6]	[-1.3,0.0]
Upper-Middle-Income Countries	29.2	34.0	4.8	26.3	30.2	3.9	14.0	17.7	3.7	12.3	12.5	0.2	2.8	3.7	0.9
	[27.2,31.2]	[31.5,36.5]	[1.3,8.3]	[24.5,28.3]	[27.8,32.9]	[0.7,7.1]	[12.8,15.4]	[15.7,20.0]	[1.2,6.2]	[11.1,13.6]	[11.2,14.1]	[-1.5,1.9]	[2.3,3.4]	[3.2,4.4]	[0.0,1.8]

Notes: 95% confidence intervals are shown in brackets.

© 2022 Karlsson O et al. *JAMA Network Open*.

eTable 10. Shares of Deaths Among Children Younger Than 5 Years Occurring at Different Ages by Sex

	0–23 months			Neonates			1–23 months			24–59 months		
	Female	Male	Difference	Female	Male	Difference	Female	Male	Difference	Female	Male	Difference
Pooled	80.3	82.6	2.3	39.2	46.2	7.1	41.1	36.3	-4.8	19.7	17.4	-2.3
	[79.5,81.0]	[82.0,83.1]	[1.6,2.9]	[38.4,39.9]	[45.5,47.0]	[6.1,8.0]	[40.3,41.9]	[35.8,36.9]	[-5.6,-4.0]	[19.0,20.5]	[16.9,18.0]	[-2.9,-1.6]
Least Developed Countries	77.8	80.0	2.2	32.8	39.0	6.3	45.0	41.0	-4.1	22.2	20.0	-2.2
	[77.0,78.5]	[79.1,80.9]	[1.0,3.4]	[31.5,34.0]	[37.9,40.2]	[4.9,7.6]	[43.9,46.2]	[40.1,41.8]	[-5.3,-2.8]	[21.5,23.0]	[19.1,20.9]	[-3.4,-1.0]
East Asia & Pacific	88.2	86.8	-1.5	47.5	49.2	1.7	40.7	37.5	-3.2	11.8	13.2	1.5
	[86.1,90.1]	[84.8,88.5]	[-4.2,1.2]	[44.0,51.1]	[46.4,52.1]	[-3.1,6.5]	[38.0,43.5]	[34.6,40.5]	[-7.4,1.0]	[9.9,13.9]	[11.5,15.2]	[-1.2,4.2]
Cambodia	85.3	89.4	4.1	48.4	41.1	-7.3	36.9	48.3	11.4	14.7	10.6	-4.1
	[80.2,89.2]	[85.2,92.5]	[-1.4,9.7]	[42.8,54.0]	[35.4,47.1]	[-15.7,1.1]	[31.7,42.5]	[42.1,54.6]	[3.2,19.6]	[10.8,19.8]	[7.5,14.8]	[-9.7,1.4]
Indonesia	89.0	86.5	-2.4	49.1	52.4	3.4	39.9	34.1	-5.8	11.0	13.5	2.4
	[84.9,92.0]	[82.1,90.0]	[-7.3,2.4]	[43.2,55.0]	[47.9,57.0]	[-3.4,10.1]	[34.9,45.1]	[30.1,38.3]	[-11.7,0.1]	[8.0,15.1]	[10.0,17.9]	[-2.4,7.3]
Lao	90.1	92.3	2.2	42.7	40.0	-2.6	47.4	52.3	4.9	9.9	7.7	-2.2
	[86.8,92.6]	[89.8,94.2]	[-1.1,5.6]	[37.9,47.6]	[35.3,44.9]	[-9.5,4.2]	[41.7,53.2]	[47.0,57.5]	[-3.5,13.2]	[7.4,13.2]	[5.8,10.2]	[-5.6,1.1]
Mongolia	87.6	80.6	-7.0	40.6	46.2	5.5	46.9	34.4	-12.5	12.4	19.4	7.0
	[76.2,93.9]	[67.7,89.1]	[-20.3,6.3]	[28.7,53.8]	[33.2,59.6]	[-14.6,25.6]	[34.2,60.1]	[23.8,46.9]	[-33.3,8.3]	[6.1,23.8]	[10.9,32.3]	[-6.3,20.3]
Myanmar	90.1	86.2	-3.9	44.7	44.2	-0.5	45.4	42.1	-3.4	9.9	13.8	3.9
	[85.9,93.2]	[81.6,89.8]	[-9.7,1.8]	[37.2,52.5]	[36.7,51.9]	[-10.3,9.2]	[38.3,52.8]	[35.9,48.5]	[-12.8,6.0]	[6.8,14.1]	[10.2,18.4]	[-1.8,9.7]
Papua New Guinea	83.4	78.4	-5.0	42.4	48.4	6.0	41.0	30.0	-11.0	16.6	21.6	5.0
	[78.1,87.7]	[72.6,83.3]	[-11.6,1.7]	[35.8,49.3]	[41.0,55.9]	[-5.0,17.0]	[34.7,47.6]	[22.6,38.7]	[-22.8,0.9]	[12.3,21.9]	[16.7,27.4]	[-1.7,11.6]
Philippines	85.9	88.2	2.3	48.4	50.2	1.8	37.5	38.0	0.5	14.1	11.8	-2.3
	[79.8,90.4]	[83.4,91.8]	[-5.1,9.8]	[40.4,56.4]	[43.1,57.2]	[-8.1,11.8]	[29.8,45.9]	[31.7,44.8]	[-9.7,10.7]	[9.6,20.2]	[8.2,16.6]	[-9.8,5.1]
Timor-Leste	80.7	79.8	-0.8	35.5	46.0	10.5	45.1	33.8	-11.3	19.3	20.2	0.8
	[74.9,85.4]	[72.9,85.3]	[-9.1,7.4]	[27.8,44.1]	[38.8,53.4]	[-0.6,21.6]	[37.9,52.6]	[26.4,42.2]	[-22.6,-0.0]	[14.6,25.1]	[14.7,27.1]	[-7.4,9.1]
Tonga	NA	88.1	NA	52.0	40.8	-11.2	48.0	47.3	-0.7	NA	11.9	NA
		[63.2,97.0]		[18.2,84.1]	[19.2,66.6]	[-68.9,46.5]	[15.9,81.8]	[20.1,76.3]	[-60.0,58.7]		[3.0,36.8]	
South Asia	87.7	90.8	3.1	51.8	62.6	10.8	35.8	28.1	-7.7	12.3	9.2	-3.1
	[86.9,88.4]	[90.3,91.3]	[2.1,4.1]	[50.5,53.2]	[61.4,63.9]	[9.1,12.5]	[34.5,37.2]	[27.0,29.3]	[-9.3,-6.0]	[11.6,13.1]	[8.7,9.7]	[-4.1,-2.1]
Afghanistan	89.8	89.2	-0.6	36.3	43.2	6.9	53.5	46.0	-7.5	10.2	10.8	0.6
	[86.5,92.3]	[85.8,91.9]	[-5.8,4.7]	[32.1,40.8]	[40.3,46.2]	[2.6,11.2]	[48.7,58.1]	[42.5,49.6]	[-13.8,-1.2]	[7.7,13.5]	[8.1,14.2]	[-4.7,5.8]
Bangladesh	88.2	89.6	1.4	60.4	66.7	6.4	27.8	22.9	-4.9	11.8	10.4	-1.4
	[85.2,90.6]	[87.3,91.5]	[-2.2,5.1]	[56.4,64.3]	[63.3,70.0]	[1.7,11.0]	[24.0,31.9]	[19.9,26.1]	[-9.1,-0.7]	[9.4,14.8]	[8.5,12.7]	[-5.1,2.2]
India	87.3	90.2	2.9	52.8	63.2	10.4	34.5	27.0	-7.5	12.7	9.8	-2.9
	[86.6,88.0]	[89.4,90.8]	[1.7,4.1]	[51.8,53.9]	[62.0,64.5]	[8.8,11.9]	[33.4,35.5]	[25.8,28.1]	[-8.8,-6.2]	[12.0,13.4]	[9.2,10.6]	[-4.1,-1.7]
Maldives	95.2	86.0	-9.2	57.0	59.3	2.3	38.2	26.7	-11.5	4.8	14.0	9.2
	[86.3,98.4]	[72.2,93.5]	[-20.9,2.5]	[39.1,73.2]	[39.7,76.3]	[-21.8,26.3]	[23.0,56.1]	[13.9,45.1]	[-30.0,7.0]	[1.6,13.7]	[6.5,27.8]	[-2.5,20.9]
Nepal	84.8	92.6	7.8	37.6	66.7	29.2	47.2	25.8	-21.4	15.2	7.4	-7.8
	[73.9,91.6]	[87.9,95.6]	[-1.3,16.9]	[28.4,47.8]	[59.9,73.0]	[16.7,41.6]	[38.6,56.0]	[20.2,32.5]	[-32.0,-10.7]	[8.4,26.1]	[4.4,12.1]	[-16.9,1.3]
Pakistan	88.3	93.0	4.7	49.6	63.0	13.4	38.7	30.0	-8.7	11.7	7.0	-4.7
	[85.4,90.7]	[91.0,94.6]	[1.5,7.8]	[44.7,54.5]	[57.8,67.9]	[6.9,19.8]	[33.6,44.1]	[25.6,34.9]	[-15.0,-2.3]	[9.3,14.6]	[5.4,9.0]	[-7.8,-1.5]
Europe & Central Asia	92.6	90.5	-2.0	46.7	51.0	4.3	45.9	39.5	-6.3	7.4	9.5	2.0
	[88.0,95.5]	[85.2,94.1]	[-7.2,3.1]	[37.0,56.7]	[44.7,57.3]	[-5.7,14.3]	[36.9,55.1]	[33.1,46.3]	[-17.0,4.3]	[4.5,12.0]	[5.9,14.8]	[-3.1,7.2]
Albania	93.0	NA	NA	54.2	60.3	6.1	38.8	39.7	0.9	7.0	NA	NA
	[61.8,99.1]			[17.1,87.2]	[31.2,83.6]	[-44.5,56.6]	[9.0,80.3]	[16.4,68.8]	[-45.5,47.3]	[0.9,38.2]		
Armenia	98.6	84.3	-14.3	53.0	48.4	-4.6	45.6	35.9	-9.7	1.4	15.7	14.3
	[94.4,99.7]	[60.5,95.0]	[-33.6,5.0]	[31.6,73.3]	[24.6,73.0]	[-42.4,33.3]	[25.4,67.4]	[14.5,64.9]	[-50.6,31.1]	[0.3,5.6]	[5.0,39.5]	[-5.0,33.6]
Kyrgyz Republic	96.4	89.5	-6.9	64.0	63.2	-0.8	32.4	26.3	-6.1	3.6	10.5	6.9
	[90.9,98.7]	[77.7,95.5]	[-16.1,2.2]	[48.6,77.1]	[47.4,76.6]	[-19.3,17.6]	[19.4,48.9]	[15.7,40.6]	[-22.2,10.0]	[1.3,9.1]	[4.5,22.3]	[-2.2,16.1]
Moldova	98.1	91.0	-7.1	74.7	75.8	1.1	23.4	15.2	-8.2	1.9	9.0	7.1

© 2022 Karlsson O et al. *JAMA Network Open*.

	[91.4,99.6]	[49.6,99.1]	[-23.0,8.8]	[51.8,89.0]	[49.0,91.1]	[-29.7,31.9]	[9.4,47.4]	[4.9,38.6]	[-34.9,18.5]	[0.4,8.6]	[0.9,50.4]	[-8.8,23.0]
Tajikistan	90.7	90.8	0.1	40.7	47.2	6.5	50.0	43.6	-6.3	9.3	9.2	-0.1
	[81.7,95.5]	[84.4,94.7]	[-8.7,8.9]	[32.6,49.5]	[38.2,56.3]	[-5.5,18.4]	[40.8,59.1]	[34.9,52.7]	[-19.8,7.1]	[4.5,18.3]	[5.3,15.6]	[-8.9,8.7]
Turkey	93.0	90.9	-2.1	44.6	48.3	3.7	48.4	42.6	-5.8	7.0	9.1	2.1
	[83.6,97.2]	[79.9,96.2]	[-10.4,6.3]	[32.3,57.6]	[34.0,63.0]	[-17.7,25.1]	[34.2,62.8]	[31.2,54.8]	[-27.2,15.7]	[2.8,16.4]	[3.8,20.1]	[-6.3,10.4]
Turkmenistan	87.3	88.0	0.8	52.4	54.7	2.4	34.9	33.3	-1.6	12.7	12.0	-0.8
	[77.4,93.2]	[81.9,92.3]	[-8.7,10.2]	[39.3,65.1]	[46.1,63.1]	[-13.4,18.1]	[22.8,49.2]	[25.1,42.6]	[-17.2,14.0]	[6.8,22.6]	[7.7,18.1]	[-10.2,8.7]
Middle East & North Africa	91.6	91.1	-0.6	47.8	54.5	6.7	43.8	36.6	-7.2	8.4	8.9	0.6
	[90.0,93.0]	[89.1,92.7]	[-3.3,2.2]	[45.4,50.3]	[51.5,57.5]	[2.1,11.3]	[41.5,46.2]	[33.8,39.4]	[-11.7,-2.8]	[7.0,10.0]	[7.3,10.9]	[-2.2,3.3]
Egypt	92.8	90.4	-2.3	49.9	57.5	7.6	42.9	33.0	-10.0	7.2	9.6	2.3
	[89.8,94.9]	[85.5,93.8]	[-7.3,2.6]	[44.1,55.6]	[52.2,62.5]	[0.4,14.8]	[37.8,48.3]	[28.9,37.3]	[-16.7,-3.2]	[5.1,10.2]	[6.2,14.5]	[-2.6,7.3]
Iraq	90.2	90.6	0.4	52.9	52.6	-0.3	37.4	38.0	0.7	9.8	9.4	-0.4
	[85.0,93.8]	[87.5,93.0]	[-4.3,5.0]	[46.1,59.5]	[47.1,58.0]	[-9.2,8.6]	[31.9,43.2]	[32.6,43.8]	[-6.8,8.1]	[6.2,15.0]	[7.0,12.5]	[-5.0,4.3]
Jordan	93.2	91.5	-1.7	48.4	60.0	11.6	44.8	31.5	-13.3	6.8	8.5	1.7
	[84.1,97.2]	[86.0,94.9]	[-8.5,5.1]	[36.0,61.0]	[49.9,69.3]	[-2.5,25.7]	[32.5,57.7]	[23.7,40.5]	[-27.3,0.7]	[2.8,15.9]	[5.1,14.0]	[-5.1,8.5]
State of Palestine	91.0	90.3	-0.7	48.1	51.8	3.7	42.9	38.5	-4.4	9.0	9.7	0.7
	[83.7,95.2]	[84.7,93.9]	[-7.2,5.7]	[40.8,55.5]	[43.1,60.3]	[-7.8,15.1]	[35.6,50.6]	[30.7,47.0]	[-15.6,6.8]	[4.8,16.3]	[6.1,15.3]	[-5.7,7.2]
Tunisia	92.5	91.5	-1.0	64.5	55.6	-8.9	28.0	35.9	7.9	7.5	8.5	1.0
	[77.4,97.8]	[82.3,96.2]	[-12.3,10.3]	[46.7,79.1]	[44.1,66.5]	[-30.0,12.2]	[15.5,45.2]	[25.8,47.4]	[-12.9,28.7]	[2.2,22.6]	[3.8,17.7]	[-10.3,12.3]
Yemen	90.4	92.2	1.8	40.7	50.9	10.2	49.7	41.3	-8.4	9.6	7.8	-1.8
	[87.9,92.5]	[89.6,94.2]	[-0.5,4.1]	[36.4,45.2]	[46.4,55.5]	[3.7,16.7]	[45.4,54.0]	[36.6,46.1]	[-15.0,-1.8]	[7.5,12.1]	[5.8,10.4]	[-4.1,0.5]
Eastern & Southern Africa	80.4	83.3	2.9	35.3	42.7	7.4	45.1	40.6	-4.5	19.6	16.7	-2.9
	[78.9,81.8]	[81.9,84.5]	[1.3,4.5]	[33.7,36.9]	[40.7,44.7]	[5.1,9.7]	[43.4,46.7]	[39.1,42.1]	[-6.4,-2.6]	[18.2,21.1]	[15.5,18.1]	[-4.5,-1.3]
Angola	72.9	76.8	3.9	25.9	34.6	8.7	47.0	42.2	-4.8	27.1	23.2	-3.9
	[69.2,76.3]	[73.2,80.0]	[0.1,7.7]	[22.0,30.1]	[30.1,39.4]	[2.4,15.1]	[42.7,51.3]	[38.1,46.3]	[-10.3,0.6]	[23.7,30.8]	[20.0,26.8]	[-7.7,-0.1]
Burundi	74.5	77.1	2.6	29.8	31.1	1.3	44.7	46.1	1.3	25.5	22.9	-2.6
	[70.7,78.0]	[74.0,80.0]	[-1.9,7.1]	[26.3,33.5]	[27.5,34.8]	[-3.5,6.0]	[40.8,48.8]	[43.4,48.7]	[-3.8,6.4]	[22.0,29.3]	[20.0,26.0]	[-7.1,1.9]
Comoros	78.3	89.5	11.1	44.3	58.0	13.8	34.1	31.4	-2.6	21.7	10.5	-11.1
	[69.3,85.3]	[83.5,93.5]	[1.9,20.3]	[33.5,55.6]	[50.1,65.6]	[0.5,27.0]	[24.6,45.0]	[24.2,39.7]	[-13.9,8.6]	[14.7,30.7]	[6.5,16.5]	[-20.3,-1.9]
Ethiopia	80.5	86.4	5.9	39.1	51.7	12.6	41.4	34.7	-6.7	19.5	13.6	-5.9
	[76.2,84.1]	[82.7,89.4]	[0.8,11.1]	[33.0,45.4]	[46.6,56.7]	[5.5,19.8]	[35.9,47.1]	[30.8,38.8]	[-12.9,-0.5]	[15.9,23.8]	[10.6,17.3]	[-11.1,-0.8]
Kenya	83.7	83.6	-0.1	39.5	42.2	2.7	44.2	41.5	-2.7	16.3	16.4	0.1
	[80.3,86.6]	[80.2,86.6]	[-4.0,3.9]	[35.1,44.2]	[37.9,46.6]	[-1.7,7.0]	[39.8,48.7]	[37.5,45.5]	[-7.8,2.4]	[13.4,19.7]	[13.4,19.8]	[-3.9,4.0]
Lesotho	86.0	91.2	5.2	50.3	46.3	-4.0	35.6	44.9	9.3	14.0	8.8	-5.2
	[76.8,91.9]	[84.9,95.0]	[-3.0,13.4]	[39.4,61.2]	[39.4,53.3]	[-15.2,7.1]	[26.5,46.0]	[36.8,53.3]	[-2.2,20.7]	[8.1,23.2]	[5.0,15.1]	[-13.4,3.0]
Madagascar	74.7	74.9	0.2	35.9	36.1	0.1	38.8	38.8	0.0	25.3	25.1	-0.2
	[70.6,78.4]	[71.4,78.0]	[-4.7,5.1]	[31.8,40.3]	[32.1,40.2]	[-5.1,5.4]	[34.7,43.0]	[35.1,42.6]	[-4.0,4.1]	[21.6,29.4]	[22.0,28.6]	[-5.1,4.7]
Malawi	75.1	77.6	2.5	31.7	38.3	6.6	43.4	39.3	-4.1	24.9	22.4	-2.5
	[71.7,78.2]	[74.4,80.6]	[-1.0,6.1]	[28.3,35.3]	[35.0,41.8]	[3.1,10.1]	[39.8,47.0]	[36.4,42.3]	[-8.5,0.4]	[21.8,28.3]	[19.4,25.6]	[-6.1,1.0]
Mozambique	79.2	78.7	-0.5	26.5	32.4	5.9	52.8	46.4	-6.4	20.8	21.3	0.5
	[75.6,82.5]	[75.3,81.8]	[-5.1,4.1]	[23.1,30.2]	[28.8,36.2]	[0.4,11.4]	[48.8,56.7]	[42.4,50.4]	[-12.1,-0.7]	[17.5,24.4]	[18.2,24.7]	[-4.1,5.1]
Namibia	82.4	82.1	-0.3	26.4	36.5	10.1	56.0	45.6	-10.4	17.6	17.9	0.3
	[75.9,87.4]	[77.0,86.2]	[-8.7,8.1]	[20.4,33.3]	[30.1,43.3]	[2.0,18.3]	[48.5,63.3]	[39.2,52.2]	[-19.9,-0.9]	[12.6,24.1]	[13.8,23.0]	[-8.1,8.7]
Rwanda	77.6	76.8	-0.8	31.0	35.1	4.1	46.6	41.7	-4.9	22.4	23.2	0.8
	[73.8,81.0]	[73.6,79.7]	[-5.5,3.9]	[27.6,34.7]	[31.2,39.1]	[-0.8,8.9]	[42.4,50.8]	[37.9,45.7]	[-10.8,1.1]	[19.0,26.2]	[20.3,26.4]	[-3.9,5.5]
South Africa	93.1	91.9	-1.2	45.2	51.3	6.1	47.9	40.5	-7.3	6.9	8.1	1.2
	[87.7,96.2]	[86.7,95.1]	[-7.1,4.6]	[31.4,59.9]	[41.6,61.0]	[-9.5,21.7]	[34.0,62.0]	[31.5,50.3]	[-22.5,7.8]	[3.8,12.3]	[4.9,13.3]	[-4.6,7.1]
South Sudan	81.7	81.8	0.2	35.8	38.2	2.5	45.9	43.6	-2.3	18.3	18.2	-0.2
	[78.2,84.7]	[78.7,84.6]	[-3.4,3.8]	[30.2,41.8]	[34.2,42.4]	[-1.6,6.6]	[40.5,51.4]	[40.1,47.2]	[-6.8,2.2]	[15.3,21.8]	[15.4,21.3]	[-3.8,3.4]
Sudan	86.9	86.5	-0.4	40.9	46.6	5.8	46.0	39.9	-6.2	13.1	13.5	0.4
	[83.5,89.7]	[83.6,89.0]	[-4.8,4.0]	[36.5,45.4]	[43.1,50.3]	[0.9,10.7]	[41.5,50.6]	[36.5,43.4]	[-12.1,-0.2]	[10.3,16.5]	[11.0,16.4]	[-4.0,4.8]
Swaziland	89.9	84.5	-5.4	25.3	21.6	-3.7	64.6	62.9	-1.7	10.1	15.5	5.4

© 2022 Karlsson O et al. *JAMA Network Open*.

	[83.7,93.9]	[77.3,89.7]	[-13.5,2.7]	[18.7,33.4]	[15.1,29.9]	[-12.7,5.3]	[55.8,72.4]	[55.6,69.6]	[-14.4,11.0]	[6.1,16.3]	[10.3,22.7]	[-2.7,13.5]
Tanzania	78.6	86.9	8.3	33.2	40.9	7.6	45.3	46.0	0.7	21.4	13.1	-8.3
	[74.1,82.5]	[83.8,89.5]	[3.2,13.5]	[28.9,37.9]	[37.0,44.9]	[2.0,13.3]	[41.0,49.8]	[42.1,50.0]	[-4.7,6.1]	[17.5,25.9]	[10.5,16.2]	[-13.5,-3.2]
Uganda	80.0	81.0	1.0	35.8	38.8	3.0	44.3	42.3	-2.0	20.0	19.0	-1.0
	[76.7,83.0]	[78.1,83.7]	[-3.5,5.5]	[33.0,38.6]	[35.9,41.7]	[-1.0,7.0]	[40.6,48.0]	[39.1,45.5]	[-7.2,3.2]	[17.0,23.3]	[16.3,21.9]	[-5.5,3.5]
Zambia	80.7	79.3	-1.4	34.0	43.0	8.9	46.7	36.4	-10.3	19.3	20.7	1.4
	[76.4,84.3]	[75.2,82.9]	[-7.5,4.8]	[27.9,40.8]	[37.5,48.6]	[-0.8,18.7]	[41.7,51.7]	[32.7,40.2]	[-17.0,-3.6]	[15.7,23.6]	[17.1,24.8]	[-4.8,7.5]
Zimbabwe	83.3	84.9	1.6	40.2	45.9	5.7	43.0	39.0	-4.1	16.7	15.1	-1.6
	[77.7,87.7]	[81.0,88.0]	[-4.2,7.5]	[35.0,45.7]	[40.3,51.6]	[-0.6,12.0]	[37.4,48.8]	[33.7,44.5]	[-10.7,2.6]	[12.3,22.3]	[12.0,19.0]	[-7.5,4.2]
West & Central Africa	69.8	71.4	1.6	26.8	30.7	3.9	43.0	40.7	-2.3	30.2	28.6	-1.6
	[68.8,70.8]	[70.3,72.5]	[0.5,2.7]	[25.6,28.1]	[29.8,31.7]	[2.7,5.1]	[42.0,44.0]	[39.7,41.7]	[-3.4,-1.2]	[29.2,31.2]	[27.5,29.7]	[-2.7,-0.5]
Benin	70.9	73.4	2.6	27.7	35.4	7.7	43.2	38.1	-5.1	29.1	26.6	-2.6
	[67.6,73.9]	[70.7,76.0]	[-1.0,6.1]	[25.1,30.3]	[32.1,38.8]	[3.9,11.5]	[39.6,46.8]	[34.9,41.4]	[-9.9,-0.4]	[26.1,32.4]	[24.0,29.3]	[-6.1,1.0]
Burkina Faso	70.8	72.1	1.3	21.8	24.5	2.7	49.0	47.6	-1.4	29.2	27.9	-1.3
	[69.0,72.6]	[70.0,74.1]	[-1.4,4.0]	[20.0,23.7]	[22.4,26.7]	[-0.0,5.4]	[46.7,51.3]	[44.9,50.3]	[-4.7,1.9]	[27.4,31.0]	[25.9,30.0]	[-4.0,1.4]
Cameroon	71.8	73.4	1.6	31.9	34.2	2.4	39.9	39.2	-0.7	28.2	26.6	-1.6
	[68.0,75.2]	[69.9,76.6]	[-3.4,6.6]	[27.8,36.2]	[30.6,38.1]	[-2.8,7.5]	[35.3,44.7]	[35.3,43.1]	[-6.6,5.2]	[24.8,32.0]	[23.4,30.1]	[-6.6,3.4]
Chad	71.2	74.2	3.0	21.0	27.4	6.4	50.2	46.8	-3.4	28.8	25.8	-3.0
	[67.6,74.6]	[70.6,77.6]	[-0.2,6.2]	[18.9,23.4]	[25.6,29.4]	[3.5,9.3]	[46.7,53.7]	[43.4,50.2]	[-6.1,-0.7]	[25.4,32.4]	[22.4,29.4]	[-6.2,0.2]
Congo	77.3	68.8	-8.4	27.4	31.9	4.4	49.9	37.0	-12.9	22.7	31.2	8.4
	[71.4,82.2]	[63.8,73.5]	[-16.1,-0.8]	[21.8,33.8]	[26.8,37.5]	[-3.3,12.2]	[42.8,56.9]	[32.3,41.8]	[-22.2,-3.6]	[17.8,28.6]	[26.5,36.2]	[0.8,16.1]
Congo (DR)	73.6	73.0	-0.6	26.0	28.0	2.1	47.7	45.0	-2.7	26.4	27.0	0.6
	[71.3,75.8]	[70.0,75.9]	[-4.1,2.9]	[22.8,29.4]	[24.9,31.5]	[-2.4,6.6]	[44.0,51.4]	[42.1,47.9]	[-6.6,1.2]	[24.2,28.7]	[24.1,30.0]	[-2.9,4.1]
Cote D'Ivoire	78.9	78.3	-0.6	33.5	36.3	2.8	45.4	42.0	-3.4	21.1	21.7	0.6
	[75.3,82.1]	[73.7,82.3]	[-5.2,4.0]	[29.3,37.9]	[32.9,39.8]	[-2.8,8.5]	[40.7,50.2]	[37.7,46.4]	[-8.3,1.5]	[17.9,24.7]	[17.7,26.3]	[-4.0,5.2]
Gabon	81.1	77.9	-3.2	42.0	38.3	-3.8	39.0	39.6	0.6	18.9	22.1	3.2
	[73.5,86.9]	[71.2,83.4]	[-13.9,7.5]	[32.3,52.5]	[31.0,46.1]	[-14.6,7.1]	[30.9,47.8]	[31.5,48.3]	[-8.5,9.6]	[13.1,26.5]	[16.6,28.8]	[-7.5,13.9]
Gambia	79.7	82.6	2.9	45.0	52.9	7.9	34.8	29.8	-5.0	20.3	17.4	-2.9
	[74.5,84.1]	[79.0,85.7]	[-3.1,8.9]	[39.5,50.6]	[47.7,58.1]	[-0.2,16.0]	[29.7,40.2]	[25.4,34.6]	[-11.7,1.7]	[15.9,25.5]	[14.3,21.0]	[-8.9,3.1]
Ghana	80.2	77.2	-3.0	43.1	44.8	1.6	37.1	32.4	-4.6	19.8	22.8	3.0
	[75.3,84.4]	[70.3,82.9]	[-10.5,4.4]	[35.5,51.2]	[39.5,50.2]	[-7.4,10.6]	[30.8,43.8]	[26.6,38.9]	[-12.2,2.9]	[15.6,24.7]	[17.1,29.7]	[-4.4,10.5]
Guinea	73.0	74.7	1.7	28.3	32.8	4.5	44.7	41.9	-2.8	27.0	25.3	-1.7
	[69.3,76.5]	[71.6,77.6]	[-3.3,6.7]	[25.0,31.9]	[28.7,37.2]	[-0.3,9.2]	[40.4,49.2]	[37.9,46.1]	[-8.0,2.4]	[23.5,30.7]	[22.4,28.4]	[-6.7,3.3]
Liberia	78.0	80.0	2.0	27.0	32.5	5.5	51.0	47.5	-3.5	22.0	20.0	-2.0
	[73.4,82.0]	[77.0,82.7]	[-3.1,7.1]	[22.6,31.9]	[28.4,36.9]	[-0.5,11.5]	[44.4,57.5]	[43.2,51.9]	[-10.7,3.8]	[18.0,26.6]	[17.3,23.0]	[-7.1,3.1]
Mali	66.9	68.3	1.5	29.4	31.4	2.1	37.5	36.9	-0.6	33.1	31.7	-1.5
	[63.0,70.5]	[65.4,71.1]	[-3.0,6.0]	[25.4,33.6]	[27.7,35.4]	[-1.8,5.9]	[34.2,41.0]	[33.5,40.4]	[-4.9,3.7]	[29.5,37.0]	[28.9,34.6]	[-6.0,3.0]
Mauritania	82.7	85.8	3.1	48.6	55.5	6.9	34.1	30.3	-3.8	17.3	14.2	-3.1
	[78.2,86.4]	[81.4,89.3]	[-3.0,9.3]	[43.2,54.0]	[50.1,60.7]	[-0.3,14.1]	[28.7,39.9]	[26.0,35.1]	[-10.9,3.4]	[13.6,21.8]	[10.7,18.6]	[-9.3,3.0]
Niger	61.8	65.3	3.5	16.9	23.8	6.9	44.9	41.5	-3.4	38.2	34.7	-3.5
	[59.4,64.2]	[62.5,68.1]	[0.1,6.9]	[15.1,18.9]	[21.3,26.5]	[4.5,9.4]	[42.5,47.4]	[38.8,44.3]	[-6.3,-0.6]	[35.8,40.6]	[31.9,37.5]	[-6.9,-0.1]
Nigeria	66.9	69.4	2.6	26.5	30.5	4.0	40.4	38.9	-1.5	33.1	30.6	-2.6
	[65.1,68.7]	[67.4,71.4]	[0.1,5.0]	[24.2,28.8]	[28.4,32.7]	[1.7,6.4]	[38.5,42.4]	[36.9,40.9]	[-3.7,0.7]	[31.3,34.9]	[28.6,32.6]	[-5.0,-0.1]
Sao Tome & Principe	89.1	79.0	-10.1	35.3	46.4	11.2	53.9	32.6	-21.2	10.9	21.0	10.1
	[75.7,95.6]	[69.5,86.2]	[-21.6,1.5]	[22.7,50.2]	[35.9,57.3]	[-6.2,28.5]	[39.7,67.4]	[23.4,43.4]	[-38.1,-4.4]	[4.4,24.3]	[13.8,30.5]	[-1.5,21.6]
Senegal	82.2	85.0	2.8	47.4	51.1	3.7	34.8	33.9	-0.9	17.8	15.0	-2.8
	[76.1,87.0]	[79.6,89.2]	[-3.9,9.5]	[40.2,54.7]	[44.7,57.5]	[-6.2,13.7]	[29.1,41.1]	[28.2,40.2]	[-10.4,8.5]	[13.0,23.9]	[10.8,20.4]	[-9.5,3.9]
Sierra Leone	77.9	77.1	-0.8	21.2	26.0	4.8	56.7	51.1	-5.7	22.1	22.9	0.8
	[74.7,80.8]	[73.0,80.7]	[-6.3,4.6]	[18.2,24.5]	[23.1,29.1]	[0.5,9.2]	[53.3,60.0]	[47.5,54.7]	[-10.0,-1.3]	[19.2,25.3]	[19.3,27.0]	[-4.6,6.3]
Togo	70.5	66.7	-3.8	38.1	37.0	-1.1	32.5	29.7	-2.7	29.5	33.3	3.8
	[63.0,77.1]	[60.9,72.1]	[-11.8,4.2]	[30.5,46.3]	[31.4,43.0]	[-10.7,8.6]	[25.8,39.9]	[23.8,36.4]	[-13.0,7.6]	[22.9,37.0]	[27.9,39.1]	[-4.2,11.8]
Latin America & Caribbean	88.4	90.0	1.6	47.0	49.4	2.4	41.4	40.6	-0.8	11.6	10.0	-1.6

© 2022 Karlsson O et al. *JAMA Network Open*.

	[86.2,90.3]	[88.7,91.1]	[-0.4,3.5]	[43.9,50.2]	[46.5,52.3]	[-2.0,6.7]	[38.8,44.0]	[37.6,43.6]	[-5.0,3.4]	[9.7,13.8]	[8.9,11.3]	[-3.5,0.4]
Colombia	92.7	93.3	0.6	49.4	54.9	5.5	43.3	38.5	-4.9	7.3	6.7	-0.6
	[88.5,95.5]	[88.8,96.1]	[-3.4,4.7]	[40.3,58.5]	[46.7,62.8]	[-7.9,18.8]	[34.3,52.8]	[30.8,46.7]	[-17.9,8.2]	[4.5,11.5]	[3.9,11.2]	[-4.7,3.4]
Dominican Republic	92.4	94.3	1.9	70.7	70.1	-0.5	21.7	24.2	2.5	7.6	5.7	-1.9
	[87.2,95.6]	[91.2,96.4]	[-2.8,6.7]	[63.1,77.2]	[62.5,76.8]	[-10.6,9.5]	[15.8,29.0]	[17.8,31.9]	[-7.3,12.2]	[4.4,12.8]	[3.6,8.8]	[-6.7,2.8]
El Salvador	91.6	90.5	-1.0	45.0	54.7	9.7	46.6	35.8	-10.8	8.4	9.5	1.0
	[78.9,96.9]	[79.3,96.0]	[-10.8,8.7]	[31.3,59.5]	[44.1,64.9]	[-7.3,26.8]	[32.9,60.8]	[26.2,46.8]	[-28.3,6.8]	[3.1,21.1]	[4.0,20.7]	[-8.7,10.8]
Guatemala	89.8	91.5	1.8	41.0	49.1	8.1	48.7	42.4	-6.3	10.2	8.5	-1.8
	[84.7,93.3]	[88.0,94.1]	[-2.2,5.7]	[35.0,47.3]	[43.4,54.9]	[-0.7,16.9]	[43.2,54.3]	[36.3,48.7]	[-14.8,2.1]	[6.7,15.3]	[5.9,12.0]	[-5.7,2.2]
Guyana	86.9	94.6	7.7	54.9	60.3	5.4	32.0	34.3	2.3	13.1	5.4	-7.7
	[69.4,95.1]	[86.2,98.0]	[-4.8,20.2]	[41.5,67.6]	[45.4,73.5]	[-13.6,24.5]	[19.9,47.2]	[21.7,49.6]	[-17.1,21.6]	[4.9,30.6]	[2.0,13.8]	[-20.2,4.8]
Haiti	82.5	83.9	1.4	39.5	36.9	-2.6	43.0	46.9	3.9	17.5	16.1	-1.4
	[78.1,86.2]	[79.5,87.5]	[-4.2,6.9]	[34.3,45.0]	[30.8,43.5]	[-9.9,4.8]	[38.4,47.7]	[40.9,53.1]	[-2.4,10.3]	[13.8,21.9]	[12.5,20.5]	[-6.9,4.2]
Honduras	87.5	91.2	3.7	57.9	54.6	-3.3	29.6	36.6	7.0	12.5	8.8	-3.7
	[82.9,91.0]	[87.2,94.1]	[-1.7,9.2]	[50.6,64.9]	[47.1,62.0]	[-11.6,5.0]	[24.0,35.8]	[29.6,44.2]	[-0.6,14.7]	[9.0,17.1]	[5.9,12.8]	[-9.2,1.7]
Paraguay	96.8	96.8	-0.0	30.5	50.8	20.3	66.3	46.0	-20.3	3.2	3.2	0.0
	[91.9,98.8]	[88.9,99.1]	[-4.4,4.3]	[19.6,44.2]	[37.5,64.0]	[2.8,37.8]	[52.2,78.0]	[32.7,59.9]	[-38.6,-2.0]	[1.2,8.1]	[0.9,11.1]	[-4.3,4.4]
Peru	87.8	90.5	2.7	49.3	50.2	0.9	38.5	40.3	1.8	12.2	9.5	-2.7
	[81.3,92.3]	[85.2,94.0]	[-4.8,10.2]	[41.9,56.8]	[42.6,57.8]	[-9.1,10.8]	[31.5,45.9]	[31.8,49.4]	[-9.7,13.3]	[7.7,18.7]	[6.0,14.8]	[-10.2,4.8]
Suriname	90.1	90.1	-0.0	65.0	66.3	1.3	25.1	23.8	-1.3	9.9	9.9	0.0
	[68.6,97.4]	[82.2,94.7]	[-12.5,12.4]	[43.7,81.6]	[50.8,78.9]	[-22.0,24.6]	[12.7,43.6]	[13.0,39.4]	[-21.2,18.5]	[2.6,31.4]	[5.3,17.8]	[-12.4,12.5]
Low-Income Countries	76.3	78.3	2.0	30.2	36.3	6.1	46.1	42.0	-4.1	23.7	21.7	-2.0
	[75.4,77.2]	[77.5,79.1]	[0.7,3.3]	[28.8,31.7]	[34.9,37.8]	[4.5,7.7]	[44.8,47.4]	[40.8,43.1]	[-5.5,-2.8]	[22.8,24.6]	[20.9,22.5]	[-3.3,-0.7]
Lower-Middle-Income Countries	81.0	83.8	2.8	42.4	50.2	7.8	38.6	33.6	-5.1	19.0	16.2	-2.8
	[80.3,81.7]	[83.1,84.5]	[1.9,3.6]	[41.3,43.4]	[49.3,51.2]	[6.3,9.3]	[37.5,39.7]	[32.8,34.3]	[-6.4,-3.7]	[18.3,19.7]	[15.5,16.9]	[-3.6,-1.9]
Upper-Middle-Income Countries	90.3	89.0	-1.3	48.1	52.1	4.0	42.2	36.9	-5.3	9.7	11.0	1.3
	[88.3,92.0]	[86.9,90.8]	[-3.8,1.2]	[45.1,51.1]	[48.1,56.1]	[-0.9,8.9]	[39.2,45.2]	[33.2,40.7]	[-9.7,-0.9]	[8.0,11.7]	[9.2,13.1]	[-1.2,3.8]

Notes: 95% confidence intervals are shown in brackets.

© 2022 Karlsson O et al. *JAMA Network Open*.

eFigure 1. Mortality Rate Among Children Younger Than 5 Years and Share of These Deaths Occurring at Different Ages: Pooled, Least Developed Countries, Regions, and World Bank Income Groups: Period Restricted to 5 Years Before Survey Instead of 10 Years

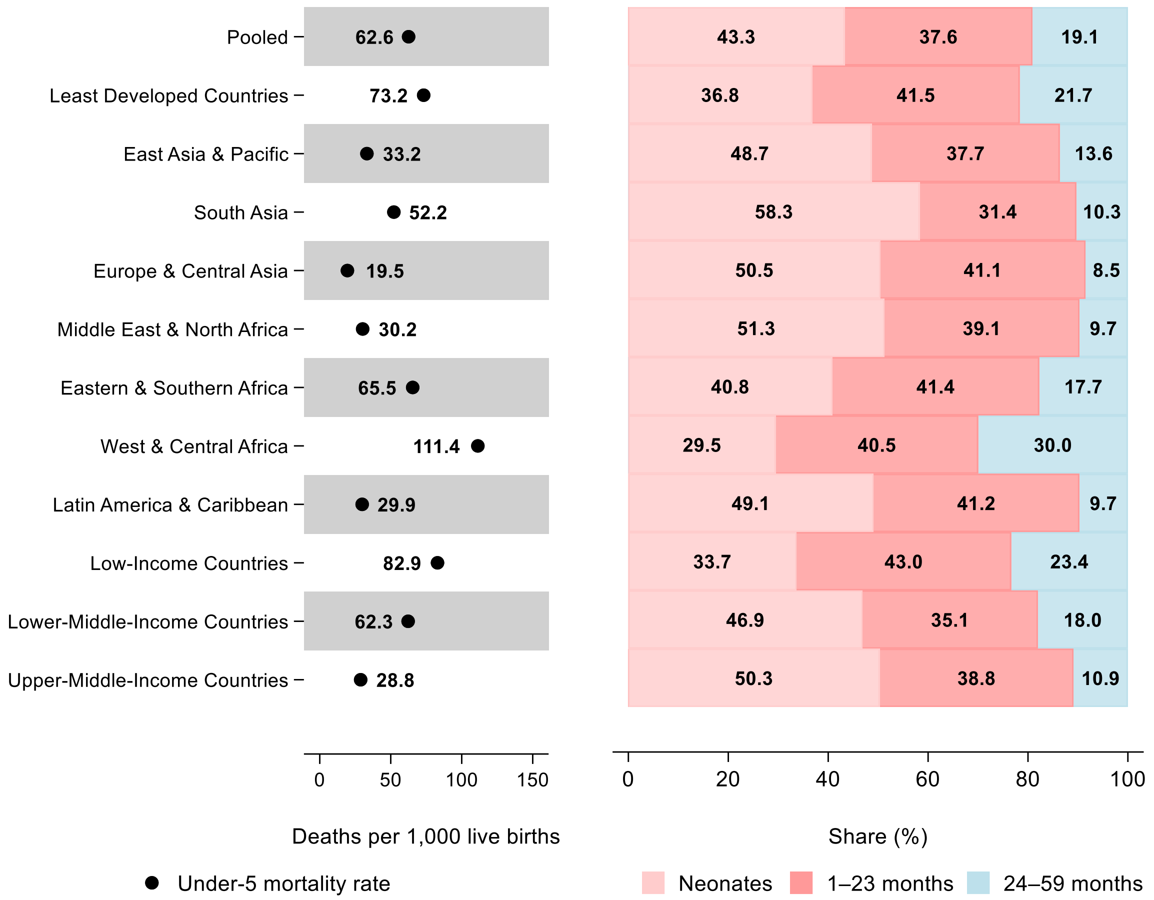

Notes: 95% confidence intervals are shown for mortality rates (some confidence intervals may be narrower than symbols). See eTable 2 in the Supplement for tabulated estimates and confidence intervals.

© 2022 Karlsson O et al. *JAMA Network Open*.

eFigure 2. Share of Deaths Among Children Younger Than 5 Years Occurring at Different Ages Using Detailed Age Intervals: Pooled, Least Developed Countries, Regions, and World Bank Income Groups: Period Restricted to 5 Years Before Survey Instead of 10 Years

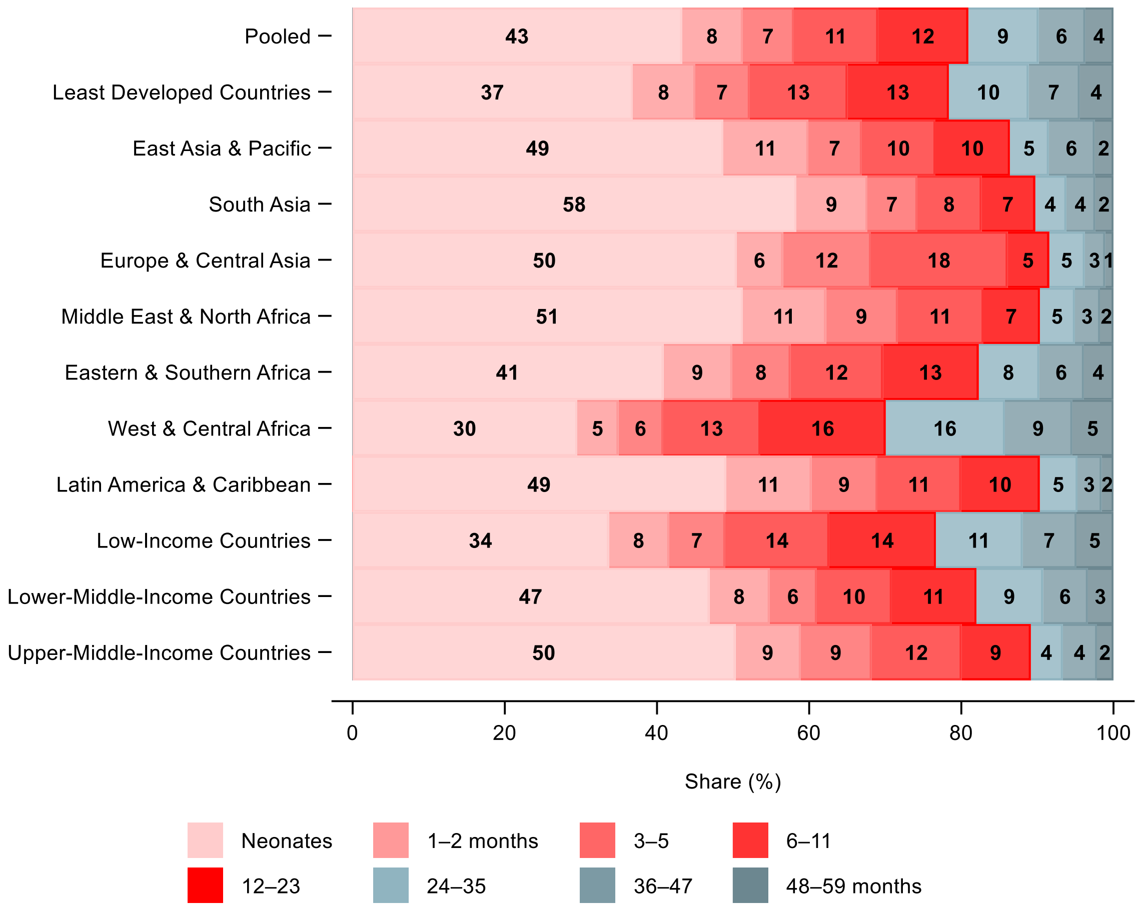

Notes: See eTable 3 in the Supplement for tabulated estimates and confidence intervals.

© 2022 Karlsson O et al. *JAMA Network Open*.

eFigure 3. Mortality Rate Among Children Younger Than 5 Years and Share of These Deaths Occurring at Different Ages by Country: Period Restricted to 5 Years Before Survey Instead of 10 Years

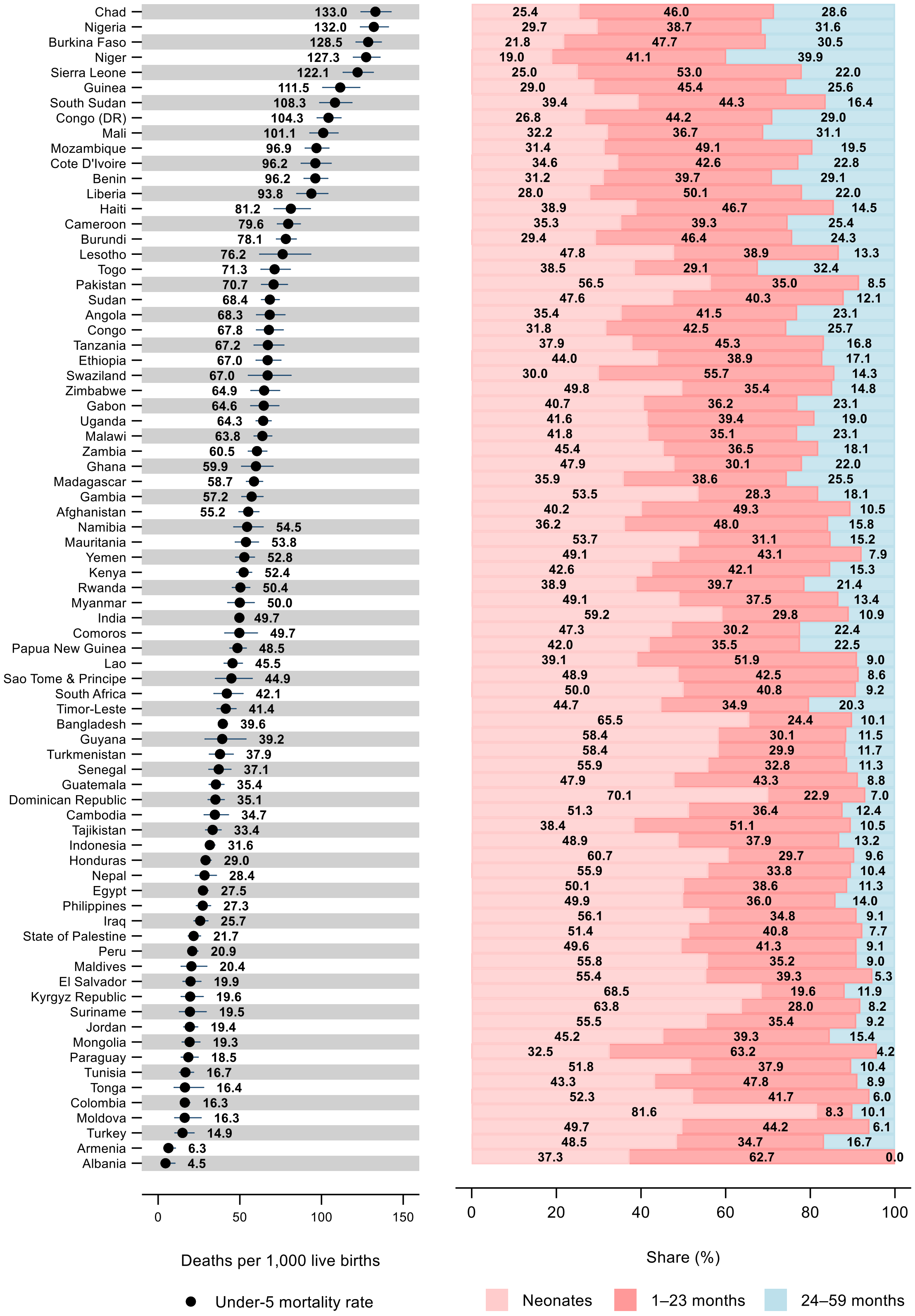

Notes: 95% confidence intervals are shown for mortality rates (some confidence intervals may be narrower than symbols). See eTable 2 in the Supplement for tabulated estimates and confidence intervals.

© 2022 Karlsson O et al. *JAMA Network Open*.

eFigure 4. Share of Deaths Among Children Younger Than 5 Years Occurring at Different Ages Using Detailed Age Intervals by Countries: Period Restricted to 5 Years Before Survey Instead of 10 Years

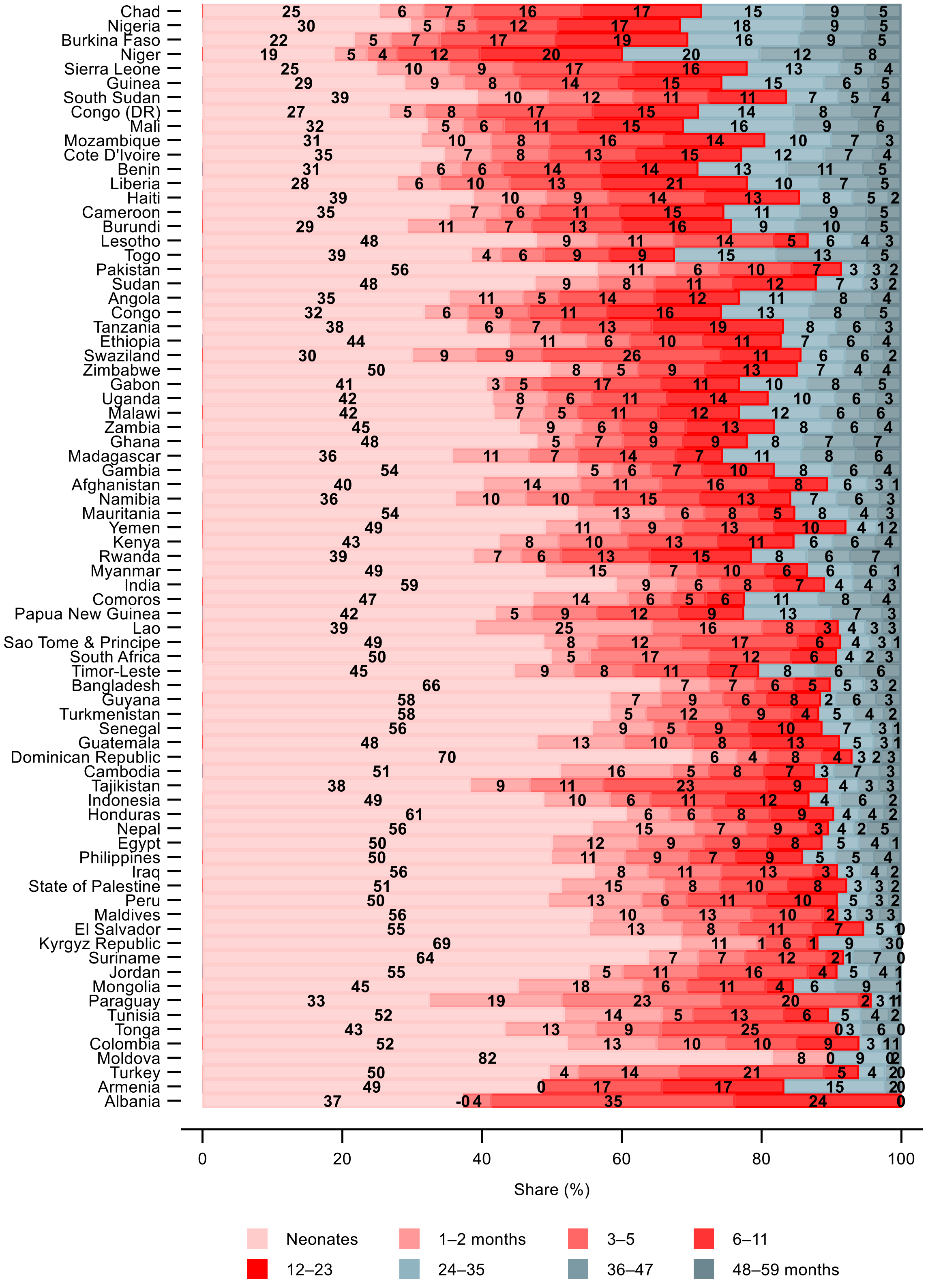

Notes: See eTable 3 in the Supplement for tabulated estimates and confidence intervals.

© 2022 Karlsson O et al. *JAMA Network Open*.

eFigure 5. Lexis Diagram Demonstrating Inclusion for Each Component Mortality Probability

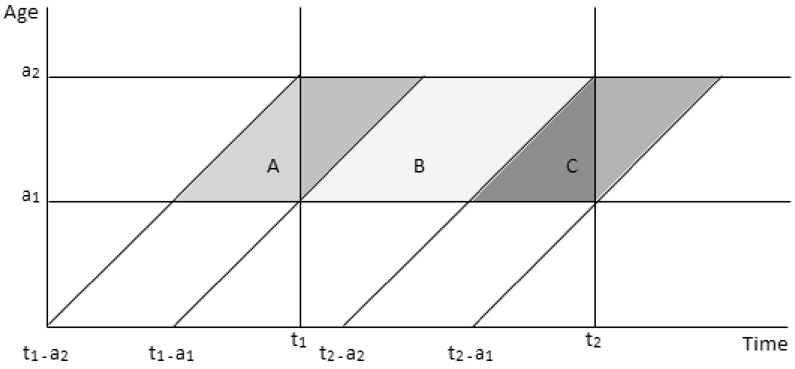

Source: Croft, Trevor N., Aileen MJ Marshall, and Courtney K. Allen. 2018. Guide to DHS Statistics DHS-7 (Version 2). Rockville, Maryland, USA: ICF.

© 2022 Karlsson O et al. *JAMA Network Open*.

eFigure 6. Country-Level Association Between Mortality Rate Among Children Younger Than 5 Years and Share of These Deaths Occurring at Different Ages

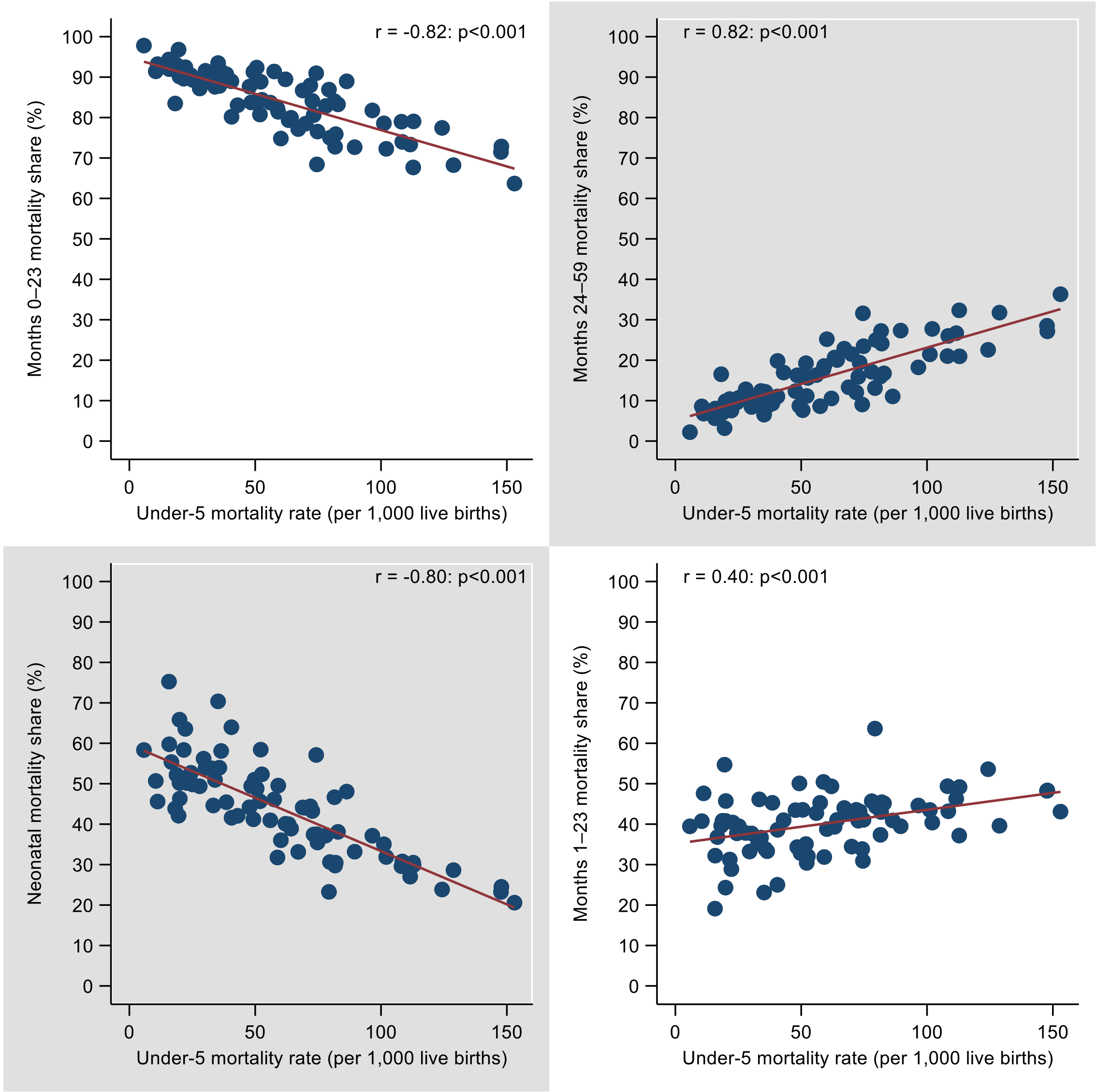

Notes: Pearson's correlation coefficients (r) are shown.

© 2022 Karlsson O et al. *JAMA Network Open*.

eFigure 7. Share of Deaths Among Children Younger Than 5 Years Occurring at Different Ages Using Detailed Age Intervals by Countries

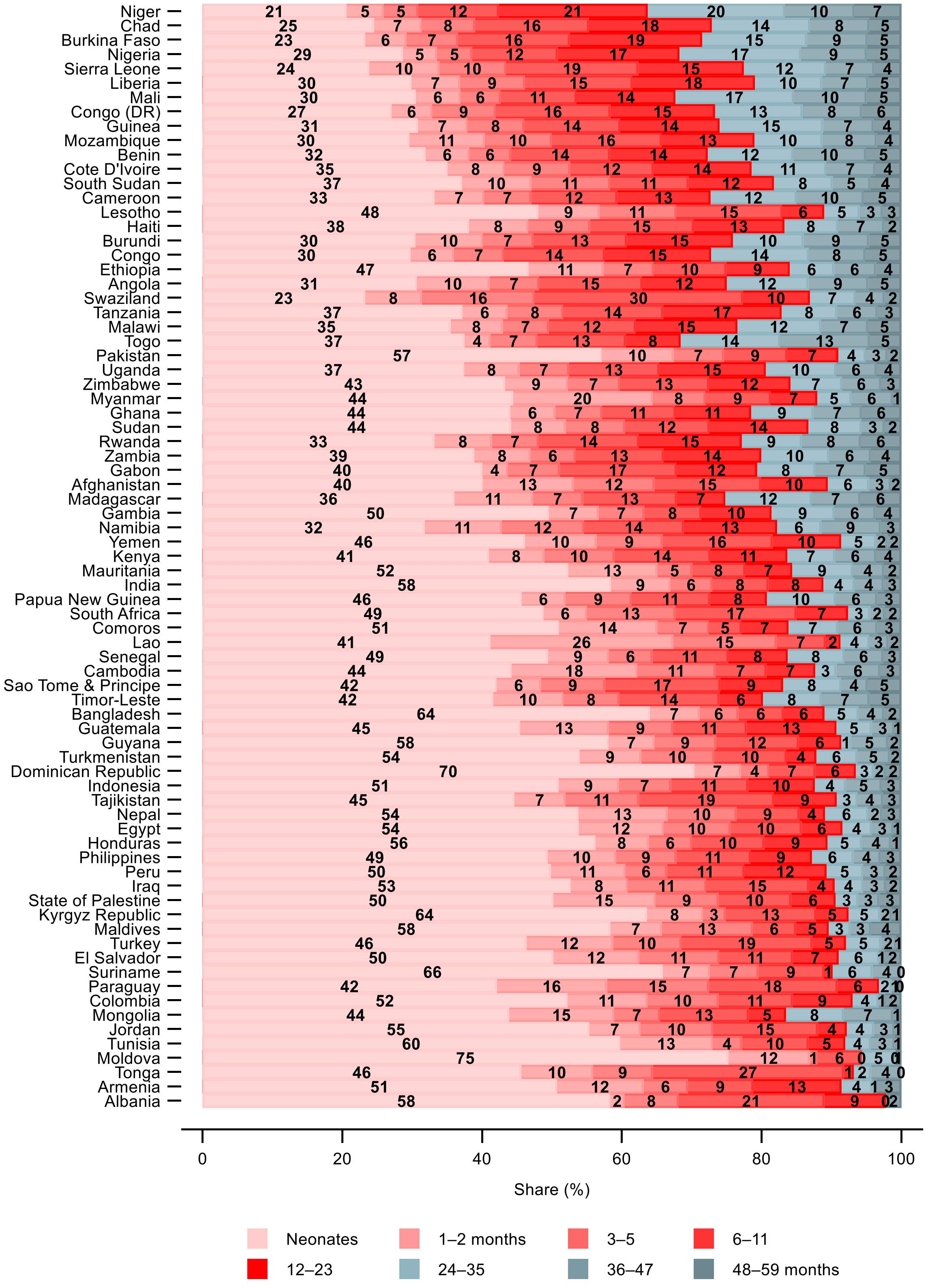

Notes: See eTable 6 in the Supplement for tabulated estimates and confidence intervals.

© 2022 Karlsson O et al. *JAMA Network Open*.

eFigure 8. Share of Deaths Among Children Younger Than 5 Years Occurring at Different Ages: Countries by Living Standards

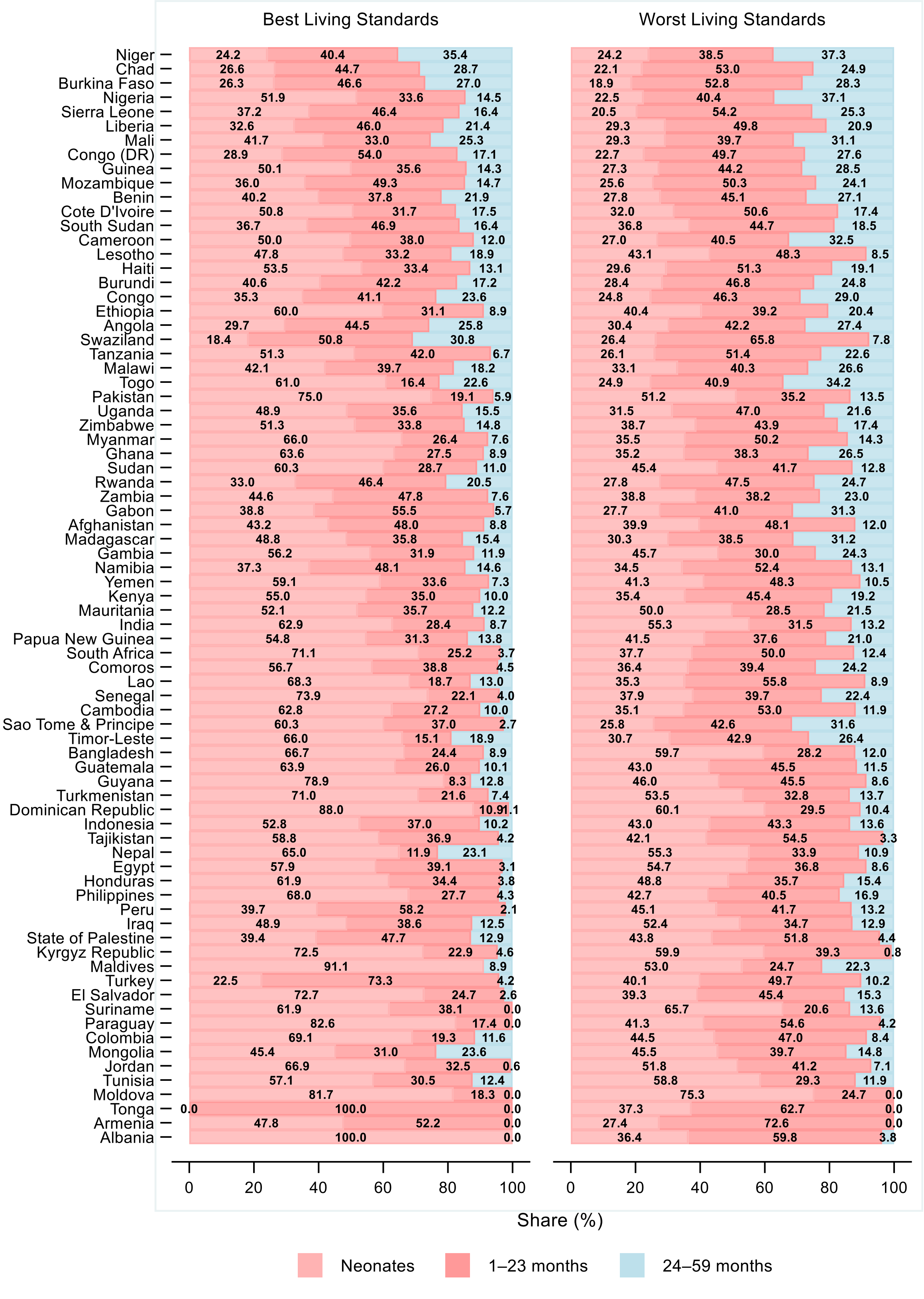

Notes: See eTable 8 in the Supplement for tabulated estimates and confidence intervals. 'Worst' refers to children in the 20% of households with the worst living standards and 'Best' refers to children in the 20% of households with the best living standards, within each country.

© 2022 Karlsson O et al. *JAMA Network Open*.

eFigure 9. Share of Deaths Among Children Younger Than 5 Years Occurring at Different Ages: Countries by Sex

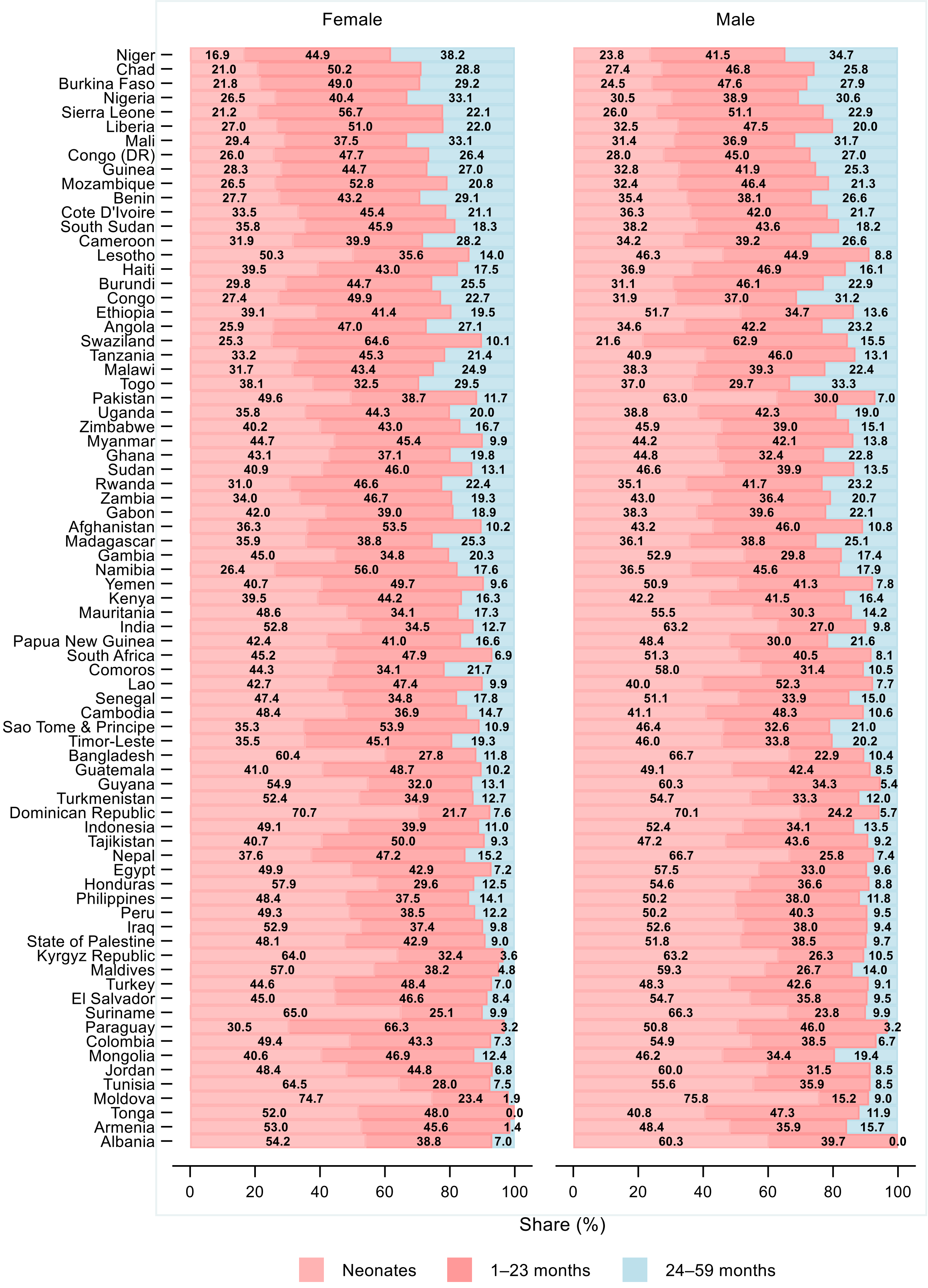

Notes: See eTable 10 in the Supplement for tabulated estimates and confidence intervals.

© 2022 Karlsson O et al. *JAMA Network Open*.

eReferences

1 Croft T. DHS Data Editing and Imputation. DHS 1991. https://dhsprogram.com/pubs/pdf/DHSG3/DHS_Data_Editing.pdf
2 UN. Mortality estimates from major sample surveys: towards the design of a database for the monitoring of mortality levels and trends. United Nations, Department of Economic and Social Affairs, Population Division 2011. https://www.un.org/en/development/desa/population/publications/pdf/technical/TP2011-2_MortEstMajorSampSurv.pdf
3 Rutstein S. Infant and child mortality: levels, trends, and demographic differentials. Revised edition. WFS Comparative Studies No. 43. In: *International Statistical Institute*. 1984.
4 Somoza JL. Illustrative Analysis, Infant and Child Mortality in Colombia. International Statistical Institute 1980.
5 Croft TN, Marshall AM, Allen CK, *et al.* Guide to DHS statistics. *Rockv Md USA ICF* 2018.
6 Mahy M. Childhood mortality in the developing world: A review of evidence for the Demographic and Health Surveys. Calverton, Maryland, USA: : ORC Macro 2003. http://dhsprogram.com/pubs/pdf/CR4/CR4.pdf
7 DHS. The DHS Program - Research Topics - Wealth Index. 2021. http://www.dhsprogram.com/topics/wealth-index/Index.cfm (accessed 5 May 2021).

© 2022 Karlsson O et al. *JAMA Network Open*.
